# A loss-of-function genetic screening identifies novel mediators of thyroid cancer cell viability

**DOI:** 10.18632/oncotarget.8577

**Published:** 2016-04-04

**Authors:** Maria Carmela Cantisani, Alessia Parascandolo, Merja Perälä, Chiara Allocca, Vidal Fey, Niko Sahlberg, Francesco Merolla, Fulvio Basolo, Mikko O. Laukkanen, Olli Pekka Kallioniemi, Massimo Santoro, Maria Domenica Castellone

**Affiliations:** ^1^ IRCCS SDN, Naples, Italy; ^2^ Dipartimento di Medicina Molecolare e Biotecnologie Mediche, Universita’ Federico II, Naples, Italy; ^3^ Medical Biotechnology, VTT Technical Research Centre of Finland, Turku, Finland; ^4^ Center for Biotechnology, University of Turku, Turku, Finland; ^5^ Dipartimento di Scienze Biomediche Avanzate, Università Federico II, Naples, Italy; ^6^ Division of Pathology, Department of Surgery, University of Pisa, Pisa, Italy; ^7^ FIMM-Institute for Molecular Medicine Finland, University of Helsinki, Helsinki, Finland; ^8^ Istituto di Endocrinologia ed Oncologia Sperimentale “G. Salvatore” (IEOS), C.N.R., Naples, Italy

**Keywords:** kinases, screening, siRNA, thyroid carcinoma

## Abstract

RET, BRAF and other protein kinases have been identified as major molecular players in thyroid cancer. To identify novel kinases required for the viability of thyroid carcinoma cells, we performed a RNA interference screening in the RET/PTC1(CCDC6-RET)-positive papillary thyroid cancer cell line TPC1 using a library of synthetic small interfering RNAs (siRNAs) targeting the human kinome and related proteins. We identified 14 hits whose silencing was able to significantly reduce the viability and the proliferation of TPC1 cells; most of them were active also in BRAF-mutant BCPAP (papillary thyroid cancer) and 8505C (anaplastic thyroid cancer) and in RAS-mutant CAL62 (anaplastic thyroid cancer) cells. These included members of EPH receptor tyrosine kinase family as well as SRC and MAPK (mitogen activated protein kinases) families. Importantly, silencing of the identified hits did not affect significantly the viability of Nthy-ori 3-1 (hereafter referred to as NTHY) cells derived from normal thyroid tissue, suggesting cancer cell specificity. The identified proteins are worth exploring as potential novel druggable thyroid cancer targets.

## INTRODUCTION

Thyroid cancer is the most common endocrine malignancy, whose incidence has been steadily increasing over the past 30 years [1–3]. Most common thyroid cancer types arise from follicular epithelial cells and are classified in well-differentiated papillary (PTC) and follicular (FTC), poorly differentiated (PDC), and anaplastic (undifferentiated) (ATC) carcinoma; PTC accounts for the vast majority of thyroid cancer cases [1–3].

Oncogenic conversion of kinases involved in the MAPK (mitogen activated protein kinases) and PI3K (phosphatidyl-inositol-3-kinase)/AKT signaling cascades occurs in thyroid cancer [1–4]. Accordingly, point mutations of the BRAF serine/threonine kinase as well as rearrangements (RET/PTC) of RET receptor tyrosine kinase (RTK) or of other RTKs are found in the majority of PTC cases [1–6]. Mutations of RAS, an upstream activator of both MAPK and PI3K, are common in FTC and in follicular-variant PTC [5–7]. Mutations or copy number alterations of PI3K and AKT are found in ATC and radioiodine-refractory thyroid cancer [3, 8–10].

Unbiased RNA interference (RNAi) screenings, based on the use of siRNAs collections targeting the entire genome or relevant gene families, are being exploited to identify novel molecular mediators of tumorigenesis and therapeutic targets [11]. Here, through a siRNA-based kinome screen, we identified a set of kinases and related proteins as novel drivers of thyroid cancer cells viability.

## RESULTS

### High-throughput siRNA-based screening in TPC1 thyroid cancer cells

We designed a functional screening using the human papillary carcinoma cell line, TPC1, harboring at the endogenous level the RET/PTC1 (CCDC6-RET) rearrangement [12] and a commercially available siRNA library containing synthetic siRNAs targeting the 518 human protein kinases as well as kinase-related and -associated proteins. The library included two independent siRNAs targeting each transcript and negative controls consisting of siRNAs targeting green fluorescent protein (GFP), eight non targeting scrambled sequences, as well as buffer alone. The screening was performed in duplicate. Seventy-two hours after transfection, viable cells were stained and antiproliferative hits were identified as genes whose knock-down, by at least one of the two siRNAs, reduced (by at least 30%; loess-log ≤-0.5) cell viability in both replicates upon normalization to the median value of negative controls. A plot of the results is shown in Supplemental Information, Figure S1 and raw data are available in Supplemental Information, Table S1. Overall, silencing of 50 genes reduced TPC1 cell viability. This list included a pseudogene LOC392265 and a Ser/Thr-like kinase MGC44796 (later identified as STK40) that were excluded from further studies (Supplemental Information, Table S2).

Though TPC1 cells depend on RET/PTC1 for their viability [12], RET was not present among the hits because the two RET targeting siRNAs included in our library did not efficiently knock-down the RET/PTC1 expression (data not shown).

### Validation of antiproliferative hits in TPC1 cells

**Figure 1:**
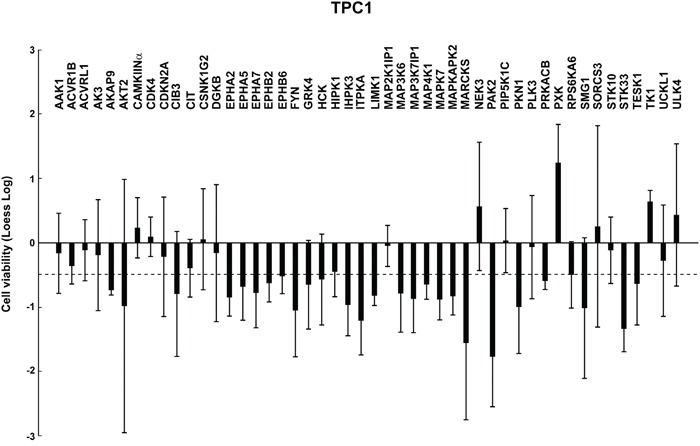
Identification of genes essential for TPC1 cell viability TPC1 cells were transfected in duplicate with 4 independent siRNAs (Supplemental Information, Table S2) targeting different regions of the 48 antiproliferative hits. Seventy-two hours after transfection, cell viability was measured by CellTiter-Blue assay and expressed as log2 (loess-log normalization). The reported values are the average results of the transfection of 4 siRNAs for each gene in duplicate normalized by negative controls (average results of Non specific siRNA Control and AllStars Negative siRNA Control). The bars represent 95% confidence intervals. Dotted line represents an arbitrary cut-off value (loess-log ≤-0.5) applied to select the down hits.

**Figure 2:**
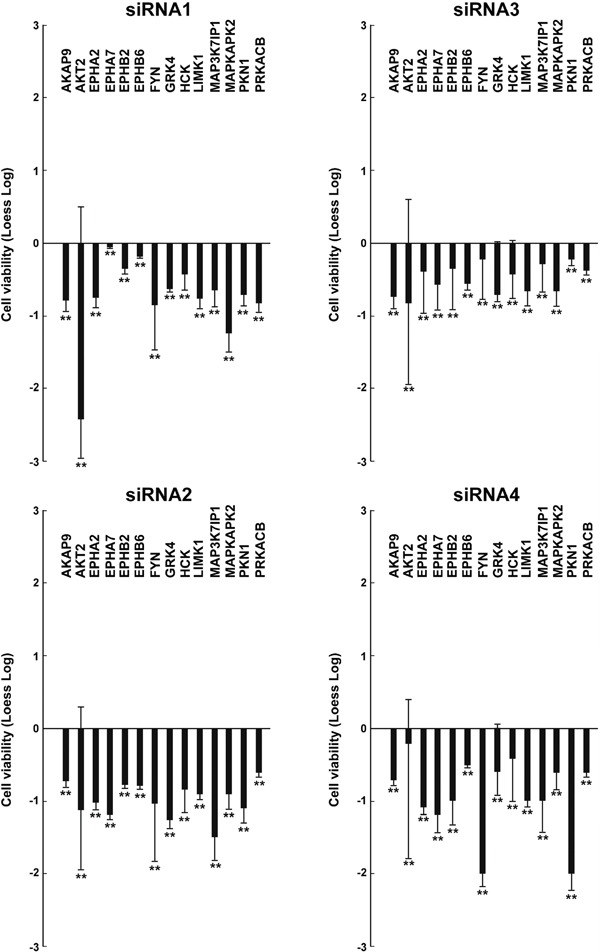
Effects on cell viability of different siRNAs in TPC1 cells TPC1 cellswere transfected in triplicate with 4 independent siRNAs (listed in Supplemental Information, Table S2) targeting different regions of the 14 antiproliferative hits. Seventy-two hours after transfection, cell viability was measured by CellTiter-Blue assay and expressed as log2 (loess-log normalization). The reported values are the average results of the transfection of 4 siRNAs for each gene in triplicate normalized by the scrambled control (average value of Non specific siRNA Control and AllStars Negative siRNA Control). The bars represent 95% confidence intervals. Significance was calculated by a paired, two-tailed Student's t test; **: *p* ≤0.01.

To confirm the results of the screening, we measured TPC1 cell viability upon transfection with the same 2 siRNAs from the library (siRNA1 and 2) as well as with 2 independent siRNAs (siRNA3 and 4) targeting a distinct mRNA region of the 48 hits (Supplemental Information, Table S2); average results are reported in Figure 1 and raw data are shown in Supplemental Information, Table S3. Nine out of 48 genes were not confirmed to reduce cell viability and were therefore excluded from further tests. Among the remaining ones, silencing of 27 genes reduced dye incorporation by at least 30% (loess-log ≤-0.5) (average value of the 4 different siRNAs) as compared to the negative controls (siRNAs with no homology to any known mammalian gene: Non specific siRNA Control, catalogue no. 1022079 and AllStars Negative siRNA Control, catalogue no. SI03650318, Qiagen); in particular, in the case of 20 genes the reduction was significant (p <0.05) with each one of the four different used siRNAs. We therefore restricted our analysis to these 20 hits and tested effects of their silencing in a control cell line (NTHY) obtained from normal thyroid tissue and immortalized by SV40 Large T. Silencing of six (CIB3, ITPKA, MAPK7, MARCKS, PAK2, STK33) genes affected the viability also of control cells (data not shown) and therefore they were excluded from further tests, thus finally resulting in 14 selected hits (Table 1 and Figure 2).

**Table 1:** List of the 14 antiproliferative hits identified in TPC1 cells

Symbol	Gene name	Genbank ID
**AGC: cAMP-dependent, cGMP-dependent and protein kinase C**		
AKT2	v-akt murine thymoma viral oncogene homolog 2	NM_001626
PKN1	protein kinase N1	NM_002741
PRKACB	protein kinase cAMP dependent, catalitic, beta	NM_182948
**CMGC group**		
AKAP9	A kinase (PRKA) anchor protein (yotiao) 9	NM_005751
MAP3K7IP1	mitogen-activated protein kinase kinase kinase 7 interacting protein 1	NM_006116
**GPCR coupled kinases**		
GRK4	G protein-coupled receptor kinase 4	NM_182982
**STE: serine/threonine kinase family**		
MAPKAPK2	mitogen-activated protein kinase-activated protein kinase 2	NM_004759
**TK: tyrosine kinases**		
EPHA2	EPH receptor A2	NM_004431
EPHA7	EPH receptor A7	NM_004440
EPHB2	EPH receptor B2	NM_017449
EPHB6	EPH receptor B6	NM_004445
FYN	FYN oncogene	NM_002037
HCK	hemopoietic cell kinase	NM_002110
**TKL: tyrosine kinase like**		
LIMK1	LIM domain kinase 1	NM_002314

### Validation of antiproliferative hits in a panel of thyroid cancer cell lines

**Figure 3:**
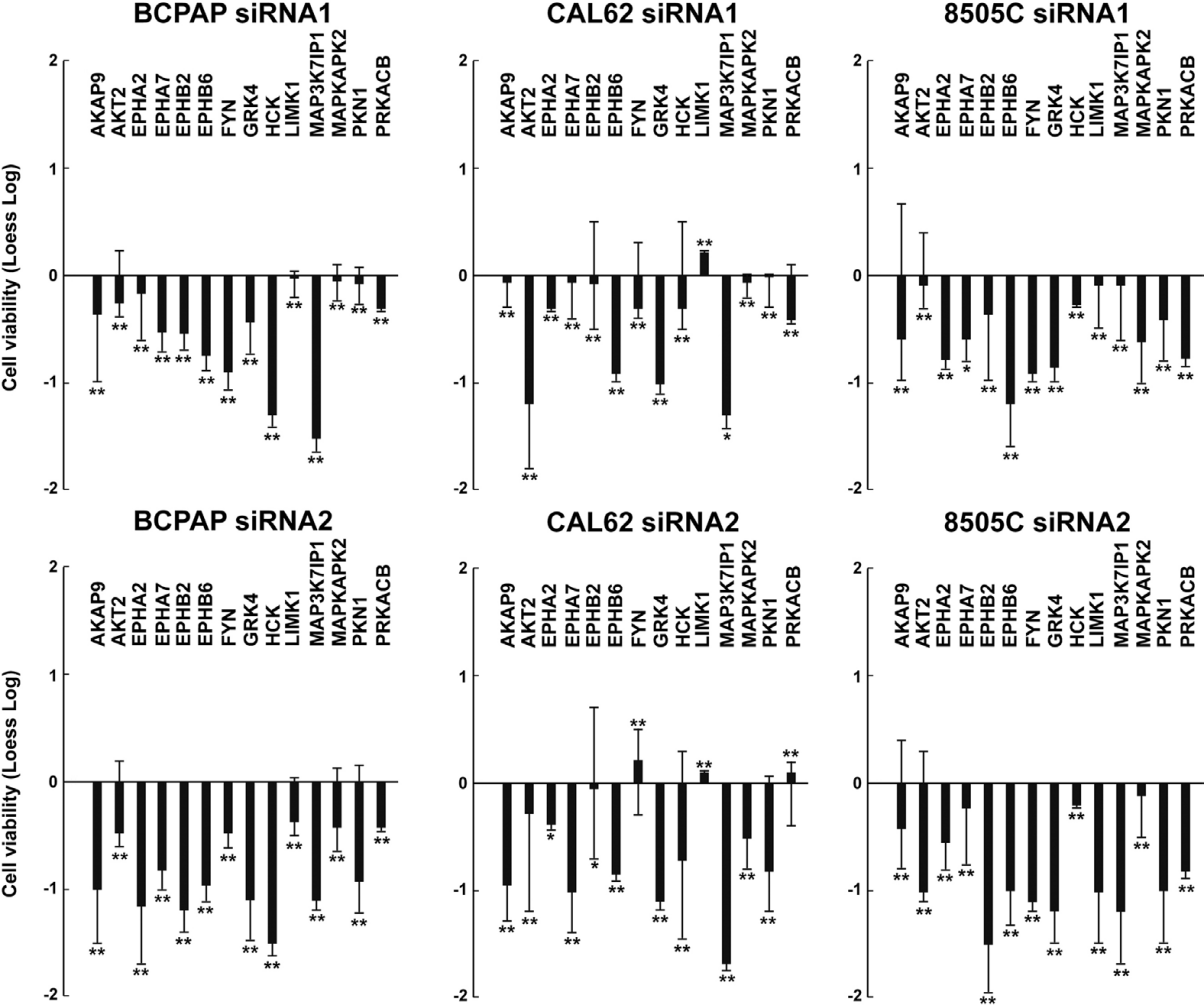
Effects on cell viability of silencing of the 14 antiproliferative hits in independent thyroid cancer cell lines BCPAP, CAL62, and 8505C cellswere transfected in triplicate with 2 siRNAs (siRNA1 and siRNA2) targeting different regions of the 14 antiproliferative hits. Seventy-two hours after transfection, cell viability was measured by CellTiter-Blue assay and expressed as log2 (loess-log normalization). The reported values are the average results of the transfection of 2 siRNAs for each gene in triplicate normalized by the scrambled control (AllStars Negative siRNA Control). The bars represent 95% confidence intervals. Significance was calculated by a paired, two-tailed Student's t test; *: *p* ≤0.05; **: *p* ≤0.01.

**Figure 4:**
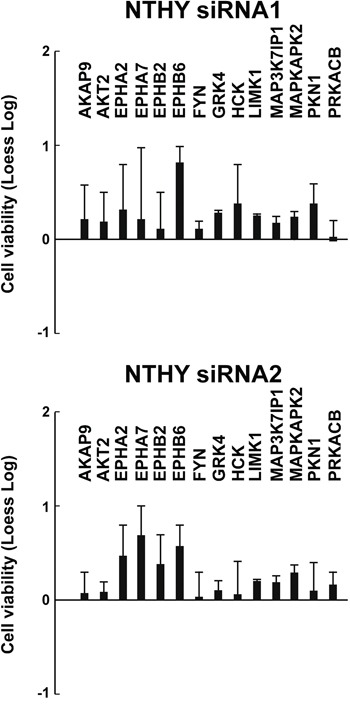
Effects on cell viability of silencing of the 14 antiproliferative hits in NTHY cells NTHY cellswere transfected in triplicate with 2 independent siRNAs (siRNA1 and siRNA2) targeting different regions of the 14 antiproliferative hits. Seventy-two hours after transfection, cell viability was measured by CellTiter-Blue assay and expressed as log2 (loess-log normalization). The reported values are the average results of the transfection of 2 siRNAs for each gene in triplicate normalized by the scrambled control (AllStars Negative siRNA Control). The bars represent 95% confidence intervals. Significance was calculated by a paired, two-tailed Student's t test.

We sought to test whether effects of the 14 antiproliferative hits were restricted to TPC1 or common to other thyroid cancer cell lines driven by oncogenes different from RET. To this aim, we selected 3 additional thyroid cancer cell lines (BCPAP, 8505C and CAL62) of different histotypes and featuring different complements of genetic lesions. BCPAP and 8505C cells bear a BRAF V600E and CAL62 bears a KRAS G12R mutation, respectively (Supplemental Information, Table S4) [13]. The 14 hits were individually silenced by siRNA1 and 2 in the three cancer cell lines, compared to NTHY cells. Silencing of most of them (14/14 genes for BCPAP, 11/14 for CAL62 and 14/14 for 8505C cells) significantly (*p* <0.01) reduced viability of cancer (Figure 3) but not NTHY (Figure 4) cells. Exceptions were represented by FYN and PRKACB that reduced CAL62 cell viability only when silenced with siRNA1 and LIMK1 that did not reduce dye incorporation with either siRNA.

**Figure 5:**
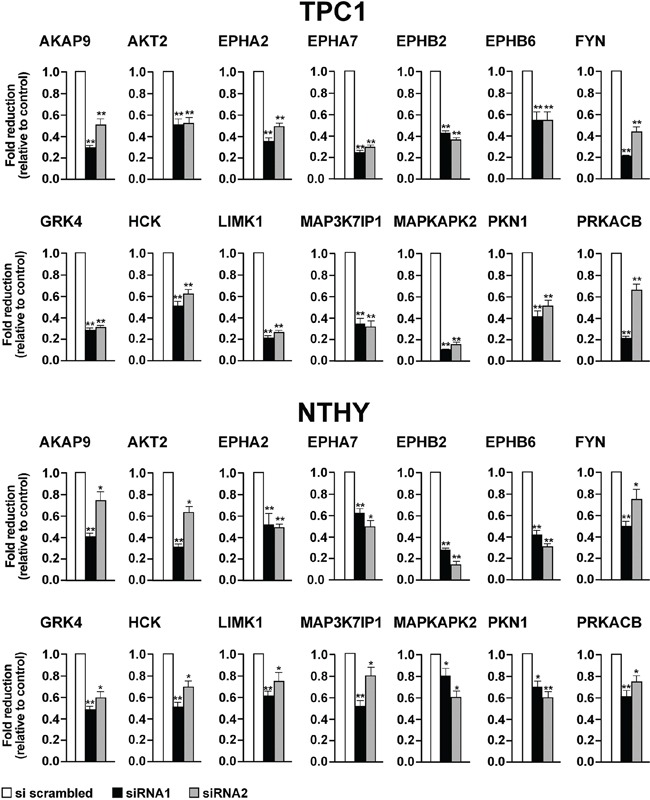
Effects on mRNA levels exerted by silencing of the 14 selected antiproliferative hits TPC1 **A.** and NTHY **B.** were transiently transfected with siRNA1 and siRNA2. After 72 hours, mRNA levels of the siRNA targets were measured by quantitative RT-PCR. Fold changes for each target (dark bars) were calculated with the formula: 2^−(gene of interest ΔCt - control ΔCt)^, where ΔCt is the difference between the amplification fluorescent threshold of the mRNA of interest and the mRNA of Δ-actin used as an internal reference. Control (white bars) was represented by scrambled siRNA (AllStars Negative siRNA Control) and was arbitrarily set at 1.0. Average results of three independent PCR reactions with upper 95% confidence intervals are reported. Significance was calculated by a paired, two-tailed Student's t test; *: *p* ≤0.05; **p ≤0.01.

To confirm gene knock-down, we performed quantitative RT-PCRs in TPC1 and NTHY cells transiently transfected with siRNA1 and siRNA2 for the 14 selected hits (Supplemental Information, Table S5). In all the cases, mRNA targets were significantly (p <0.05) depleted by cognate siRNAs with respect to negative control (AllStars Negative Control siRNA) (Figure 5).

### Antiproliferative effect of the 14 hits in thyroid cancer cells

**Figure 6:**
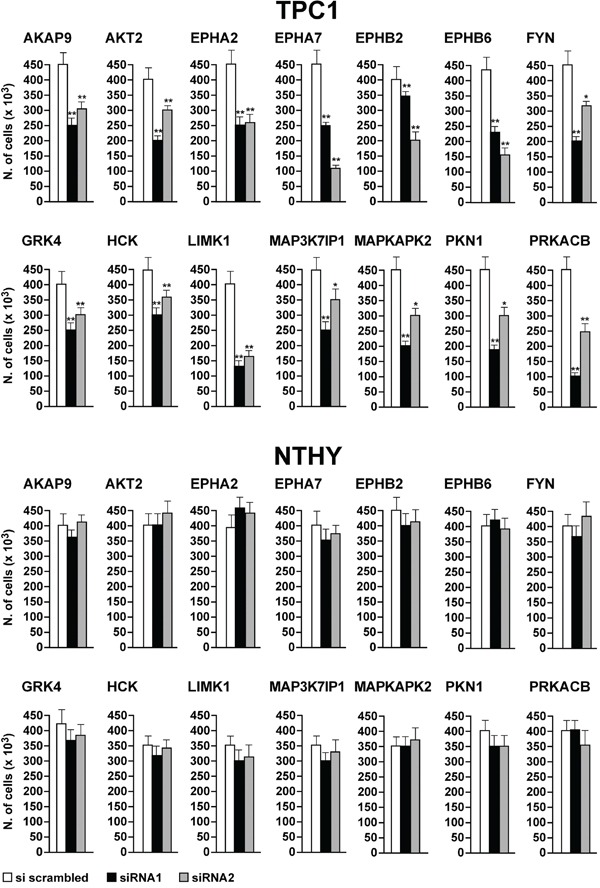
Reduction of cell number by silencing of the 14 antiproliferative hits TPC1 **A.** and NTHY **B.** were transfected in triplicate with siRNA1 and 2. Seventy-two hours after transfection, cells were counted. Average cell counts upon the transfection of the 2 siRNAs in triplicate are reported (dark bars). Control was represented by scrambled siRNA (AllStars Negative siRNA Control) (white bars). Upper 95% confidence intervals are shown. Significance was calculated by a paired, two-tailed Student's t test; *: *p* ≤0.05; **: *p* ≤0.01.

**Figure 7:**
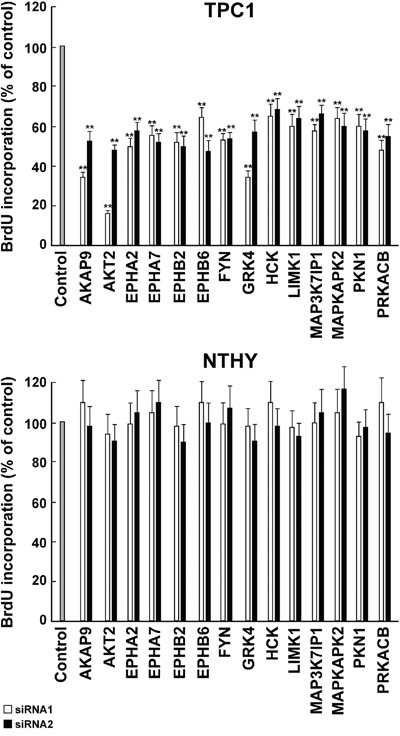
Inhibition of DNA synthesis by silencing of the 14 antiproliferative hits TPC1 **A.** and NTHY **B.** cells were transfected in triplicate with 2 independent siRNAs (siRNA1 and 2, Supplemental Information, Table S2) targeting the 14 antiproliferative hits. Seventy-two hours after transfection, cell were pulsed with BrdU for 2 hours, fixed and BrdU incorporation was detected by a peroxidase-conjugated anti-BrdU antibody. Results were read by a microplate reader and calculated as % reduction with respect to the negative control represented by scrambled siRNA (AllStars Negative siRNA Control). Average results of the transfection of individual siRNA in triplicate for each gene are reported. The bars represent 95% confidence intervals. Significance was calculated by a paired, two-tailed Student's t test; **: *p* ≤0.01.

To better characterize the biological effect of the knock-down of the 14 hits, we performed cell count and DNA synthesis measurement (BrdU assay). Transfection of siRNA1 and 2 against all of the 14 hits significantly (*p* <0.05) decreased cell number (Figure 6, upper) and BrdU incorporation (Figure 7, upper) in TPC1 but not in NTHY cells (Figure 6–7, lower).

**Figure 8:**
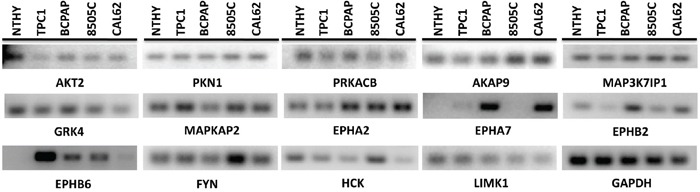
Expression level of the 14 identified hits in a panel of normal and thyroid cancer cell lines The level of expression of the 14 antiproliferative hits was tested by semiquantitative RT-PCR in normal (NTHY) and cancer (TPC1, BCPAP, 8505C and CAL62) thyroid cell lines. GAPDH is shown for normalization.

Finally, we asked whether differential expression levels can account for the differential effect observed in cancer with respect to control NTHY cells. Only minor differences in the expression levels of the 14 genes were observed when tested by semiquantitative RT-PCR in the panel of cell lines used for this study, with the exception of EPHA7, EPHB2 and EPHB6 that resulted highly expressed in some cancer cell lines compared to NTHY cells (Figure 8). Moreover, even genes with a low expression level had robust effects on cell viability and proliferation (Figure 8).

## DISCUSSION

Here, we applied a RNAi-based loss-of-function screen to identify protein kinases (and kinase-related proteins) required for viability of thyroid cancer cells. We focused on protein kinases because of their frequent involvement in thyroid cancer [1–6] and their “druggability” [13, 14]. We identified a set of 14 antiproliferative hits that, when silenced, impaired the viability of various thyroid cancer cell lines. Importantly, though the screening was initially conducted on a RET/PTC1-positive cell line (TPC1), subsequent validation experiments proved that sensitivity to knock-down of the 14 genes was shared by most thyroid cancer cell lines harboring different oncogenic lesions (BRAF mutation in BCPAP and 8505C and KRAS mutation in CAL62). This is noteworthy because BRAF mutation is recognized as risk factor for thyroid cancer progression [1, 3] and because, so far, KRAS proteins have proved very difficult to be directly targeted [15]. P53 mutation is a marker of aggressive thyroid cancers [1, 3] and effects of siRNA-mediated kinase knock-down were not dependent on functional P53 because some of the used cell lines were P53-mutated (Supplemental Information, Table S4). Finally, immortalized thyrocytes coming from non tumoral tissue were not sensitive to knock-down of the 14 hits; a fact that warrants a therapeutic window for approaches aimed at inhibiting these hits in thyroid cancer.

Overall, the 14 hits list included 3 cytosolic tyrosine kinases, 4 receptor tyrosine kinases (all belonging to the EPH family), and 7 serine/threonine kinases. This list was enriched for: i) EPH receptors (EPHA2, EPHA7, EPHB2, EPHB6); ii) SRC family kinases (FYN, HCK); iii) kinases belonging to the p38 or JNK signaling cascades (MAP3K7IP1, MAPKAPK2 and PKN1); iv) proteins of PI3K/mTOR signaling (AKT2) or the PKA/cyclic AMP pathway (PRKACB, AKAP9) (Table 1). Some of these proteins were previously involved in thyroid cancer, such as: AKAP9, that was rearranged with BRAF in radiation-associated PTC [16], GRK4, that was overexpressed in thyroid nodules [17], EPHA2, that was overexpressed in thyroid cancer [18, 19], LIMK1 that is targeted by an onco-miR (miR-20a) in thyroid cancer cells [20] and AKT2 that was found upregulated in thyroid cancer and involved in transgenic mouse models of thyroid cancer [21–24]. Thus, most of the identified hits are involved in pathways linked to thyroid cancer: SRC family kinases have been previously involved in RET/PTC signal transduction [25] and in thyroid cancer cell viability [26], JNK and p38MAPK have been found to be activated by RET oncogenes [27, 28] and p38MAPK targeting has been described to effectively reduce proliferation of TPC1 cells [29]. Finally, a screening from radiation related PTCs identified, among other genes, also the upregulation of PRKAA1, PDLIM1 and MAPKAPK3 that are close relatives of PRKACB, LIMK1 and MAPKAPK2 identified in our screening [30].

Among the antiproliferative hits, the largest group (4/14) was represented by tyrosine kinase receptors of the EPH family. EPH receptors are divided in two families: EPHAs that preferentially bind to GPI-linked ephrin A (EFN-A) ligands, and EPHB that bind transmembrane ephrin B (EFN-B) ligands. EPH-EFN complexes emanate bidirectional signaling: forward signals that depend on EPH tyrosine-kinase activity, and reverse signals depending on SRC family kinases associated to the cytosolic side of EFN [31]. EPHs signaling controls many functions including cell migration, invasion, proliferation and survival. These activities in cancer are complex and dichotomic, pro-tumorigenic in some instances and anti-oncogenic in others [31]. For instance, EPHA2 overexpression causes oncogenic transformation of mammary epithelial cells [32, 33], was found essential for tumour growth [34, 35] and able to mediate resistance to BRAF kinase inhibitors in melanoma [36]. Noteworthy, EPHA4 is expressed in follicular cell progenitors and it is important for normal thyroid development since its ablation caused histological alterations of developing thyroid gland [37]. Moreover, functional interactions between RET, EPHA4 and EFN in motor neurons have been reported [38, 39]. In addition, enhanced EphB6 expression has been detected to be more frequently observed in malignant compared to benign thyroid lesions [40]. Enhanced EphB6 expression was significantly associated with larger tumor size, the presence of lymph node metastases, the presence of capsular, lymphatic and vascular invasion and increased risk of recurrence. In a similar way, EPHA7 has been involved in tumor growth and progression of medulloblastoma and glioblastoma multiforme (GBM) [41, 42] as well as in NSCLC [43]. Depletion of EphA7 has remarkably inhibited the proliferation and invasion in human laryngeal cancer cell lines [44]. On the other hand, EPHA7 has been identified as a soluble tumor suppressor for follicular lymphoma [45]. Finally, overexpression of EPHB2 has been detected in human cholangiocarcinoma (CCA), cutaneous squamous cell carcinoma (cSCC) and cervical cancer where it regulates an EMT program through R-Ras activation [46–48] while EPHB2 cytosolic localization has been shown to predict poor survival in breast cancer patients [49]. Interestingly, EPHB2 has been shown to have a pro-invasive role also in other tumor models, such as in GBM, where on the other side it shows anti-proliferative actions, suggesting a dichotomic role. A tumor suppressor role for EPHB2 through induction of autophagic cell death via concomitant activation of the ERK1/2 and PI3K pathways has also been described [50].

Oncogenic kinase targeting, either with monoclonal antibodies or small molecule kinase inhibitors, has emerged as a promising strategy for different human cancer types [14]. Thyroid cancer patients are treated by thyroidectomy and ablation of residual tissue with radioactive iodine. However, thyroid cancers may concentrate iodine poorly as the disease becomes less differentiated, thus limiting radioiodine efficacy [51, 52]. Antiproliferative hits identified through this screen may be evaluated as potential targets for the treatment of thyroid cancer.

## MATERIALS AND METHODS

### Cell cultures

We used thyroid cancercells featuring different genetic lesions (Supplemental Information, Table S4). TPC1 and BCPAP cell lines were derived from papillary thyroid cancer (PTC), while CAL62 and 8505C were derived from anaplastic thyroid cancer (ATC) [53]. Cells were authenticated by SNP genotyping (BMR Genomics, Padova, Italy) and were grown in Dulbecco's Modified Eagle's Medium (DMEM) supplemented with 10% Foetal Bovine Serum (FBS) (Invitrogen, Carlsbad, CA, USA), L-glutamine and penicillin/streptomycin (Sigma Aldrich, St Louis, MO, USA). Nthy-ori 3-1 (hereafter referred to as NTHY) (ECACC, Wiltshire, UK) is a human follicular epithelial cell line derived from a normal thyroid and has been immortalized by SV40 large T gene [54]. NTHY cells were grown in RPMI-1640 medium supplemented with 10% FBS (Invitrogen). All cells were expanded in culture and cryopreserved in multiple replicate vials.

### Primary siRNA screening

Initially, siRNA transfection conditions were set up for TPC1 cells by using the KDalert GAPDH kit (Ambion, Applied Biosystem, Carlsbad, CA, USA) that measures the silencing efficiency of GAPDH, used as a reference control. Then, for the screening, TPC1 cells were transfected with the human kinome siRNA set v2.0 (Qiagen, Germantown, MD, USA) in black, transparent bottom, 384/well plates (BD Biosciences, Heidelberg, Germany). Each well contained a specific siRNA targeting one of 709 distinct genes coding for kinases and kinase-related or -associated proteins. Two siRNAs for each target were used. Negative controls were represented by 8 different scrambled siRNAs, green–fluorescent protein (GFP) siRNA, and buffer alone. Four μl of a 0.165 micromolar siRNA solution were transferred from the stock plates. 10 μl of Optimem medium (Invitrogen) was mixed with 0.2 μl of HiPerfect cell transfection reagent (Qiagen) and after 10 minutes incubation, the transfection mix was transferred to each well using an automatic MultiDrop dispenser (Thermo Labsystems, Philadelphia, PA, USA). The cells were plated at 1,000 cells/well in a volume of 35 μl of DMEM supplemented with 2.5% FBS without antibiotics. Seventy-two hours after transfection, CellTiter-Blue reagent (CellTiter- Blue®, Promega, Madison, WI, USA) (10 μl) diluted (1:1) in culture medium was added to each well. Plates were incubated at 37°C, in 5% CO_2_ for 6 hours and then transferred to room temperature for overnight incubation, at dark. Cell viability was measured by dye reduction with an EnVision Multilabel plate reader at an excitation wavelength of 530 nm and emission wavelength of 590 nm (Perkin Elmer, Waltham, MA, USA). The raw data were smoothed using loess-correction and subsequently normalized to the median of the negative controls and log2-transformed (loess-log normalization). siRNAs reducing cell viability by ≥2SD (Standard Deviations) of the median of the controls were considered as antiproliferative hits. The screening was performed in duplicate and the hits replicated in both of the screens (loess log ≤-0.53 and ≤-0.88, respectively) were selected for further validation.

### Validation screening

An independent set of 2 siRNAs (Qiagen) for each of the 48 identified antiproliferative hits was obtained. The list of the 4 siRNAs (those of the library, siRNA 1-2, and the additional ones, siRNA 3-4) for each antiproliferative hit is provided in Supplemental Information, Table S2. TPC1 cells were transfected in duplicate in multi-wells with the 4 siRNAs for each antiproliferative hit. Negative control was represented by scrambled siRNAs with no homology to any known mammalian gene (Non specific siRNA Control, catalogue no. 1022079 and AllStars Negative siRNA Control, catalogue no. SI03650318, Qiagen). Cell viability data were obtained and normalized as in the primary screening. siRNAs effective at reducing cell viability by at least 30% (loess-log ≤-0.5) were selected for further studies.

### Quantitative RT-PCR

Each cell line was grown to 70% confluence, total RNA was extracted with RNeasy mini kit (Qiagen) and subjected to on-column DNase digestion with the RNase-free DNase set (Qiagen) according to the manufacturer's instructions. RNA (1μg) was reverse transcribed (RT) using a high-capacity reverse transcriptase kit (Quantitect Reverse® Transcription Kit, Qiagen). Primers were designed by using software available http://bioinfo.ut.ee/primer3/ and synthesized by the Ceinge Core Service Unit (Naples, Italy). The list of primers is provided in Supplemental Information, Table S5. ThecDNA amplification was performed using the GeneAmp RNA PCR Core Kit system (Qiagen) using 2.5 μl of RT product in a reaction volume of 25 μl with cycles of 42°C for 15 minutes, 99°C for 5 minutes, and 5°C for 5 minutes according to the manufacturer's instructions. Quantitative RT-PCR reactions were performed in triplicate by using the SYBR Green PCR Master mix (Applied Biosystems) and the iCycler apparatus (Bio-Rad Laboratories, Berkeley, CA, USA). Fluorescent threshold values were measured in triplicate and fold changes were calculated by the formula: 2^−(sample 1 ΔCt - sample 2 ΔCt)^, where ΔCt is the difference between the amplification fluorescent threshold (Ct) of the mRNA of interest and the Ct of the *Δ* actin mRNA used as housekeeping gene: 5′-TGCGTGACATTAAGGAGA-3′ (forward) and 5′-GCTCGTAGCTCTTCTCCA-3′ (reverse). Semiquantitative RT-PCR analysis was carried out with cDNAs generated from 1μg of total RNA. The RT-PCR exponential phase was determined on 25-30 cycles to allow semiquantitative comparison of different cDNAs using JumpStart REDTaq Reaction Mix, (Sigma). GAPDH mRNA was used as housekeeping gene: 5′-CCATCACCATCTTCCAGGAGCG-3′ (forward) and 5′-AGAGATGATGACCCTTTTGGC-3′ (reverse).

### Proliferation assays

Cell count was performed by plating 100,000 cells in 6 cm dishes and counting the cell number 72 hours after siRNA transfection. DNA replication rate was evaluated by 5′-bromo-3′-deoxyuridine (BrdU) incorporation with the Cell Proliferation ELISA BrdU (Roche, Mannheim, Germany). Briefly, cells were cultured in 96-multiwell plates with black, flat bottom (BD Biosciences) in a volume of 100 μl/well and transfected. After 72 hours, the cells were treated with BrdU labeling solution and incubated at 37°C for additional 2 hours. The cells were then fixed for 30 minutes at room temperature with 200 μl/well of FixDenat buffer, and incubated with 100 μl/well of anti-BrdU-POD (peroxidase) for 90 minutes at room temperature. After three washes, substrate solution was added, plates were kept on a shaker for 3 minutes at room temperature, and light emission was read by using a microplate luminometer with photomultiplier technology (DL Ready Berthold Technologies Centro, Beckman, Brea, CA, USA). Experiments were performed in triplicate.

### Statistical analysis

Statistical analyses were performed using a paired, two-tailed Student's t test (GraphPad Prism 3.0, GraphPad Software, San Diego, CA, USA), and differences were considered to be statistically significant at a value of *p* ≤0.05.

## SUPPLEMENTARY FIGURES AND TABLES




