# Supplementary material for: A loss-of-function genetic screening identifies novel mediators of thyroid cancer cell viability
Source: Oncotarget. 2016 Apr 4;7(19):28510–22. doi: 10.18632/oncotarget.8577 (PMC5053742; doi:10.18632/oncotarget.8577)
Supplement: Supplementary file 1 [file oncotarget-07-28510-s001.pdf]

SUPPLEMENTAL FIGURE AND TABLES

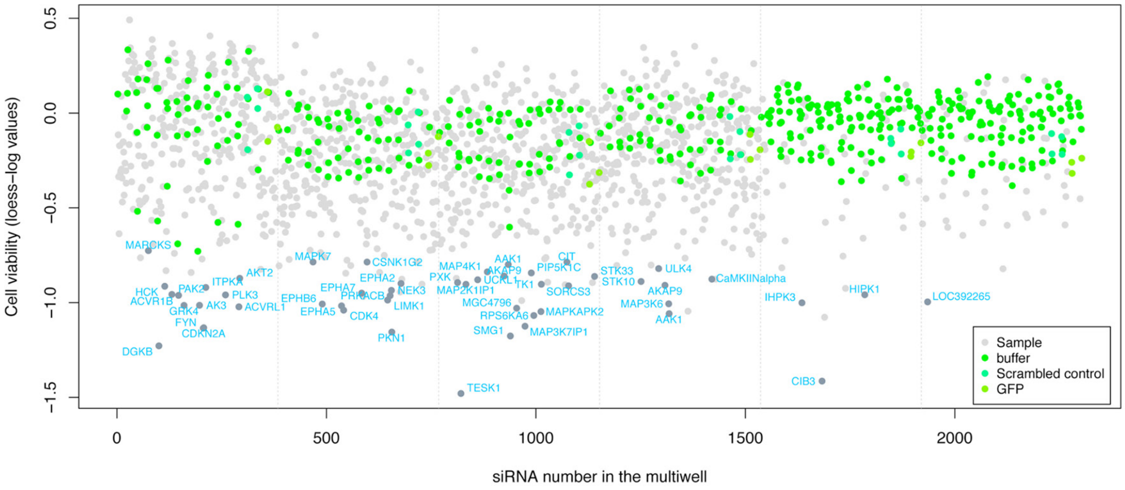

Supplementary Figure S1: Plot of screening data. Seventy-two hours after siRNA library transfection, viable TPC1 cells were measured by CellTiter-Blue reagent and antiproliferative hits (dark gray dots with gene name) were identified as genes whose knock-down, by at least one of the two siRNAs, reduced cell viability in both replicate screens upon normalization to the median value of negative controls. Loess-log plot of the results of one representative (screen 2) of the two replicated screenings is reported.Buffer, scrambled control and GFP siRNA controls are highlighted in green for performance verification. Raw data of the two screenings are reported in Table S1.

Supplementary Table S1: Screening raw data Seventy-two hours after siRNA library transfection, cell viability was measured by CellTiter-Blue assay and antiproliferative hits were identified as genes whose knock-down, by at least one of the two siRNAs, reduced cell viability in both replicate screens (loess log ≤ -0.53 and ≤ -0.88, respectively) upon normalization to the median value of negative controls. Raw data and antiproliferative hits (down) are reported separately for the two screenings (Scr1 and Scr2). siRNAs that were confirmed in both screenings were named Rep (replicated hits) and are reported in the last column.

See Supplementary File: 1

Supplementary Table S2: List of antiproliferative hits and used siRNAs

See Supplementary File: 1

Supplementary Table S3: Validation raw data (the 14 validated hits are highlighted in yellow: see text for details)

See Supplementary File: 1

Supplementary Table S4: Features of the thyroid carcinoma cell lines used in this study

See Supplementary File: 1

Supplementary Table S5: List of antiproliferative hits and qRT-PCR primers used in this study

See Supplementary File: 1
