# Supplementary material for: A loss-of-function genetic screening identifies novel mediators of thyroid cancer cell viability
Source: Oncotarget. 2016 Apr 4;7(19):28510–22. doi: 10.18632/oncotarget.8577 (PMC5053742; doi:10.18632/oncotarget.8577)
Supplement: Supplementary file 2 [file oncotarget-07-28510-s002.doc]

**Supplemental Table S1: Screening raw data**

Seventy-two hours after siRNA library transfection, cell viability was measured by CellTiter-Blue assay and antiproliferative hits were identified as genes whose knock-down, by at least one of the two siRNAs, reduced cell viability in both replicate screens (loess log ≤ -0.53 and ≤ -0.88, respectively) upon normalization to the median value of negative controls. Raw data and antiproliferative hits (down) are reported separately for the two screenings (Scr1 and Scr2). siRNAs that were confirmed in both screenings were named Rep (replicated hits) and are reported in the last column.

|  |  |  | median | 3.629.599 | 3.156.996 | -0,0212 | -0,2095 |  | |  |  |
| --- | --- | --- | --- | --- | --- | --- | --- | --- | --- | --- | --- |
|  |  |  | SD | 755.383 | 836.653 | 0,2549 | 0,3350 | threshold +/- 2 SD | | |  |
|  |  |  | median+2SD | 5.140.364 | 4.830.302 | 0,4886 | 0,4604 |  |  | |  |
|  |  |  | median-2SD | 2.118.834 | 1.483.690 | -0,5311 | -0,8794 |  |  | |  |
| **siRNA_ID** | **Symbol** | **Entrez**  **ID** | **Ensembl ID** | **raw: Scr1** | **raw: Scr2** | **loess: Scr1** | **loess: Scr2** | **Scr1**  **hits** | **Scr2**  **hits** | | **Rep hit** |
| Q006947 | AAK1 | 22848 | ENSG00000115977 | 2693383 | 2413791 | -0,6766 | -0,9375 | DOWN | DOWN | | DOWN-HIT |
| Q025023 | AAK1 | 22848 | ENSG00000115977 | 2505634 | 1741920 | -0,6787 | -1,4054 | DOWN | DOWN | | DOWN-HIT |
| Q006892 | AATK | 9625 | NA | 3461453 | 2642923 | -0,3512 | -0,4872 | -- | -- | | -- |
| Q024968 | AATK | 9625 | NA | 2822683 | 2143763 | -0,5507 | -0,7390 | DOWN | -- | | -- |
| Q006462 | ABL1 | 25 | ENSG00000097007 | 3243168 | 2123632 | 0,0965 | -0,3714 | -- | -- | | -- |
| Q024538 | ABL1 | 25 | ENSG00000097007 | 1847054 | 1733429 | -0,6113 | -0,7777 | DOWN | -- | | -- |
| Q006463 | ABL2 | 27 | ENSG00000143322 | 3399263 | 2558782 | -0,0403 | -0,3402 | -- | -- | | -- |
| Q024539 | ABL2 | 27 | ENSG00000143322 | 3691416 | 2585767 | -0,0410 | -0,4386 | -- | -- | | -- |
| Q006464 | ACVR1 | 90 | ENSG00000115170 | 4205862 | 2673843 | 0,1452 | -0,3964 | -- | -- | | -- |
| Q024540 | ACVR1 | 90 | ENSG00000115170 | 4025522 | 3214476 | -0,0828 | -0,2327 | -- | -- | | -- |
| Q006465 | ACVR1B | 91 | ENSG00000135503 | 2769151 | 1378584 | -0,5647 | -1,3972 | DOWN | DOWN | | DOWN-HIT |
| Q024541 | ACVR1B | 91 | ENSG00000135503 | 5028863 | 2996388 | 0,2025 | -0,4026 | -- | -- | | -- |
| Q006466 | ACVR2 | 92 | ENSG00000121989 | 3664789 | 2204891 | -0,1213 | -0,7062 | -- | -- | | -- |
| Q024542 | ACVR2 | 92 | ENSG00000121989 | 4586535 | 2634031 | 0,0371 | -0,6243 | -- | -- | | -- |
| Q006467 | ACVR2B | 93 | ENSG00000114739 | 4218495 | 2153810 | 0,1443 | -0,6559 | -- | -- | | -- |
| Q024543 | ACVR2B | 93 | ENSG00000114739 | 2580415 | 2272570 | -0,8952 | -0,8294 | DOWN | -- | | -- |
| Q006468 | ACVRL1 | 94 | ENSG00000139567 | 2051375 | 1196160 | -0,7599 | -1,2316 | DOWN | DOWN | | DOWN-HIT |
| Q024544 | ACVRL1 | 94 | ENSG00000139567 | 3807715 | 2890660 | -0,0728 | -0,3531 | -- | -- | | -- |
| Q007054 | ADCK1 | 57143 | ENSG00000063761 | 2796059 | 2586768 | -0,6516 | -0,5210 | DOWN | -- | | -- |
| Q025130 | ADCK1 | 57143 | ENSG00000063761 | 4110077 | 2672420 | 0,0731 | -0,4528 | -- | -- | | -- |
| Q007109 | ADCK2 | 90956 | ENSG00000133597 | 3970649 | 3380809 | 0,0161 | -0,2802 | -- | -- | | -- |
| Q025185 | ADCK2 | 90956 | ENSG00000133597 | 3414564 | 2659470 | -0,1255 | -0,6077 | -- | -- | | -- |
| Q007082 | ADCK4 | 79934 | ENSG00000123815 | 3072274 | 2874968 | -0,1978 | -0,2359 | -- | -- | | -- |
| Q025158 | ADCK4 | 79934 | ENSG00000123815 | 4124528 | 3555716 | 0,2796 | 0,1391 | -- | -- | | -- |
| Q007145 | ADCK5 | 203054 | ENSG00000173137 | 2071061 | 1971023 | -1,0701 | -0,8452 | DOWN | -- | | -- |
| Q025221 | ADCK5 | 203054 | ENSG00000173137 | 3344294 | 2491126 | -0,3359 | -0,4917 | -- | -- | | -- |
| Q006469 | ADK | 132 | ENSG00000156110 | 2700751 | 1901465 | -0,1850 | -0,5365 | -- | -- | | -- |
| Q024545 | ADK | 132 | ENSG00000156110 | 3683790 | 2194932 | 0,0563 | -0,5607 | -- | -- | | -- |
| Q006470 | ADRBK1 | 156 | ENSG00000173020 | 1657302 | 1625925 | -0,6240 | -0,6825 | DOWN | -- | | -- |
| Q024546 | ADRBK1 | 156 | ENSG00000173020 | 1895791 | 2344668 | -0,4901 | -0,3311 | -- | -- | | -- |
| Q006471 | ADRBK2 | 157 | ENSG00000100077 | 2969511 | 1939416 | -0,2291 | -0,7297 | -- | -- | | -- |
| Q024547 | ADRBK2 | 157 | ENSG00000100077 | 3590379 | 2379070 | -0,0234 | -0,4628 | -- | -- | | -- |
| Q006472 | AK1 | 203 | ENSG00000106992 | 3863748 | 1857933 | -0,0034 | -0,9611 | -- | DOWN | | -- |
| Q024548 | AK1 | 203 | ENSG00000106992 | 3215706 | 2538946 | -0,4021 | -0,4931 | -- | -- | | -- |
| Q006473 | AK2 | 204 | ENSG00000004455 | 4171728 | 3663072 | 0,0440 | -0,0324 | -- | -- | | -- |
| Q024549 | AK2 | 204 | ENSG00000004455 | 5159211 | 3332193 | 0,2694 | -0,1635 | -- | -- | | -- |
| Q006474 | AK3 | 205 | ENSG00000162433 | 2548732 | 1578276 | -0,7812 | -1,2770 | DOWN | DOWN | | DOWN-HIT |
| Q024550 | AK3 | 205 | ENSG00000162433 | 4211944 | 1819785 | -0,0829 | -1,0980 | -- | DOWN | | -- |
| Q007005 | AK3L1 | 50808 | ENSG00000147853 | 3945919 | 3259147 | 0,0496 | -0,1687 | -- | -- | | -- |
| Q025081 | AK3L1 | 50808 | ENSG00000147853 | 4644348 | 3619464 | 0,2747 | -0,0884 | -- | -- | | -- |
| Q006980 | AK5 | 26289 | ENSG00000154027 | 3985233 | 3157314 | -0,0449 | -0,2260 | -- | -- | | -- |
| Q025056 | AK5 | 26289 | ENSG00000154027 | 2746254 | 2199549 | -0,5334 | -0,7753 | DOWN | -- | | -- |
| Q007125 | AK7 | 122481 | ENSG00000140057 | 3559013 | 1499440 | 0,0397 | -0,8270 | -- | -- | | -- |
| Q025201 | AK7 | 122481 | ENSG00000140057 | 3984509 | 2616646 | 0,1734 | -0,2464 | -- | -- | | -- |
| Q006820 | AKAP1 | 8165 | ENSG00000121057 | 4769311 | 3428062 | 0,2916 | -0,0682 | -- | -- | | -- |
| Q024896 | AKAP1 | 8165 | ENSG00000121057 | 3654833 | 3397409 | 0,0252 | 0,0388 | -- | -- | | -- |
| Q006943 | AKAP10 | 11216 | ENSG00000108599 | 3360842 | 2192286 | -0,0638 | -0,5759 | -- | -- | | -- |
| Q025019 | AKAP10 | 11216 | ENSG00000108599 | 3549460 | 2633099 | 0,1044 | -0,2569 | -- | -- | | -- |
| Q006942 | AKAP11 | 11215 | ENSG00000023516 | 4395199 | 2797005 | 0,2071 | -0,3901 | -- | -- | | -- |
| Q025018 | AKAP11 | 11215 | ENSG00000023516 | 3727634 | 2833839 | 0,0593 | -0,3154 | -- | -- | | -- |
| Q006891 | AKAP12 | 9590 | ENSG00000131016 | 4262845 | 2199306 | -0,0001 | -0,7573 | -- | -- | | -- |
| Q024967 | AKAP12 | 9590 | ENSG00000131016 | 3721902 | 3028643 | -0,1042 | -0,2697 | -- | -- | | -- |
| Q006941 | AKAP13 | 11214 | ENSG00000170776 | 2777059 | 1670096 | -0,5198 | -1,2728 | -- | DOWN | | -- |
| Q025017 | AKAP13 | 11214 | ENSG00000170776 | 3838797 | 3276211 | 0,0279 | -0,2024 | -- | -- | | -- |
| Q007138 | AKAP14 | 158798 | ENSG00000186471 | 4707154 | 2974094 | 0,1956 | -0,2236 | -- | -- | | -- |
| Q025214 | AKAP14 | 158798 | ENSG00000186471 | 3884150 | 2845467 | -0,0785 | -0,2494 | -- | -- | | -- |
| Q006920 | AKAP3 | 10566 | ENSG00000111254 | 2435742 | 2527532 | -0,2905 | -0,3979 | -- | -- | | -- |
| Q024996 | AKAP3 | 10566 | ENSG00000111254 | 3144477 | 3620301 | 0,0490 | 0,0667 | -- | -- | | -- |
| Q006856 | AKAP4 | 8852 | ENSG00000147081 | 4408805 | 4118515 | 0,2051 | 0,1144 | -- | -- | | -- |
| Q024932 | AKAP4 | 8852 | ENSG00000147081 | 3849814 | 3518397 | 0,0422 | -0,1166 | -- | -- | | -- |
| Q006889 | AKAP5 | 9495 | ENSG00000179841 | 3698702 | 3207426 | -0,0230 | -0,1729 | -- | -- | | -- |
| Q024965 | AKAP5 | 9495 | ENSG00000179841 | 3549876 | 3140759 | 0,0151 | -0,1698 | -- | -- | | -- |
| Q006886 | AKAP6 | 9472 | ENSG00000151320 | 4573949 | 3522643 | 0,1738 | -0,0549 | -- | -- | | -- |
| Q024962 | AKAP6 | 9472 | ENSG00000151320 | 3828207 | 3229416 | 0,0351 | -0,0563 | -- | -- | | -- |
| Q006885 | AKAP7 | 9465 | ENSG00000118507 | 4595072 | 3757571 | 0,0985 | 0,0029 | -- | -- | | -- |
| Q024961 | AKAP7 | 9465 | ENSG00000118507 | 3372831 | 1740363 | -0,2520 | -0,9663 | -- | DOWN | | -- |
| Q006911 | AKAP8 | 10270 | ENSG00000105127 | 2527074 | 2609739 | -0,3898 | -0,3150 | -- | -- | | -- |
| Q024987 | AKAP8 | 10270 | ENSG00000105127 | 2277536 | 2095999 | -0,3896 | -0,4090 | -- | -- | | -- |
| Q006985 | AKAP8L | 26993 | ENSG00000011243 | 2924492 | 2647059 | -0,1385 | -0,2578 | -- | -- | | -- |
| Q025061 | AKAP8L | 26993 | ENSG00000011243 | 2026687 | 1767756 | -0,4309 | -0,5075 | -- | -- | | -- |
| Q024983 | AKAP9 | 10142 | ENSG00000127914 | 2161878 | 2071053 | -0,9096 | -1,0012 | DOWN | DOWN | | DOWN-HIT |
| Q006907 | AKAP9 | 10142 | ENSG00000127914 | 1986330 | 2283025 | -0,9902 | -0,6906 | DOWN | -- | | -- |
| Q007029 | AKIP | 54998 | ENSG00000175756 | 4752646 | 4056676 | 0,2367 | 0,0059 | -- | -- | | -- |
| Q025105 | AKIP | 54998 | ENSG00000175756 | 3732504 | 2975952 | -0,0618 | -0,4406 | -- | -- | | -- |
| Q006475 | AKT1 | 207 | ENSG00000142208 | 2537544 | 2236087 | -0,7116 | -0,6646 | DOWN | -- | | -- |
| Q024551 | AKT1 | 207 | ENSG00000142208 | 3740136 | 2855010 | -0,2016 | -0,3985 | -- | -- | | -- |
| Q006476 | AKT2 | 208 | ENSG00000105221 | 2281774 | 1536191 | -0,7005 | -1,0136 | DOWN | DOWN | | DOWN-HIT |
| Q024552 | AKT2 | 208 | ENSG00000105221 | 3970284 | 3037367 | 0,0236 | -0,2184 | -- | -- | | -- |
| Q006903 | AKT3 | 10000 | ENSG00000117020 | 3325445 | 3098968 | -0,0653 | -0,1096 | -- | -- | | -- |
| Q024979 | AKT3 | 10000 | ENSG00000117020 | 2402976 | 2442438 | -0,3457 | -0,2425 | -- | -- | | -- |
| Q006477 | ALK | 238 | ENSG00000171094 | 2225890 | 1909403 | -0,4666 | -0,5640 | -- | -- | | -- |
| Q024553 | ALK | 238 | ENSG00000171094 | 3685006 | 2442438 | 0,0915 | -0,3501 | -- | -- | | -- |
| Q007084 | ALPK1 | 80216 | ENSG00000073331 | 2657224 | 2109793 | -0,2909 | -0,6979 | -- | -- | | -- |
| Q025160 | ALPK1 | 80216 | ENSG00000073331 | 2797936 | 2896865 | -0,1890 | -0,2875 | -- | -- | | -- |
| Q007119 | ALPK2 | 115701 | ENSG00000198796 | 2983487 | 1579971 | -0,2892 | -1,1331 | -- | DOWN | | -- |
| Q025195 | ALPK2 | 115701 | ENSG00000198796 | 3349141 | 2520693 | -0,1286 | -0,5066 | -- | -- | | -- |
| Q007040 | ALS2CR2 | 55437 | ENSG00000082146 | 3046819 | 2615019 | -0,6838 | -0,5864 | DOWN | -- | | -- |
| Q025116 | ALS2CR2 | 55437 | ENSG00000082146 | 4364738 | 3397820 | 0,0863 | -0,1059 | -- | -- | | -- |
| Q007069 | ALS2CR7 | 65061 | ENSG00000138395 | 3187880 | 2786704 | -0,2098 | -0,3733 | -- | -- | | -- |
| Q025145 | ALS2CR7 | 65061 | ENSG00000138395 | 2957222 | 2783502 | -0,2877 | -0,4436 | -- | -- | | -- |
| Q007150 | ANKK1 | 255239 | ENSG00000170209 | 3150923 | 2626392 | 0,1253 | -0,2720 | -- | -- | | -- |
| Q025226 | ANKK1 | 255239 | ENSG00000170209 | 2148343 | 2598664 | -0,3012 | -0,2754 | -- | -- | | -- |
| Q006912 | APEG1 | 10290 | ENSG00000175079 | 3071019 | 2297734 | -0,0123 | -0,5072 | -- | -- | | -- |
| Q024988 | APEG1 | 10290 | ENSG00000175079 | 2270227 | 2693868 | -0,3066 | -0,3168 | -- | -- | | -- |
| Q006478 | ARAF | 369 | ENSG00000078061 | 2630779 | 2489653 | -0,1213 | -0,2416 | -- | -- | | -- |
| Q024554 | ARAF | 369 | ENSG00000078061 | 2944528 | 3133684 | 0,0356 | 0,0607 | -- | -- | | -- |
| Q006958 | ARHGAP26 | 23092 | ENSG00000145819 | 4752076 | 2384126 | 0,0802 | -0,7700 | -- | -- | | -- |
| Q025034 | ARHGAP26 | 23092 | ENSG00000145819 | 4604756 | 3102926 | 0,1543 | -0,2781 | -- | -- | | -- |
| Q006900 | ARK5 | 9891 | ENSG00000074590 | 4019475 | 3038914 | -0,0229 | -0,2760 | -- | -- | | -- |
| Q024976 | ARK5 | 9891 | ENSG00000074590 | 4080337 | 3048401 | 0,0539 | -0,2895 | -- | -- | | -- |
| Q006930 | ASK | 10926 | ENSG00000006634 | 4180409 | 4371604 | 0,1453 | 0,2243 | -- | -- | | -- |
| Q025006 | ASK | 10926 | ENSG00000006634 | 2731224 | 2734839 | -0,4474 | -0,4837 | -- | -- | | -- |
| Q006479 | ATM | 472 | ENSG00000149311 | 2499048 | 2317027 | -0,4597 | -0,5314 | -- | -- | | -- |
| Q024555 | ATM | 472 | ENSG00000149311 | 3975091 | 3210073 | 0,1618 | -0,0254 | -- | -- | | -- |
| Q006480 | ATR | 545 | ENSG00000175054 | 3941453 | 3422400 | 0,0205 | -0,1122 | -- | -- | | -- |
| Q024556 | ATR | 545 | ENSG00000175054 | 3033586 | 1890704 | -0,4416 | -0,8418 | -- | -- | | -- |
| Q006875 | AURKB | 9212 | ENSG00000178999 | 4597739 | 2839175 | 0,0362 | -0,4726 | -- | -- | | -- |
| Q024951 | AURKB | 9212 | ENSG00000178999 | 4177009 | 2232074 | 0,0216 | -0,7404 | -- | -- | | -- |
| Q006794 | AURKC | 6795 | ENSG00000105146 | 4662249 | 3276856 | 0,0338 | -0,2789 | -- | -- | | -- |
| Q024870 | AURKC | 6795 | ENSG00000105146 | 3893977 | 3223648 | -0,1287 | -0,2312 | -- | -- | | -- |
| Q006481 | AXL | 558 | ENSG00000167601 | 4041831 | 4017917 | -0,0210 | 0,0783 | -- | -- | | -- |
| Q024557 | AXL | 558 | ENSG00000167601 | 4719254 | 3023548 | 0,1535 | -0,2688 | -- | -- | | -- |
| Q006913 | BCKDK | 10295 | ENSG00000103507 | 2522800 | 3125762 | -0,4391 | -0,1865 | -- | -- | | -- |
| Q024989 | BCKDK | 10295 | ENSG00000103507 | 2350642 | 1884209 | -0,5029 | -0,9582 | -- | DOWN | | -- |
| Q006482 | BCR | 613 | ENSG00000186716 | 4174988 | 3947163 | 0,0151 | 0,0574 | -- | -- | | -- |
| Q024558 | BCR | 613 | ENSG00000186716 | 3102097 | 2647498 | -0,5738 | -0,4879 | DOWN | -- | | -- |
| Q006483 | BLK | 640 | ENSG00000136573 | 3169932 | 1877925 | -0,3874 | -0,9695 | -- | DOWN | | -- |
| Q024559 | BLK | 640 | ENSG00000136573 | 4529820 | 3185552 | 0,1461 | -0,2142 | -- | -- | | -- |
| Q007045 | BMP2K | 55589 | ENSG00000138756 | 4296030 | 3583198 | 0,0829 | -0,0593 | -- | -- | | -- |
| Q025121 | BMP2K | 55589 | ENSG00000138756 | 3685096 | 3594813 | -0,0219 | 0,0046 | -- | -- | | -- |
| Q006484 | BMPR1A | 657 | ENSG00000107779 | 4070154 | 3568366 | 0,1132 | 0,0444 | -- | -- | | -- |
| Q024560 | BMPR1A | 657 | ENSG00000107779 | 3070454 | 2198390 | -0,3208 | -0,6363 | -- | -- | | -- |
| Q006485 | BMPR1B | 658 | ENSG00000138696 | 1767319 | 1593388 | -0,7679 | -0,7937 | DOWN | -- | | -- |
| Q024561 | BMPR1B | 658 | ENSG00000138696 | 3649892 | 3093741 | 0,1101 | -0,0234 | -- | -- | | -- |
| Q006486 | BMPR2 | 659 | NA | 2400153 | 2180474 | -0,2068 | -0,4116 | -- | -- | | -- |
| Q024562 | BMPR2 | 659 | NA | 3285728 | 2105608 | 0,1896 | -0,3577 | -- | -- | | -- |
| Q006487 | BMX | 660 | ENSG00000102010 | 4353576 | 3244471 | 0,3172 | -0,0877 | -- | -- | | -- |
| Q024563 | BMX | 660 | ENSG00000102010 | 3034009 | 2736025 | -0,1542 | -0,2106 | -- | -- | | -- |
| Q006488 | BRAF | 673 | ENSG00000157764 | 3062486 | 2341259 | -0,3290 | -0,6725 | -- | -- | | -- |
| Q024564 | BRAF | 673 | ENSG00000157764 | 3730708 | 3029566 | -0,0662 | -0,1940 | -- | -- | | -- |
| Q007099 | BRSK1 | 84446 | ENSG00000160469 | 3005543 | 1981062 | -0,0807 | -0,5421 | -- | -- | | -- |
| Q025175 | BRSK1 | 84446 | ENSG00000160469 | 3459994 | 2796261 | 0,1668 | -0,0481 | -- | -- | | -- |
| Q006489 | BTK | 695 | ENSG00000010671 | 3862488 | 2754593 | -0,0582 | -0,4978 | -- | -- | | -- |
| Q024565 | BTK | 695 | ENSG00000010671 | 3896078 | 2900438 | -0,1142 | -0,3337 | -- | -- | | -- |
| Q006490 | BUB1 | 699 | ENSG00000169679 | 4291765 | 3767202 | 0,0837 | -0,0227 | -- | -- | | -- |
| Q024566 | BUB1 | 699 | ENSG00000169679 | 4008061 | 3545637 | -0,0849 | -0,0698 | -- | -- | | -- |
| Q006491 | BUB1B | 701 | ENSG00000156970 | 3896039 | 2947195 | -0,0571 | -0,3464 | -- | -- | | -- |
| Q024567 | BUB1B | 701 | ENSG00000156970 | 4200897 | 3467524 | 0,0644 | -0,0968 | -- | -- | | -- |
| C | C | C | NA | 2151972 | 2030004 | -0,3795 | -0,3874 | -- | -- | | -- |
| C | C | C | NA | 3584097 | 2777729 | 0,2118 | -0,0590 | -- | -- | | -- |
| C | C | C | NA | 3324325 | 3807479 | -0,0261 | -0,0228 | -- | -- | | -- |
| C | C | C | NA | 3023909 | 3223709 | -0,1856 | -0,3326 | -- | -- | | -- |
| C | C | C | NA | 3327090 | 3448511 | 0,0251 | 0,1347 | -- | -- | | -- |
| C | C | C | NA | 4089331 | 4091691 | 0,2895 | 0,3380 | -- | -- | | -- |
| C | C | C | NA | 3909554 | 4001632 | 0,1480 | 0,0093 | -- | -- | | -- |
| C | C | C | NA | 3284041 | 3685984 | -0,1217 | -0,1674 | -- | -- | | -- |
| C | C | C | NA | 2469606 | 1935495 | -0,4534 | -0,6428 | -- | -- | | -- |
| C | C | C | NA | 4607179 | 3130999 | 0,3836 | -0,0642 | -- | -- | | -- |
| C | C | C | NA | 4084474 | 4404671 | 0,1747 | 0,1251 | -- | -- | | -- |
| C | C | C | NA | 3392787 | 3904255 | -0,1142 | -0,1136 | -- | -- | | -- |
| C | C | C | NA | 3936873 | 3147266 | 0,1236 | -0,0895 | -- | -- | | -- |
| C | C | C | NA | 4637803 | 3791307 | 0,3427 | 0,1418 | -- | -- | | -- |
| C | C | C | NA | 4139765 | 4268760 | 0,1671 | 0,0456 | -- | -- | | -- |
| C | C | C | NA | 3478269 | 3794504 | -0,1079 | -0,1960 | -- | -- | | -- |
| C | C | C | NA | 2466448 | 2213716 | -0,5931 | -0,5957 | DOWN | -- | | -- |
| C | C | C | NA | 4507874 | 3408249 | 0,2637 | -0,0379 | -- | -- | | -- |
| C | C | C | NA | 3589137 | 4550157 | -0,0628 | 0,1283 | -- | -- | | -- |
| C | C | C | NA | 3487326 | 2895237 | -0,1263 | -0,7121 | -- | -- | | -- |
| C | C | C | NA | 3861668 | 3437487 | 0,0371 | -0,0247 | -- | -- | | -- |
| C | C | C | NA | 4861027 | 4031998 | 0,3511 | 0,1756 | -- | -- | | -- |
| C | C | C | NA | 3888728 | 4357060 | 0,0397 | 0,0417 | -- | -- | | -- |
| C | C | C | NA | 3511111 | 3955456 | -0,1314 | -0,1686 | -- | -- | | -- |
| C | C | C | NA | 2391004 | 2075078 | -0,6914 | -0,7274 | DOWN | -- | | -- |
| C | C | C | NA | 4018862 | 3344100 | 0,0506 | -0,0988 | -- | -- | | -- |
| C | C | C | NA | 4279411 | 4150779 | 0,1730 | -0,0476 | -- | -- | | -- |
| C | C | C | NA | 3587958 | 3962538 | -0,1078 | -0,1740 | -- | -- | | -- |
| C | C | C | NA | 3708468 | 3657565 | -0,0330 | 0,0538 | -- | -- | | -- |
| C | C | C | NA | 4072067 | 4264244 | 0,0679 | 0,2460 | -- | -- | | -- |
| C | C | C | NA | 3801015 | 4372287 | -0,0081 | 0,0408 | -- | -- | | -- |
| C | C | C | NA | 3646244 | 4031993 | -0,0871 | -0,1422 | -- | -- | | -- |
| C | C | C | NA | 2202914 | 2116009 | -0,7963 | -0,6830 | DOWN | -- | | -- |
| C | C | C | NA | 4235959 | 3422290 | 0,1360 | -0,0548 | -- | -- | | -- |
| C | C | C | NA | 4246579 | 4436595 | 0,1570 | 0,0733 | -- | -- | | -- |
| C | C | C | NA | 3576434 | 3374168 | -0,1166 | -0,4486 | -- | -- | | -- |
| C | C | C | NA | 3645044 | 3647134 | -0,0226 | 0,0773 | -- | -- | | -- |
| C | C | C | NA | 4214467 | 4157348 | 0,1475 | 0,2365 | -- | -- | | -- |
| C | C | C | NA | 3949048 | 4485339 | 0,0519 | 0,1069 | -- | -- | | -- |
| C | C | C | NA | 3600872 | 3618249 | -0,1018 | -0,2993 | -- | -- | | -- |
| C | C | C | NA | 2322875 | 2285187 | -0,6199 | -0,5132 | DOWN | -- | | -- |
| C | C | C | NA | 3947674 | 3097182 | 0,0825 | -0,1399 | -- | -- | | -- |
| C | C | C | NA | 4142292 | 4402813 | 0,1333 | 0,1009 | -- | -- | | -- |
| C | C | C | NA | 3604131 | 3634164 | -0,0883 | -0,2561 | -- | -- | | -- |
| C | C | C | NA | 3319290 | 3480544 | -0,0652 | 0,0726 | -- | -- | | -- |
| C | C | C | NA | 4480251 | 3998583 | 0,3067 | 0,2371 | -- | -- | | -- |
| C | C | C | NA | 4049509 | 4306827 | 0,1197 | 0,1009 | -- | -- | | -- |
| C | C | C | NA | 3548166 | 3630607 | -0,0888 | -0,2093 | -- | -- | | -- |
| C | C | C | NA | 2295986 | 1902938 | -0,4795 | -0,6297 | -- | -- | | -- |
| C | C | C | NA | 3692449 | 2809068 | 0,0889 | -0,1836 | -- | -- | | -- |
| C | C | C | NA | 3329303 | 3427222 | 0,0607 | 0,1277 | -- | -- | | -- |
| C | C | C | NA | 4053465 | 4293834 | 0,2746 | 0,3984 | -- | -- | | -- |
| C | C | C | NA | 2641994 | 2076782 | -0,1339 | -0,4042 | -- | -- | | -- |
| C | C | C | NA | 3751649 | 3112745 | 0,2463 | 0,0423 | -- | -- | | -- |
| C | C | C | NA | 3307428 | 2976209 | 0,2175 | 0,0573 | -- | -- | | -- |
| C | C | C | NA | 3214507 | 3167973 | 0,1541 | 0,1176 | -- | -- | | -- |
| C | C | C | NA | 3485060 | 3203680 | 0,0596 | -0,1098 | -- | -- | | -- |
| C | C | C | NA | 3535180 | 3013919 | 0,1118 | -0,1412 | -- | -- | | -- |
| C | C | C | NA | 3224887 | 3644032 | -0,1181 | -0,0198 | -- | -- | | -- |
| C | C | C | NA | 3256928 | 3448711 | -0,1705 | -0,1754 | -- | -- | | -- |
| C | C | C | NA | 3274621 | 3120241 | -0,1625 | -0,2155 | -- | -- | | -- |
| C | C | C | NA | 3988806 | 3918355 | 0,1479 | 0,1540 | -- | -- | | -- |
| C | C | C | NA | 3850620 | 3918063 | 0,0602 | 0,0065 | -- | -- | | -- |
| C | C | C | NA | 3357380 | 3588848 | -0,1979 | -0,2058 | -- | -- | | -- |
| C | C | C | NA | 3712261 | 3237736 | -0,0966 | -0,2268 | -- | -- | | -- |
| C | C | C | NA | 4281959 | 3635238 | 0,1487 | -0,0044 | -- | -- | | -- |
| C | C | C | NA | 4112459 | 4009643 | 0,1069 | -0,0245 | -- | -- | | -- |
| C | C | C | NA | 3151216 | 3804203 | -0,3518 | -0,1853 | -- | -- | | -- |
| C | C | C | NA | 3680172 | 3489199 | -0,2047 | -0,1697 | -- | -- | | -- |
| C | C | C | NA | 4429102 | 3441670 | 0,1182 | -0,1349 | -- | -- | | -- |
| C | C | C | NA | 4283454 | 3767469 | 0,1326 | -0,1834 | -- | -- | | -- |
| C | C | C | NA | 3625895 | 4085109 | -0,1739 | -0,1202 | -- | -- | | -- |
| C | C | C | NA | 3524851 | 3437710 | -0,3547 | -0,2413 | -- | -- | | -- |
| C | C | C | NA | 4445022 | 3501306 | 0,0590 | -0,1529 | -- | -- | | -- |
| C | C | C | NA | 3821410 | 4343217 | -0,0665 | 0,0180 | -- | -- | | -- |
| C | C | C | NA | 3458383 | 3620011 | -0,2759 | -0,3839 | -- | -- | | -- |
| C | C | C | NA | 3896644 | 3386821 | -0,2336 | -0,3048 | -- | -- | | -- |
| C | C | C | NA | 4609476 | 3426776 | 0,0723 | -0,2215 | -- | -- | | -- |
| C | C | C | NA | 4138274 | 4290887 | 0,0410 | -0,0257 | -- | -- | | -- |
| C | C | C | NA | 3715049 | 3883483 | -0,1750 | -0,2817 | -- | -- | | -- |
| C | C | C | NA | 3565627 | 3466023 | -0,4278 | -0,2931 | -- | -- | | -- |
| C | C | C | NA | 4394036 | 3649368 | -0,0401 | -0,1451 | -- | -- | | -- |
| C | C | C | NA | 4129961 | 4367950 | 0,0319 | -0,0034 | -- | -- | | -- |
| C | C | C | NA | 3856578 | 3995040 | -0,1192 | -0,2387 | -- | -- | | -- |
| C | C | C | NA | 3700628 | 3499804 | -0,3744 | -0,2888 | -- | -- | | -- |
| C | C | C | NA | 4000792 | 4373115 | -0,2202 | 0,1325 | -- | -- | | -- |
| C | C | C | NA | 4034663 | 4216514 | -0,0028 | -0,0589 | -- | -- | | -- |
| C | C | C | NA | 3501579 | 4012054 | -0,2724 | -0,2231 | -- | -- | | -- |
| C | C | C | NA | 3642282 | 3432269 | -0,3965 | -0,3236 | -- | -- | | -- |
| C | C | C | NA | 4184998 | 3792841 | -0,1338 | -0,0954 | -- | -- | | -- |
| C | C | C | NA | 3970144 | 3798503 | -0,0197 | -0,2214 | -- | -- | | -- |
| C | C | C | NA | 3622069 | 3865882 | -0,2067 | -0,2674 | -- | -- | | -- |
| C | C | C | NA | 3706031 | 3459435 | -0,3336 | -0,3039 | -- | -- | | -- |
| C | C | C | NA | 4437282 | 4164455 | -0,0061 | 0,0587 | -- | -- | | -- |
| C | C | C | NA | 3837951 | 4274178 | -0,0571 | 0,0114 | -- | -- | | -- |
| C | C | C | NA | 3778045 | 4032328 | -0,1215 | -0,1530 | -- | -- | | -- |
| C | C | C | NA | 3685293 | 3399485 | -0,2927 | -0,3145 | -- | -- | | -- |
| C | C | C | NA | 4281990 | 3748635 | -0,0250 | -0,0921 | -- | -- | | -- |
| C | C | C | NA | 3892449 | 4366847 | -0,0140 | 0,0850 | -- | -- | | -- |
| C | C | C | NA | 3868939 | 3808977 | -0,0599 | -0,2013 | -- | -- | | -- |
| C | C | C | NA | 3481022 | 3364625 | -0,3216 | -0,2957 | -- | -- | | -- |
| C | C | C | NA | 4181224 | 4024102 | -0,0084 | 0,0482 | -- | -- | | -- |
| C | C | C | NA | 3848976 | 4101027 | -0,0027 | 0,0331 | -- | -- | | -- |
| C | C | C | NA | 3523600 | 3898045 | -0,1718 | -0,0983 | -- | -- | | -- |
| C | C | C | NA | 3549404 | 3343732 | -0,2105 | -0,2509 | -- | -- | | -- |
| C | C | C | NA | 4250422 | 3705777 | 0,0833 | -0,0331 | -- | -- | | -- |
| C | C | C | NA | 3176175 | 3248876 | -0,2905 | -0,2247 | -- | -- | | -- |
| C | C | C | NA | 4130871 | 4077206 | 0,1145 | 0,1620 | -- | -- | | -- |
| C | C | C | NA | 3035724 | 3155590 | -0,2485 | -0,1852 | -- | -- | | -- |
| C | C | C | NA | 3805786 | 3439505 | 0,0836 | -0,0122 | -- | -- | | -- |
| C | C | C | NA | 2944792 | 3338339 | -0,1691 | -0,0218 | -- | -- | | -- |
| C | C | C | NA | 2959504 | 3499042 | -0,1414 | 0,0875 | -- | -- | | -- |
| C | C | C | NA | 3236250 | 3128690 | -0,1529 | -0,3489 | -- | -- | | -- |
| C | C | C | NA | 3573328 | 3444507 | 0,0034 | -0,1571 | -- | -- | | -- |
| C | C | C | NA | 3442859 | 3662666 | -0,0298 | -0,0373 | -- | -- | | -- |
| C | C | C | NA | 3307658 | 3274348 | -0,1187 | -0,2452 | -- | -- | | -- |
| C | C | C | NA | 3959784 | 3291865 | 0,0226 | -0,3049 | -- | -- | | -- |
| C | C | C | NA | 4091520 | 4098596 | 0,0898 | 0,0760 | -- | -- | | -- |
| C | C | C | NA | 3564327 | 2917419 | -0,0589 | -0,4516 | -- | -- | | -- |
| C | C | C | NA | 3380117 | 3548509 | -0,1700 | -0,1927 | -- | -- | | -- |
| C | C | C | NA | 3625336 | 3476167 | -0,1988 | -0,2448 | -- | -- | | -- |
| C | C | C | NA | 4278028 | 3785185 | 0,0746 | -0,0686 | -- | -- | | -- |
| C | C | C | NA | 4072127 | 4012790 | 0,0700 | -0,0101 | -- | -- | | -- |
| C | C | C | NA | 3431906 | 3611902 | -0,2182 | -0,2293 | -- | -- | | -- |
| C | C | C | NA | 3750208 | 3448537 | -0,2198 | -0,2776 | -- | -- | | -- |
| C | C | C | NA | 4543019 | 3472840 | 0,1035 | -0,2213 | -- | -- | | -- |
| C | C | C | NA | 4021950 | 3947723 | 0,0041 | -0,0803 | -- | -- | | -- |
| C | C | C | NA | 3619080 | 3684161 | -0,1953 | -0,2541 | -- | -- | | -- |
| C | C | C | NA | 3758497 | 3229151 | -0,2738 | -0,4000 | -- | -- | | -- |
| C | C | C | NA | 4688452 | 3340714 | 0,1055 | -0,2953 | -- | -- | | -- |
| C | C | C | NA | 4414790 | 4110238 | 0,1092 | -0,0488 | -- | -- | | -- |
| C | C | C | NA | 3734220 | 3685315 | -0,1913 | -0,3027 | -- | -- | | -- |
| C | C | C | NA | 3858534 | 3183523 | -0,2699 | -0,4315 | -- | -- | | -- |
| C | C | C | NA | 4583880 | 3783216 | 0,0324 | -0,1022 | -- | -- | | -- |
| C | C | C | NA | 4084022 | 4173837 | -0,0386 | -0,0478 | -- | -- | | -- |
| C | C | C | NA | 3682248 | 3825452 | -0,2483 | -0,2726 | -- | -- | | -- |
| C | C | C | NA | 4031600 | 3158390 | -0,2187 | -0,4459 | -- | -- | | -- |
| C | C | C | NA | 4479248 | 4057047 | -0,0310 | 0,0082 | -- | -- | | -- |
| C | C | C | NA | 4313600 | 4245542 | 0,0290 | -0,0331 | -- | -- | | -- |
| C | C | C | NA | 3532088 | 3465996 | -0,3418 | -0,4790 | -- | -- | | -- |
| C | C | C | NA | 3874274 | 2393271 | -0,3024 | -0,9184 | -- | DOWN | | -- |
| C | C | C | NA | 4141936 | 3803765 | -0,1799 | -0,0900 | -- | -- | | -- |
| C | C | C | NA | 4083199 | 4267769 | -0,0690 | -0,0275 | -- | -- | | -- |
| C | C | C | NA | 3803242 | 3787976 | -0,2316 | -0,3197 | -- | -- | | -- |
| C | C | C | NA | 3845056 | 3532559 | -0,3125 | -0,2440 | -- | -- | | -- |
| C | C | C | NA | 4251665 | 4001231 | -0,1328 | -0,0029 | -- | -- | | -- |
| C | C | C | NA | 3996949 | 4276456 | -0,1060 | -0,0170 | -- | -- | | -- |
| C | C | C | NA | 3712700 | 3659770 | -0,2753 | -0,3740 | -- | -- | | -- |
| C | C | C | NA | 4077200 | 3250893 | -0,1864 | -0,3631 | -- | -- | | -- |
| C | C | C | NA | 4490367 | 4096796 | -0,0215 | 0,0439 | -- | -- | | -- |
| C | C | C | NA | 4262653 | 4339807 | 0,0032 | 0,0240 | -- | -- | | -- |
| C | C | C | NA | 3772610 | 3946733 | -0,2416 | -0,2140 | -- | -- | | -- |
| C | C | C | NA | 3724921 | 3295256 | -0,3027 | -0,3191 | -- | -- | | -- |
| C | C | C | NA | 4562496 | 3829488 | 0,0359 | -0,0470 | -- | -- | | -- |
| C | C | C | NA | 4322315 | 4159620 | 0,0399 | -0,0206 | -- | -- | | -- |
| C | C | C | NA | 3635601 | 3857590 | -0,2856 | -0,2211 | -- | -- | | -- |
| C | C | C | NA | 3658657 | 3310001 | -0,2762 | -0,2878 | -- | -- | | -- |
| C | C | C | NA | 4689265 | 3900250 | 0,1232 | -0,0006 | -- | -- | | -- |
| C | C | C | NA | 4216489 | 4133834 | 0,0249 | 0,0051 | -- | -- | | -- |
| C | C | C | NA | 3809540 | 3904333 | -0,1807 | -0,1557 | -- | -- | | -- |
| C | C | C | NA | 3893075 | 3440090 | -0,1113 | -0,2001 | -- | -- | | -- |
| C | C | C | NA | 4581512 | 3812126 | 0,1383 | -0,0128 | -- | -- | | -- |
| C | C | C | NA | 3608873 | 3173929 | -0,1535 | -0,2894 | -- | -- | | -- |
| C | C | C | NA | 4058720 | 3859783 | 0,0188 | 0,0328 | -- | -- | | -- |
| C | C | C | NA | 3308119 | 3612443 | -0,1860 | -0,0574 | -- | -- | | -- |
| C | C | C | NA | 3912532 | 3733217 | 0,0489 | 0,0164 | -- | -- | | -- |
| C | C | C | NA | 3030560 | 3198672 | -0,1858 | -0,1909 | -- | -- | | -- |
| C | C | C | NA | 3135395 | 3279907 | -0,1433 | -0,1274 | -- | -- | | -- |
| C | C | C | NA | 3244969 | 3000890 | -0,0729 | -0,2071 | -- | -- | | -- |
| C | C | C | NA | 3141038 | 3277804 | -0,0868 | -0,0664 | -- | -- | | -- |
| C | C | C | NA | 3174956 | 3709615 | -0,0651 | -0,0375 | -- | -- | | -- |
| C | C | C | NA | 2986574 | 3142561 | -0,1759 | -0,3370 | -- | -- | | -- |
| C | C | C | NA | 3497105 | 3027223 | -0,0587 | -0,2477 | -- | -- | | -- |
| C | C | C | NA | 4007893 | 3870031 | 0,1573 | 0,1209 | -- | -- | | -- |
| C | C | C | NA | 3697256 | 3649140 | 0,0687 | -0,0898 | -- | -- | | -- |
| C | C | C | NA | 3390766 | 3487867 | -0,0769 | -0,2025 | -- | -- | | -- |
| C | C | C | NA | 3500348 | 3259964 | -0,1334 | -0,1880 | -- | -- | | -- |
| C | C | C | NA | 4303934 | 3372056 | 0,1944 | -0,1128 | -- | -- | | -- |
| C | C | C | NA | 4196556 | 4132193 | 0,1951 | 0,0775 | -- | -- | | -- |
| C | C | C | NA | 3373431 | 3539875 | -0,1405 | -0,2081 | -- | -- | | -- |
| C | C | C | NA | 3893603 | 3321107 | -0,0341 | -0,2015 | -- | -- | | -- |
| C | C | C | NA | 4330604 | 3793255 | 0,1499 | 0,0280 | -- | -- | | -- |
| C | C | C | NA | 4205495 | 4296825 | 0,1604 | 0,1172 | -- | -- | | -- |
| C | C | C | NA | 3699135 | 3718920 | -0,0483 | -0,1548 | -- | -- | | -- |
| C | C | C | NA | 3781840 | 3525408 | -0,1297 | -0,1444 | -- | -- | | -- |
| C | C | C | NA | 4316548 | 3756964 | 0,1003 | -0,0101 | -- | -- | | -- |
| C | C | C | NA | 4063954 | 3787968 | 0,0812 | -0,1027 | -- | -- | | -- |
| C | C | C | NA | 3847499 | 3468448 | -0,0188 | -0,2955 | -- | -- | | -- |
| C | C | C | NA | 3857497 | 3618412 | -0,1369 | -0,1301 | -- | -- | | -- |
| C | C | C | NA | 4519182 | 3958998 | 0,1384 | 0,0515 | -- | -- | | -- |
| C | C | C | NA | 4011181 | 3845841 | 0,0413 | -0,0937 | -- | -- | | -- |
| C | C | C | NA | 3741416 | 3524668 | -0,0811 | -0,2882 | -- | -- | | -- |
| C | C | C | NA | 3809047 | 3296161 | -0,1861 | -0,2976 | -- | -- | | -- |
| C | C | C | NA | 4293654 | 4004845 | 0,0314 | 0,0578 | -- | -- | | -- |
| C | C | C | NA | 3940035 | 3922226 | 0,0023 | -0,0709 | -- | -- | | -- |
| C | C | C | NA | 3700739 | 3478457 | -0,1091 | -0,3233 | -- | -- | | -- |
| C | C | C | NA | 3706804 | 3463569 | -0,2498 | -0,2283 | -- | -- | | -- |
| C | C | C | NA | 4297838 | 4278826 | 0,0180 | 0,1533 | -- | -- | | -- |
| C | C | C | NA | 3667525 | 4069127 | -0,1115 | -0,0125 | -- | -- | | -- |
| C | C | C | NA | 3538448 | 3695121 | -0,1825 | -0,2221 | -- | -- | | -- |
| C | C | C | NA | 3591974 | 3136332 | -0,3077 | -0,3901 | -- | -- | | -- |
| C | C | C | NA | 3941782 | 3774723 | -0,1288 | -0,0379 | -- | -- | | -- |
| C | C | C | NA | 3805721 | 4206268 | -0,0505 | 0,0478 | -- | -- | | -- |
| C | C | C | NA | 3568847 | 3744415 | -0,1646 | -0,1919 | -- | -- | | -- |
| C | C | C | NA | 3798948 | 3264360 | -0,1993 | -0,3179 | -- | -- | | -- |
| C | C | C | NA | 4211682 | 3883747 | -0,0083 | 0,0134 | -- | -- | | -- |
| C | C | C | NA | 3980629 | 4196276 | 0,0308 | 0,0588 | -- | -- | | -- |
| C | C | C | NA | 3403383 | 3821483 | -0,2235 | -0,1398 | -- | -- | | -- |
| C | C | C | NA | 3535178 | 3261277 | -0,2920 | -0,3049 | -- | -- | | -- |
| C | C | C | NA | 4295383 | 3674247 | 0,0468 | -0,0572 | -- | -- | | -- |
| C | C | C | NA | 3991467 | 4304975 | 0,0560 | 0,1225 | -- | -- | | -- |
| C | C | C | NA | 3622166 | 3710271 | -0,1048 | -0,1605 | -- | -- | | -- |
| C | C | C | NA | 3410301 | 3340173 | -0,3066 | -0,2462 | -- | -- | | -- |
| C | C | C | NA | 4394734 | 4068347 | 0,1175 | 0,1140 | -- | -- | | -- |
| C | C | C | NA | 3737544 | 3960036 | -0,0108 | 0,0257 | -- | -- | | -- |
| C | C | C | NA | 3524751 | 3623997 | -0,1134 | -0,1588 | -- | -- | | -- |
| C | C | C | NA | 3701155 | 3360120 | -0,1186 | -0,2049 | -- | -- | | -- |
| C | C | C | NA | 3925814 | 3968272 | -0,0089 | 0,1026 | -- | -- | | -- |
| C | C | C | NA | 3571956 | 3199471 | -0,1029 | -0,2321 | -- | -- | | -- |
| C | C | C | NA | 3789546 | 4112664 | 0,0048 | 0,1901 | -- | -- | | -- |
| C | C | C | NA | 3310566 | 3167393 | -0,1247 | -0,1895 | -- | -- | | -- |
| C | C | C | NA | 3588782 | 3837320 | 0,0099 | 0,1360 | -- | -- | | -- |
| C | C | C | NA | 3225125 | 3231568 | -0,0543 | -0,1005 | -- | -- | | -- |
| C | C | C | NA | 3189936 | 3342278 | -0,0454 | 0,0012 | -- | -- | | -- |
| C | C | C | NA | 3276806 | 3061996 | 0,0542 | -0,1738 | -- | -- | | -- |
| C | C | C | NA | 3307077 | 3612562 | 0,0748 | 0,0873 | -- | -- | | -- |
| C | C | C | NA | 3022477 | 3923342 | 0,0439 | 0,1498 | -- | -- | | -- |
| C | C | C | NA | 3287886 | 3835856 | 0,1136 | 0,0900 | -- | -- | | -- |
| C | C | C | NA | 3137452 | 3820789 | 0,0192 | 0,0608 | -- | -- | | -- |
| C | C | C | NA | 3421390 | 3810606 | 0,0855 | 0,0484 | -- | -- | | -- |
| C | C | C | NA | 3039935 | 3154975 | -0,0783 | -0,2232 | -- | -- | | -- |
| C | C | C | NA | 3466793 | 3314236 | 0,0109 | -0,1152 | -- | -- | | -- |
| C | C | C | NA | 3699878 | 3854739 | 0,1043 | 0,1316 | -- | -- | | -- |
| C | C | C | NA | 3448354 | 4482931 | 0,1058 | 0,2999 | -- | -- | | -- |
| C | C | C | NA | 3684370 | 4577504 | 0,1718 | 0,2999 | -- | -- | | -- |
| C | C | C | NA | 3542041 | 4398034 | 0,0982 | 0,2201 | -- | -- | | -- |
| C | C | C | NA | 3652638 | 4087689 | 0,1136 | 0,0979 | -- | -- | | -- |
| C | C | C | NA | 3653060 | 3769795 | 0,1022 | -0,0243 | -- | -- | | -- |
| C | C | C | NA | 3211968 | 3478573 | -0,0788 | -0,1378 | -- | -- | | -- |
| C | C | C | NA | 3478991 | 3266442 | -0,0805 | -0,1780 | -- | -- | | -- |
| C | C | C | NA | 4103708 | 3493216 | 0,1574 | -0,0457 | -- | -- | | -- |
| C | C | C | NA | 4046309 | 4655477 | 0,2253 | 0,2866 | -- | -- | | -- |
| C | C | C | NA | 4027993 | 4529418 | 0,2041 | 0,2209 | -- | -- | | -- |
| C | C | C | NA | 3845462 | 4175197 | 0,1286 | 0,0817 | -- | -- | | -- |
| C | C | C | NA | 3837575 | 3611644 | 0,1160 | -0,1402 | -- | -- | | -- |
| C | C | C | NA | 3680337 | 3594453 | 0,0540 | -0,1317 | -- | -- | | -- |
| C | C | C | NA | 3847024 | 3300447 | -0,0087 | -0,1942 | -- | -- | | -- |
| C | C | C | NA | 4503032 | 3962692 | 0,2248 | 0,1107 | -- | -- | | -- |
| C | C | C | NA | 4256977 | 4616029 | 0,2366 | 0,2492 | -- | -- | | -- |
| C | C | C | NA | 4288266 | 4788681 | 0,2384 | 0,2703 | -- | -- | | -- |
| C | C | C | NA | 4142889 | 4519858 | 0,1823 | 0,1648 | -- | -- | | -- |
| C | C | C | NA | 3990599 | 4304823 | 0,1290 | 0,0915 | -- | -- | | -- |
| C | C | C | NA | 4119605 | 3947741 | 0,1758 | -0,0355 | -- | -- | | -- |
| C | C | C | NA | 3848755 | 3649419 | 0,0828 | -0,1393 | -- | -- | | -- |
| C | C | C | NA | 3707790 | 3402771 | -0,1221 | -0,1692 | -- | -- | | -- |
| C | C | C | NA | 4184050 | 4070392 | 0,0593 | 0,1333 | -- | -- | | -- |
| C | C | C | NA | 4083168 | 4777482 | 0,1242 | 0,2428 | -- | -- | | -- |
| C | C | C | NA | 4126246 | 4552493 | 0,1339 | 0,1408 | -- | -- | | -- |
| C | C | C | NA | 3996796 | 4620121 | 0,0911 | 0,1666 | -- | -- | | -- |
| C | C | C | NA | 3735304 | 4133185 | 0,0070 | 0,0130 | -- | -- | | -- |
| C | C | C | NA | 3535978 | 3630283 | -0,0604 | -0,1687 | -- | -- | | -- |
| C | C | C | NA | 3684403 | 3544642 | -0,1751 | -0,1181 | -- | -- | | -- |
| C | C | C | NA | 4638006 | 4048030 | 0,1817 | 0,1159 | -- | -- | | -- |
| C | C | C | NA | 4319029 | 4415932 | 0,1751 | 0,1147 | -- | -- | | -- |
| C | C | C | NA | 4124741 | 4556440 | 0,0993 | 0,1178 | -- | -- | | -- |
| C | C | C | NA | 3938235 | 4491182 | 0,0343 | 0,0873 | -- | -- | | -- |
| C | C | C | NA | 3882397 | 4463716 | 0,0289 | 0,0995 | -- | -- | | -- |
| C | C | C | NA | 4054591 | 4036540 | 0,1056 | -0,0429 | -- | -- | | -- |
| C | C | C | NA | 3696279 | 3511439 | -0,0113 | -0,2349 | -- | -- | | -- |
| C | C | C | NA | 4043867 | 3572497 | -0,0524 | -0,1091 | -- | -- | | -- |
| C | C | C | NA | 4333233 | 3996941 | 0,0465 | 0,0948 | -- | -- | | -- |
| C | C | C | NA | 3925427 | 4755430 | 0,0191 | 0,2594 | -- | -- | | -- |
| C | C | C | NA | 4157538 | 4823168 | 0,0894 | 0,2121 | -- | -- | | -- |
| C | C | C | NA | 4332216 | 4641588 | 0,1526 | 0,1244 | -- | -- | | -- |
| C | C | C | NA | 4119634 | 4597458 | 0,0893 | 0,1197 | -- | -- | | -- |
| C | C | C | NA | 3914753 | 4197755 | 0,0419 | 0,0145 | -- | -- | | -- |
| C | C | C | NA | 3507843 | 3584376 | -0,0951 | -0,2041 | -- | -- | | -- |
| C | C | C | NA | 3897766 | 3385926 | -0,1290 | -0,1873 | -- | -- | | -- |
| C | C | C | NA | 4493081 | 4307476 | 0,0902 | 0,2107 | -- | -- | | -- |
| C | C | C | NA | 3874168 | 5069143 | -0,0205 | 0,3218 | -- | -- | | -- |
| C | C | C | NA | 4103915 | 4822305 | 0,0565 | 0,1896 | -- | -- | | -- |
| C | C | C | NA | 3981820 | 4570233 | 0,0200 | 0,0961 | -- | -- | | -- |
| C | C | C | NA | 4156320 | 4403064 | 0,1068 | 0,0645 | -- | -- | | -- |
| C | C | C | NA | 4117011 | 4182428 | 0,1101 | 0,0093 | -- | -- | | -- |
| C | C | C | NA | 3875368 | 3546377 | 0,0452 | -0,2183 | -- | -- | | -- |
| C | C | C | NA | 3772526 | 3069377 | -0,1873 | -0,3265 | -- | -- | | -- |
| C | C | C | NA | 4070164 | 3664007 | -0,0736 | -0,0194 | -- | -- | | -- |
| C | C | C | NA | 4031825 | 4877336 | 0,0539 | 0,3073 | -- | -- | | -- |
| C | C | C | NA | 4464489 | 4858170 | 0,1869 | 0,2286 | -- | -- | | -- |
| C | C | C | NA | 4593431 | 4579843 | 0,2308 | 0,1065 | -- | -- | | -- |
| C | C | C | NA | 4162387 | 4414500 | 0,0959 | 0,0580 | -- | -- | | -- |
| C | C | C | NA | 3881599 | 4124807 | 0,0231 | -0,0042 | -- | -- | | -- |
| C | C | C | NA | 3615376 | 3780233 | -0,0562 | -0,1038 | -- | -- | | -- |
| C | C | C | NA | 3673102 | 3351563 | -0,2189 | -0,1732 | -- | -- | | -- |
| C | C | C | NA | 4307717 | 3759826 | 0,0356 | 0,0373 | -- | -- | | -- |
| C | C | C | NA | 4532842 | 4796083 | 0,2286 | 0,2579 | -- | -- | | -- |
| C | C | C | NA | 4149038 | 5029122 | 0,0834 | 0,2886 | -- | -- | | -- |
| C | C | C | NA | 4029875 | 4601355 | 0,0470 | 0,1385 | -- | -- | | -- |
| C | C | C | NA | 4014425 | 4402535 | 0,0631 | 0,0932 | -- | -- | | -- |
| C | C | C | NA | 4167277 | 4175228 | 0,1353 | 0,0344 | -- | -- | | -- |
| C | C | C | NA | 3471194 | 3691200 | -0,1083 | -0,1241 | -- | -- | | -- |
| C | C | C | NA | 3741859 | 2965194 | -0,1593 | -0,3296 | -- | -- | | -- |
| C | C | C | NA | 4382788 | 3586347 | 0,0925 | -0,0060 | -- | -- | | -- |
| C | C | C | NA | 4383131 | 4773579 | 0,2099 | 0,3126 | -- | -- | | -- |
| C | C | C | NA | 4483607 | 4436976 | 0,2246 | 0,1320 | -- | -- | | -- |
| C | C | C | NA | 4204850 | 4751132 | 0,1259 | 0,2222 | -- | -- | | -- |
| C | C | C | NA | 3792355 | 4627559 | -0,0155 | 0,1881 | -- | -- | | -- |
| C | C | C | NA | 3834493 | 4000212 | 0,0296 | -0,0059 | -- | -- | | -- |
| C | C | C | NA | 3416593 | 3680034 | -0,1149 | -0,1003 | -- | -- | | -- |
| C | C | C | NA | 3674738 | 3265711 | -0,1434 | -0,1509 | -- | -- | | -- |
| C | C | C | NA | 4334585 | 3817522 | 0,1146 | 0,1143 | -- | -- | | -- |
| C | C | C | NA | 4152178 | 4267267 | 0,1542 | 0,1425 | -- | -- | | -- |
| C | C | C | NA | 4075330 | 4249953 | 0,1120 | 0,0924 | -- | -- | | -- |
| C | C | C | NA | 3622282 | 4264164 | -0,0584 | 0,0924 | -- | -- | | -- |
| C | C | C | NA | 3846900 | 4396224 | 0,0462 | 0,1606 | -- | -- | | -- |
| C | C | C | NA | 4064149 | 3746955 | 0,1389 | -0,0678 | -- | -- | | -- |
| C | C | C | NA | 3628322 | 3667655 | -0,0040 | -0,0658 | -- | -- | | -- |
| C | C | C | NA | 3503008 | 3299542 | -0,1610 | -0,0917 | -- | -- | | -- |
| C | C | C | NA | 4000524 | 3784675 | 0,0428 | 0,1393 | -- | -- | | -- |
| C | C | C | NA | 4306316 | 4572769 | 0,2606 | 0,3261 | -- | -- | | -- |
| C | C | C | NA | 4418132 | 4052615 | 0,2775 | 0,0918 | -- | -- | | -- |
| C | C | C | NA | 3727975 | 4378697 | 0,0279 | 0,1907 | -- | -- | | -- |
| C | C | C | NA | 3820662 | 3821791 | 0,0674 | -0,0154 | -- | -- | | -- |
| C | C | C | NA | 3741320 | 3230672 | 0,0006 | -0,0669 | -- | -- | | -- |
| C | C | C | NA | 3913857 | 3826837 | 0,0750 | 0,1987 | -- | -- | | -- |
| C | C | C | NA | 4166579 | 4033658 | 0,2755 | 0,2317 | -- | -- | | -- |
| C | C | C | NA | 3995647 | 4027921 | 0,2024 | 0,1731 | -- | -- | | -- |
| C | C | C | NA | 3818143 | 4035075 | 0,1241 | 0,1400 | -- | -- | | -- |
| C | C | C | NA | 4152417 | 3993846 | 0,2321 | 0,1176 | -- | -- | | -- |
| C | C | C | NA | 3291488 | 4062707 | -0,0767 | 0,1545 | -- | -- | | -- |
| C | C | C | NA | 3027128 | 2739431 | -0,2143 | -0,2171 | -- | -- | | -- |
| C | C | C | NA | 3716899 | 3310782 | 0,0818 | 0,0646 | -- | -- | | -- |
| C | C | C | NA | 3984130 | 3756119 | 0,2744 | 0,1760 | -- | -- | | -- |
| C | C | C | NA | 4068811 | 3865410 | 0,2841 | 0,1688 | -- | -- | | -- |
| C | C | C | NA | 4042825 | 3676489 | 0,2622 | 0,0818 | -- | -- | | -- |
| C | C | C | NA | 3701449 | 3810428 | 0,1413 | 0,1312 | -- | -- | | -- |
| C | C | C | NA | 2939428 | 3118616 | -0,1407 | 0,0192 | -- | -- | | -- |
| C | C | C | NA | 3339709 | 3156409 | 0,0399 | 0,0715 | -- | -- | | -- |
| C | C | C | NA | 3541874 | 3208740 | 0,2227 | 0,0858 | -- | -- | | -- |
| C | C | C | NA | 3467594 | 3166477 | 0,1769 | 0,0179 | -- | -- | | -- |
| C | C | C | NA | 3352689 | 3583385 | 0,1162 | 0,1432 | -- | -- | | -- |
| C | C | C | NA | 3406874 | 3495422 | 0,1199 | 0,1034 | -- | -- | | -- |
| C | C | C | NA | 3139866 | 3359830 | 0,0131 | 0,0623 | -- | -- | | -- |
| C | C | C | NA | 3264744 | 3159809 | 0,0671 | -0,1578 | -- | -- | | -- |
| C | C | C | NA | 3541291 | 3255383 | 0,1706 | -0,0729 | -- | -- | | -- |
| C | C | C | NA | 3269596 | 3916693 | 0,1231 | 0,1576 | -- | -- | | -- |
| C | C | C | NA | 3137895 | 4122352 | 0,0473 | 0,2021 | -- | -- | | -- |
| C | C | C | NA | 3385446 | 3649321 | 0,0955 | -0,0001 | -- | -- | | -- |
| C | C | C | NA | 3232650 | 3579103 | -0,0122 | -0,0452 | -- | -- | | -- |
| C | C | C | NA | 3304496 | 2750803 | -0,0097 | -0,4417 | -- | -- | | -- |
| C | C | C | NA | 3372858 | 3302952 | -0,0173 | -0,1529 | -- | -- | | -- |
| C | C | C | NA | 3773990 | 3989414 | 0,1343 | 0,1625 | -- | -- | | -- |
| C | C | C | NA | 3731848 | 4470223 | 0,1847 | 0,3163 | -- | -- | | -- |
| C | C | C | NA | 3821679 | 4341960 | 0,2007 | 0,2398 | -- | -- | | -- |
| C | C | C | NA | 3678841 | 4424400 | 0,1267 | 0,2439 | -- | -- | | -- |
| C | C | C | NA | 3783297 | 3681995 | 0,1333 | -0,0562 | -- | -- | | -- |
| C | C | C | NA | 3608224 | 3689048 | 0,0524 | -0,0586 | -- | -- | | -- |
| C | C | C | NA | 3414131 | 3546985 | -0,0426 | -0,1199 | -- | -- | | -- |
| C | C | C | NA | 3454847 | 3397345 | -0,0869 | -0,1573 | -- | -- | | -- |
| C | C | C | NA | 4053770 | 3750346 | 0,1405 | 0,0348 | -- | -- | | -- |
| C | C | C | NA | 3769485 | 4579057 | 0,1057 | 0,2868 | -- | -- | | -- |
| C | C | C | NA | 3511335 | 4186462 | -0,0069 | 0,1208 | -- | -- | | -- |
| C | C | C | NA | 4136911 | 4125861 | 0,2018 | 0,0761 | -- | -- | | -- |
| C | C | C | NA | 3755413 | 3818221 | 0,0476 | -0,0560 | -- | -- | | -- |
| C | C | C | NA | 3384760 | 3264985 | -0,1129 | -0,3044 | -- | -- | | -- |
| C | C | C | NA | 3626281 | 3395617 | -0,0949 | -0,1937 | -- | -- | | -- |
| C | C | C | NA | 4256292 | 4211237 | 0,1426 | 0,1806 | -- | -- | | -- |
| C | C | C | NA | 4279742 | 4591537 | 0,2112 | 0,2694 | -- | -- | | -- |
| C | C | C | NA | 3937579 | 4585521 | 0,0908 | 0,2289 | -- | -- | | -- |
| C | C | C | NA | 4265745 | 4583349 | 0,1958 | 0,2061 | -- | -- | | -- |
| C | C | C | NA | 4106408 | 4444117 | 0,1352 | 0,1464 | -- | -- | | -- |
| C | C | C | NA | 3890087 | 4356170 | 0,0530 | 0,1162 | -- | -- | | -- |
| C | C | C | NA | 3732818 | 3507051 | -0,0134 | -0,2299 | -- | -- | | -- |
| C | C | C | NA | 3747936 | 3516221 | -0,1007 | -0,1630 | -- | -- | | -- |
| C | C | C | NA | 4143555 | 4335647 | 0,0514 | 0,2084 | -- | -- | | -- |
| C | C | C | NA | 3849444 | 4669400 | 0,0054 | 0,2361 | -- | -- | | -- |
| C | C | C | NA | 3940674 | 4531449 | 0,0358 | 0,1576 | -- | -- | | -- |
| C | C | C | NA | 4030207 | 4548531 | 0,0695 | 0,1504 | -- | -- | | -- |
| C | C | C | NA | 3993395 | 3967377 | 0,0594 | -0,0695 | -- | -- | | -- |
| C | C | C | NA | 3578697 | 3384071 | -0,1021 | -0,3215 | -- | -- | | -- |
| C | C | C | NA | 3740069 | 3304212 | -0,1418 | -0,2767 | -- | -- | | -- |
| C | C | C | NA | 4346688 | 4132081 | 0,0918 | 0,1272 | -- | -- | | -- |
| C | C | C | NA | 4453972 | 5083240 | 0,1807 | 0,3692 | -- | -- | | -- |
| C | C | C | NA | 4169875 | 4920474 | 0,0864 | 0,2737 | -- | -- | | -- |
| C | C | C | NA | 3804542 | 4644641 | -0,0512 | 0,1596 | -- | -- | | -- |
| C | C | C | NA | 4081721 | 4425449 | 0,0645 | 0,0804 | -- | -- | | -- |
| C | C | C | NA | 3930770 | 4070941 | 0,0142 | -0,0490 | -- | -- | | -- |
| C | C | C | NA | 3694094 | 3615617 | -0,0718 | -0,2285 | -- | -- | | -- |
| C | C | C | NA | 3724709 | 3538697 | -0,1720 | -0,1702 | -- | -- | | -- |
| C | C | C | NA | 4159338 | 3995183 | 0,0014 | 0,0748 | -- | -- | | -- |
| C | C | C | NA | 4710651 | 4999750 | 0,2408 | 0,3602 | -- | -- | | -- |
| C | C | C | NA | 4605143 | 4487734 | 0,2153 | 0,1218 | -- | -- | | -- |
| C | C | C | NA | 4228098 | 4844850 | 0,0873 | 0,2180 | -- | -- | | -- |
| C | C | C | NA | 3986167 | 4597463 | 0,0054 | 0,1237 | -- | -- | | -- |
| C | C | C | NA | 3989344 | 4130811 | 0,0231 | -0,0360 | -- | -- | | -- |
| C | C | C | NA | 3873318 | 3545259 | -0,0101 | -0,2711 | -- | -- | | -- |
| C | C | C | NA | 3898323 | 3636191 | -0,1105 | -0,1215 | -- | -- | | -- |
| C | C | C | NA | 4432374 | 4134141 | 0,0934 | 0,1326 | -- | -- | | -- |
| C | C | C | NA | 3868298 | 4995206 | -0,0633 | 0,3235 | -- | -- | | -- |
| C | C | C | NA | 4537297 | 4379624 | 0,1873 | 0,0528 | -- | -- | | -- |
| C | C | C | NA | 4308013 | 4804714 | 0,1121 | 0,1936 | -- | -- | | -- |
| C | C | C | NA | 3826698 | 4418091 | -0,0574 | 0,0610 | -- | -- | | -- |
| C | C | C | NA | 3864931 | 4124327 | -0,0320 | -0,0390 | -- | -- | | -- |
| C | C | C | NA | 3539669 | 3744489 | -0,1520 | -0,1755 | -- | -- | | -- |
| C | C | C | NA | 3924497 | 3344265 | -0,0965 | -0,2405 | -- | -- | | -- |
| C | C | C | NA | 4225162 | 3814463 | 0,0236 | 0,0238 | -- | -- | | -- |
| C | C | C | NA | 4659026 | 4272534 | 0,2383 | 0,1231 | -- | -- | | -- |
| C | C | C | NA | 4289637 | 4831855 | 0,1077 | 0,2484 | -- | -- | | -- |
| C | C | C | NA | 4304889 | 5087754 | 0,1132 | 0,3036 | -- | -- | | -- |
| C | C | C | NA | 4303006 | 4547089 | 0,1182 | 0,1119 | -- | -- | | -- |
| C | C | C | NA | 3953799 | 4140029 | 0,0037 | -0,0207 | -- | -- | | -- |
| C | C | C | NA | 3723487 | 3570922 | -0,0750 | -0,2432 | -- | -- | | -- |
| C | C | C | NA | 3496459 | 3207544 | -0,2614 | -0,2826 | -- | -- | | -- |
| C | C | C | NA | 4296323 | 4337233 | 0,0756 | 0,2365 | -- | -- | | -- |
| C | C | C | NA | 4296878 | 4816360 | 0,1403 | 0,2960 | -- | -- | | -- |
| C | C | C | NA | 4310677 | 4469735 | 0,1331 | 0,1219 | -- | -- | | -- |
| C | C | C | NA | 4030948 | 4724230 | 0,0305 | 0,1983 | -- | -- | | -- |
| C | C | C | NA | 3946883 | 4614408 | 0,0051 | 0,1679 | -- | -- | | -- |
| C | C | C | NA | 3854255 | 4067597 | -0,0234 | -0,0261 | -- | -- | | -- |
| C | C | C | NA | 3599726 | 3735693 | -0,1153 | -0,1410 | -- | -- | | -- |
| C | C | C | NA | 3606839 | 3382110 | -0,1783 | -0,1709 | -- | -- | | -- |
| C | C | C | NA | 4088077 | 3662113 | 0,0327 | 0,0116 | -- | -- | | -- |
| C | C | C | NA | 4698184 | 4578876 | 0,3232 | 0,2859 | -- | -- | | -- |
| C | C | C | NA | 4450573 | 4416572 | 0,2168 | 0,1601 | -- | -- | | -- |
| C | C | C | NA | 4079077 | 4103910 | 0,0764 | 0,0096 | -- | -- | | -- |
| C | C | C | NA | 3796310 | 4385345 | -0,0329 | 0,1126 | -- | -- | | -- |
| C | C | C | NA | 4067605 | 3807748 | 0,0778 | -0,0972 | -- | -- | | -- |
| C | C | C | NA | 3727150 | 3397973 | -0,0433 | -0,2567 | -- | -- | | -- |
| C | C | C | NA | 3764018 | 3147739 | -0,0774 | -0,2478 | -- | -- | | -- |
| C | C | C | NA | 4274200 | 3829084 | 0,1336 | 0,1043 | -- | -- | | -- |
| C | C | C | NA | 4320764 | 4366576 | 0,2285 | 0,2184 | -- | -- | | -- |
| C | C | C | NA | 3856601 | 4463345 | 0,0377 | 0,2053 | -- | -- | | -- |
| C | C | C | NA | 3929071 | 4497111 | 0,0539 | 0,2024 | -- | -- | | -- |
| C | C | C | NA | 3634799 | 4353403 | -0,0619 | 0,1546 | -- | -- | | -- |
| C | C | C | NA | 3743377 | 4180543 | -0,0156 | 0,1029 | -- | -- | | -- |
| C | C | C | NA | 3717306 | 3520932 | -0,0192 | -0,1437 | -- | -- | | -- |
| C | C | C | NA | 3547318 | 3325871 | -0,1346 | -0,1299 | -- | -- | | -- |
| C | C | C | NA | 4150226 | 3661999 | 0,1215 | 0,0707 | -- | -- | | -- |
| C | C | C | NA | 4058034 | 4531503 | 0,2044 | 0,3542 | -- | -- | | -- |
| C | C | C | NA | 4296287 | 4200406 | 0,2489 | 0,1872 | -- | -- | | -- |
| C | C | C | NA | 4108429 | 4118505 | 0,1658 | 0,1328 | -- | -- | | -- |
| C | C | C | NA | 4093247 | 4501098 | 0,1496 | 0,2597 | -- | -- | | -- |
| C | C | C | NA | 3393807 | 3359575 | -0,1654 | -0,0765 | -- | -- | | -- |
| C | C | C | NA | 4063994 | 3724268 | 0,1278 | 0,1277 | -- | -- | | -- |
| C | C | C | NA | 4155521 | 4096608 | 0,2967 | 0,2963 | -- | -- | | -- |
| C | C | C | NA | 4115844 | 4381816 | 0,2542 | 0,3346 | -- | -- | | -- |
| C | C | C | NA | 4279241 | 4126660 | 0,2783 | 0,2137 | -- | -- | | -- |
| C | C | C | NA | 4103799 | 3838292 | 0,2025 | 0,0960 | -- | -- | | -- |
| C | C | C | NA | 3666937 | 3897665 | 0,0370 | 0,1193 | -- | -- | | -- |
| C | C | C | NA | 3639215 | 3208707 | -0,0260 | -0,1001 | -- | -- | | -- |
| C | C | C | NA | 3931269 | 2995084 | 0,1218 | -0,1275 | -- | -- | | -- |
| C | C | C | NA | 4011213 | 3443958 | 0,2849 | 0,1054 | -- | -- | | -- |
| C | C | C | NA | 4093427 | 3737547 | 0,2824 | 0,1662 | -- | -- | | -- |
| C | C | C | NA | 3844087 | 3696789 | 0,1742 | 0,1319 | -- | -- | | -- |
| C | C | C | NA | 4041835 | 3344087 | 0,2222 | -0,0103 | -- | -- | | -- |
| C | C | C | NA | 3007653 | 3128676 | -0,2455 | -0,0925 | -- | -- | | -- |
| C | C | C | NA | 3247217 | 3417702 | -0,0914 | 0,0859 | -- | -- | | -- |
| C | C | C | NA | 3524619 | 3527398 | 0,1906 | 0,2317 | -- | -- | | -- |
| C | C | C | NA | 3677919 | 3386740 | 0,2151 | 0,1422 | -- | -- | | -- |
| C | C | C | NA | 3493404 | 3440393 | 0,1214 | 0,1332 | -- | -- | | -- |
| C | C | C | NA | 3345713 | 3603830 | 0,0404 | 0,1845 | -- | -- | | -- |
| C | C | C | NA | 3215276 | 3315929 | -0,0284 | 0,0875 | -- | -- | | -- |
| Q007104 | C21orf124 | 85006 | ENSG00000160209 | 3479141 | 2910330 | -0,1988 | -0,4396 | -- | -- | | -- |
| Q025180 | C21orf124 | 85006 | ENSG00000160209 | 4225660 | 3210435 | 0,1474 | -0,2626 | -- | -- | | -- |
| Q006493 | CALM1 | 801 | ENSG00000143933 | 4514152 | 3581148 | 0,4131 | 0,1497 | -- | -- | | -- |
| Q024569 | CALM1 | 801 | ENSG00000143933 | 3132537 | 2085541 | -0,0589 | -0,4831 | -- | -- | | -- |
| Q006494 | CALM2 | 805 | ENSG00000143933 | 2147444 | 2501262 | -0,3146 | -0,2711 | -- | -- | | -- |
| Q024570 | CALM2 | 805 | ENSG00000143933 | 2261844 | 2200503 | -0,1851 | -0,2984 | -- | -- | | -- |
| Q006495 | CALM3 | 808 | ENSG00000198668 | 3462491 | 1882457 | 0,0260 | -0,8276 | -- | -- | | -- |
| Q024571 | CALM3 | 808 | ENSG00000198668 | 3360135 | 3636459 | 0,0108 | 0,1514 | -- | -- | | -- |
| Q006837 | CAMK1 | 8536 | ENSG00000134072 | 3387651 | 3139620 | -0,0119 | -0,0708 | -- | -- | | -- |
| Q024913 | CAMK1 | 8536 | ENSG00000134072 | 3250089 | 3223248 | 0,0381 | 0,0870 | -- | -- | | -- |
| Q007053 | CAMK1D | 57118 | ENSG00000183049 | 3355539 | 2855620 | -0,2409 | -0,3589 | -- | -- | | -- |
| Q025129 | CAMK1D | 57118 | ENSG00000183049 | 4368761 | 3577275 | 0,2442 | -0,0105 | -- | -- | | -- |
| Q007056 | CAMK1G | 57172 | ENSG00000008118 | 3742987 | 3284820 | -0,2043 | -0,1757 | -- | -- | | -- |
| Q025132 | CAMK1G | 57172 | ENSG00000008118 | 3239138 | 2107308 | -0,3075 | -0,7319 | -- | -- | | -- |
| Q006497 | CAMK2A | 815 | ENSG00000070808 | 3630876 | 2892505 | -0,1008 | -0,4137 | -- | -- | | -- |
| Q024573 | CAMK2A | 815 | ENSG00000070808 | 4743598 | 3789436 | 0,2413 | 0,0347 | -- | -- | | -- |
| Q006498 | CAMK2B | 816 | ENSG00000058404 | 2996275 | 1870130 | -0,4405 | -1,1064 | -- | DOWN | | -- |
| Q024574 | CAMK2B | 816 | ENSG00000058404 | 3282035 | 2454637 | -0,3609 | -0,6492 | -- | -- | | -- |
| Q006499 | CAMK2D | 817 | ENSG00000145349 | 3562740 | 1741722 | -0,1846 | -1,1724 | -- | DOWN | | -- |
| Q024575 | CAMK2D | 817 | ENSG00000145349 | 4189125 | 3908569 | 0,0929 | 0,0639 | -- | -- | | -- |
| Q006500 | CAMK2G | 818 | ENSG00000148660 | 4923845 | 3274089 | 0,3901 | -0,1223 | -- | -- | | -- |
| Q024576 | CAMK2G | 818 | ENSG00000148660 | 3358199 | 3130008 | -0,1194 | -0,1662 | -- | -- | | -- |
| Q006496 | CAMK4 | 814 | ENSG00000152495 | 3581053 | 2848067 | -0,0530 | -0,3807 | -- | -- | | -- |
| Q024572 | CAMK4 | 814 | ENSG00000152495 | 4101068 | 4013989 | 0,1148 | 0,1853 | -- | -- | | -- |
| Q025117 | CaMKIINalpha | 55450 | ENSG00000162545 | 2565237 | 1726728 | -0,6885 | -1,0179 | DOWN | DOWN | | DOWN-HIT |
| Q007041 | CaMKIINalpha | 55450 | ENSG00000162545 | 4857382 | 1875110 | 0,1908 | -1,0578 | -- | DOWN | | -- |
| Q007098 | CAMKK1 | 84254 | ENSG00000004660 | 2778792 | 2075261 | -0,3340 | -0,6691 | -- | -- | | -- |
| Q025174 | CAMKK1 | 84254 | ENSG00000004660 | 4066974 | 2696615 | 0,2445 | -0,2652 | -- | -- | | -- |
| Q006923 | CAMKK2 | 10645 | ENSG00000110931 | 3360831 | 2914877 | -0,1748 | -0,3393 | -- | -- | | -- |
| Q024999 | CAMKK2 | 10645 | ENSG00000110931 | 3805015 | 3117446 | -0,0123 | -0,3081 | -- | -- | | -- |
| Q006975 | CARKL | 23729 | ENSG00000197417 | 4865199 | 3786939 | 0,2442 | 0,0360 | -- | -- | | -- |
| Q025051 | CARKL | 23729 | ENSG00000197417 | 4776805 | 2666974 | 0,3312 | -0,3373 | -- | -- | | -- |
| Q006843 | CASK | 8573 | ENSG00000147044 | 3586942 | 2784412 | -0,0822 | -0,3734 | -- | -- | | -- |
| Q024919 | CASK | 8573 | ENSG00000147044 | 3377076 | 2712855 | -0,1343 | -0,4028 | -- | -- | | -- |
| Q006969 | CCRK | 23552 | ENSG00000156345 | 2198392 | 2301168 | -0,5592 | -0,5219 | DOWN | -- | | -- |
| Q025045 | CCRK | 23552 | ENSG00000156345 | 2057928 | 1926974 | -0,4991 | -0,5270 | -- | -- | | -- |
| Q006501 | CDC2 | 983 | ENSG00000170312 | 2977729 | 2079603 | -0,1276 | -0,5419 | -- | -- | | -- |
| Q024577 | CDC2 | 983 | ENSG00000170312 | 3215390 | 4077135 | -0,0085 | 0,3357 | -- | -- | | -- |
| Q006257 | CDC25A | 993 | ENSG00000164045 | 3792412 | 2812032 | 0,1413 | -0,0862 | -- | -- | | -- |
| Q024333 | CDC25A | 993 | ENSG00000164045 | 3924121 | 2102951 | 0,1819 | -0,4642 | -- | -- | | -- |
| Q006258 | CDC25B | 994 | ENSG00000101224 | 2844988 | 2663491 | -0,0430 | -0,2181 | -- | -- | | -- |
| Q024334 | CDC25B | 994 | ENSG00000101224 | 3281182 | 2915775 | 0,1186 | -0,1112 | -- | -- | | -- |
| Q006259 | CDC25C | 995 | ENSG00000158402 | 3773144 | 3429197 | 0,0703 | 0,0036 | -- | -- | | -- |
| Q024335 | CDC25C | 995 | ENSG00000158402 | 4358552 | 2667734 | 0,2543 | -0,3390 | -- | -- | | -- |
| Q006502 | CDC2L1 | 984 | ENSG00000008128 | 1934844 | 2130066 | -0,4334 | -0,4962 | -- | -- | | -- |
| Q024578 | CDC2L1 | 984 | ENSG00000008128 | 1503017 | 2113719 | -0,5686 | -0,3501 | DOWN | -- | | -- |
| Q006846 | CDC2L5 | 8621 | ENSG00000065883 | 3091997 | 2732585 | -0,0288 | -0,3075 | -- | -- | | -- |
| Q024922 | CDC2L5 | 8621 | ENSG00000065883 | 2928025 | 2875628 | -0,0440 | -0,2588 | -- | -- | | -- |
| Q006959 | CDC2L6 | 23097 | ENSG00000155111 | 3866980 | 2524861 | -0,1886 | -0,6370 | -- | -- | | -- |
| Q025035 | CDC2L6 | 23097 | ENSG00000155111 | 4685272 | 3280363 | 0,2340 | -0,1572 | -- | -- | | -- |
| Q006829 | CDC42BPA | 8476 | ENSG00000143776 | 3365495 | 2627290 | -0,0367 | -0,3144 | -- | -- | | -- |
| Q024905 | CDC42BPA | 8476 | ENSG00000143776 | 2420624 | 2275557 | -0,3259 | -0,3189 | -- | -- | | -- |
| Q006890 | CDC42BPB | 9578 | ENSG00000198752 | 4244899 | 2972913 | 0,0561 | -0,3135 | -- | -- | | -- |
| Q024966 | CDC42BPB | 9578 | ENSG00000198752 | 3415639 | 2883727 | -0,1647 | -0,3354 | -- | -- | | -- |
| Q007043 | CDC42BPG | 55561 | ENSG00000171219 | 2823136 | 2579247 | -0,2639 | -0,4009 | -- | -- | | -- |
| Q025119 | CDC42BPG | 55561 | ENSG00000171219 | 2903464 | 2076911 | -0,1183 | -0,4891 | -- | -- | | -- |
| Q007052 | CDC42SE2 | 56990 | ENSG00000158985 | 2609047 | 1720715 | -0,3911 | -0,9959 | -- | DOWN | | -- |
| Q025128 | CDC42SE2 | 56990 | ENSG00000158985 | 3405323 | 3181242 | 0,0397 | -0,1140 | -- | -- | | -- |
| Q006839 | CDK10 | 8558 | ENSG00000185324 | 3355442 | 1923692 | -0,0651 | -0,8627 | -- | -- | | -- |
| Q024915 | CDK10 | 8558 | ENSG00000185324 | 3321701 | 4275961 | -0,0486 | 0,2309 | -- | -- | | -- |
| Q006503 | CDK2 | 1017 | ENSG00000123374 | 4216039 | 3384717 | 0,2934 | -0,0558 | -- | -- | | -- |
| Q024579 | CDK2 | 1017 | ENSG00000123374 | 2831629 | 2851661 | -0,1966 | -0,1705 | -- | -- | | -- |
| Q006504 | CDK3 | 1018 | ENSG00000108504 | 4123822 | 2902719 | 0,1703 | -0,3629 | -- | -- | | -- |
| Q024580 | CDK3 | 1018 | ENSG00000108504 | 2611643 | 2736279 | -0,5060 | -0,3597 | -- | -- | | -- |
| Q006505 | CDK4 | 1019 | ENSG00000135446 | 4513188 | 3677310 | 0,2565 | -0,0507 | -- | -- | | -- |
| Q024581 | CDK4 | 1019 | ENSG00000135446 | 1929740 | 2001493 | -1,1212 | -0,9238 | DOWN | DOWN | | DOWN-HIT |
| Q006506 | CDK5 | 1020 | ENSG00000164885 | 3372863 | 2671463 | -0,2155 | -0,5507 | -- | -- | | -- |
| Q024582 | CDK5 | 1020 | ENSG00000164885 | 4296582 | 4060451 | 0,1089 | 0,1003 | -- | -- | | -- |
| Q006855 | CDK5R1 | 8851 | ENSG00000176749 | 4101037 | 3667362 | 0,1692 | -0,0184 | -- | -- | | -- |
| Q024931 | CDK5R1 | 8851 | ENSG00000176749 | 2191311 | 2308502 | -0,6280 | -0,6982 | DOWN | -- | | -- |
| Q006860 | CDK5R2 | 8941 | ENSG00000171450 | 2784546 | 2880493 | -0,4114 | -0,3127 | -- | -- | | -- |
| Q024936 | CDK5R2 | 8941 | ENSG00000171450 | 3271268 | 2582708 | -0,1114 | -0,4176 | -- | -- | | -- |
| Q006507 | CDK6 | 1021 | ENSG00000105810 | 3792386 | 3387993 | -0,0628 | -0,1741 | -- | -- | | -- |
| Q024583 | CDK6 | 1021 | ENSG00000105810 | 4084113 | 4017847 | 0,0832 | 0,0988 | -- | -- | | -- |
| Q006508 | CDK7 | 1022 | ENSG00000134058 | 3030565 | 2543213 | -0,3109 | -0,4980 | -- | -- | | -- |
| Q024584 | CDK7 | 1022 | ENSG00000134058 | 2698341 | 3008814 | -0,3961 | -0,2282 | -- | -- | | -- |
| Q006509 | CDK8 | 1024 | ENSG00000132964 | 2053434 | 1891610 | -0,5860 | -0,6789 | DOWN | -- | | -- |
| Q024585 | CDK8 | 1024 | ENSG00000132964 | 3259574 | 2928331 | 0,0157 | -0,0817 | -- | -- | | -- |
| Q006510 | CDK9 | 1025 | ENSG00000136807 | 2641161 | 2831699 | -0,1208 | -0,1819 | -- | -- | | -- |
| Q024586 | CDK9 | 1025 | ENSG00000136807 | 1721314 | 2070728 | -0,4758 | -0,3950 | -- | -- | | -- |
| Q006853 | CDKL1 | 8814 | ENSG00000100490 | 3150771 | 2980319 | -0,0914 | -0,1245 | -- | -- | | -- |
| Q024929 | CDKL1 | 8814 | ENSG00000100490 | 3337479 | 3018270 | 0,0595 | -0,0288 | -- | -- | | -- |
| Q006862 | CDKL2 | 8999 | ENSG00000138769 | 2746893 | 2263432 | -0,2545 | -0,6585 | -- | -- | | -- |
| Q024938 | CDKL2 | 8999 | ENSG00000138769 | 3515877 | 3695104 | 0,1156 | 0,0190 | -- | -- | | -- |
| Q007009 | CDKL3 | 51265 | ENSG00000006837 | 2425295 | 2333895 | -0,3072 | -0,3471 | -- | -- | | -- |
| Q025085 | CDKL3 | 51265 | ENSG00000006837 | 1665353 | 1710293 | -0,6035 | -0,5294 | DOWN | -- | | -- |
| Q007159 | CDKL4 | 344387 | NA | 3383253 | 3040539 | -0,0062 | -0,2480 | -- | -- | | -- |
| Q025235 | CDKL4 | 344387 | NA | 4378909 | 3410323 | 0,3001 | -0,0556 | -- | -- | | -- |
| Q006791 | CDKL5 | 6792 | ENSG00000008086 | 3234221 | 2462056 | -0,3299 | -0,6971 | -- | -- | | -- |
| Q024867 | CDKL5 | 6792 | ENSG00000008086 | 3796430 | 2759829 | 0,0328 | -0,3803 | -- | -- | | -- |
| Q006511 | CDKN1A | 1026 | ENSG00000124762 | 3977558 | 3940267 | 0,2204 | 0,1428 | -- | -- | | -- |
| Q024587 | CDKN1A | 1026 | ENSG00000124762 | 4289414 | 3664509 | 0,3394 | 0,1316 | -- | -- | | -- |
| Q006512 | CDKN1B | 1027 | ENSG00000111276 | 3850028 | 3234540 | 0,0900 | -0,2099 | -- | -- | | -- |
| Q024588 | CDKN1B | 1027 | ENSG00000111276 | 2560075 | 1746489 | -0,4907 | -1,0046 | -- | DOWN | | -- |
| Q006513 | CDKN1C | 1028 | ENSG00000129757 | 3871728 | 4017586 | 0,0542 | 0,0816 | -- | -- | | -- |
| Q024589 | CDKN1C | 1028 | ENSG00000129757 | 4082133 | 3188388 | 0,0965 | -0,2289 | -- | -- | | -- |
| Q006514 | CDKN2A | 1029 | ENSG00000147889 | 1983975 | 1709666 | -0,9811 | -1,2494 | DOWN | DOWN | | DOWN-HIT |
| Q024590 | CDKN2A | 1029 | ENSG00000147889 | 4331467 | 3872231 | 0,1717 | 0,0413 | -- | -- | | -- |
| Q006515 | CDKN2B | 1030 | ENSG00000147883 | 4207784 | 3750928 | 0,1384 | -0,0180 | -- | -- | | -- |
| Q024591 | CDKN2B | 1030 | ENSG00000147883 | 4589314 | 3920926 | 0,2903 | 0,0779 | -- | -- | | -- |
| Q006516 | CDKN2C | 1031 | ENSG00000123080 | 3748419 | 3239100 | 0,0332 | -0,1599 | -- | -- | | -- |
| Q024592 | CDKN2C | 1031 | ENSG00000123080 | 3708085 | 3369454 | 0,0654 | -0,0619 | -- | -- | | -- |
| Q006517 | CDKN2D | 1032 | ENSG00000129355 | 1877077 | 2236208 | -0,6714 | -0,4895 | DOWN | -- | | -- |
| Q024593 | CDKN2D | 1032 | ENSG00000129355 | 3512033 | 2767615 | 0,1152 | -0,1567 | -- | -- | | -- |
| Q007067 | CERK | 64781 | ENSG00000100422 | 2933306 | 2304136 | -0,1204 | -0,4122 | -- | -- | | -- |
| Q025143 | CERK | 64781 | ENSG00000100422 | 2316164 | 1830964 | -0,2748 | -0,4606 | -- | -- | | -- |
| Q006518 | CHEK1 | 1111 | ENSG00000149554 | 1974490 | 2485701 | -0,4749 | -0,3936 | -- | -- | | -- |
| Q024594 | CHEK1 | 1111 | ENSG00000149554 | 3034694 | 1885968 | 0,0614 | -0,5453 | -- | -- | | -- |
| Q006939 | CHEK2 | 11200 | ENSG00000183765 | 2909342 | 2727526 | -0,4635 | -0,5706 | -- | -- | | -- |
| Q025015 | CHEK2 | 11200 | ENSG00000183765 | 4010676 | 3658923 | 0,0585 | -0,0895 | -- | -- | | -- |
| Q006519 | CHKA | 1119 | ENSG00000110721 | 3393008 | 3244302 | 0,0064 | -0,1568 | -- | -- | | -- |
| Q024595 | CHKA | 1119 | ENSG00000110721 | 2907716 | 2339835 | -0,1723 | -0,5046 | -- | -- | | -- |
| Q006520 | CHKB | 1120 | ENSG00000100288 | 4472005 | 3780651 | 0,3115 | 0,0045 | -- | -- | | -- |
| Q024596 | CHKB | 1120 | ENSG00000100288 | 2615985 | 2519419 | -0,4427 | -0,5333 | -- | -- | | -- |
| Q006521 | CHUK | 1147 | ENSG00000107566 | 4213063 | 4245559 | 0,1930 | 0,1539 | -- | -- | | -- |
| Q024597 | CHUK | 1147 | ENSG00000107566 | 3172049 | 3121043 | -0,2355 | -0,2741 | -- | -- | | -- |
| Q006919 | CIB2 | 10518 | ENSG00000136425 | 2132210 | 2274845 | -0,5754 | -0,4697 | DOWN | -- | | -- |
| Q024995 | CIB2 | 10518 | ENSG00000136425 | 3431351 | 2735991 | 0,1057 | -0,1130 | -- | -- | | -- |
| Q007121 | CIB3 | 117286 | ENSG00000141977 | 1803341 | 1765747 | -1,5178 | -1,0926 | DOWN | DOWN | | DOWN-HIT |
| Q025197 | CIB3 | 117286 | ENSG00000141977 | 3374413 | 2419952 | -0,3426 | -0,6371 | -- | -- | | -- |
| Q007014 | CINP | 51550 | ENSG00000100865 | 3837248 | 2741107 | 0,0056 | -0,4197 | -- | -- | | -- |
| Q025090 | CINP | 51550 | ENSG00000100865 | 3372363 | 2325311 | -0,1723 | -0,6963 | -- | -- | | -- |
| Q006934 | CIT | 11113 | ENSG00000122966 | 2398510 | 1754359 | -0,5900 | -0,9616 | DOWN | DOWN | | DOWN-HIT |
| Q025010 | CIT | 11113 | ENSG00000122966 | 3570994 | 3263001 | 0,0175 | -0,0789 | -- | -- | | -- |
| Q006522 | CKB | 1152 | ENSG00000166165 | 4014508 | 3530105 | 0,1054 | -0,1309 | -- | -- | | -- |
| Q024598 | CKB | 1152 | ENSG00000166165 | 2394615 | 3204781 | -0,6514 | -0,2403 | DOWN | -- | | -- |
| Q006523 | CKM | 1158 | ENSG00000104879 | 4261253 | 4452222 | 0,1913 | 0,2361 | -- | -- | | -- |
| Q024599 | CKM | 1158 | ENSG00000104879 | 3251896 | 2068368 | -0,1772 | -0,8572 | -- | -- | | -- |
| Q006524 | CKMT1 | 1159 | ENSG00000166998 | 4549314 | 4219221 | 0,3327 | 0,2115 | -- | -- | | -- |
| Q024600 | CKMT1 | 1159 | ENSG00000166998 | 3340205 | 3270648 | -0,0671 | -0,1024 | -- | -- | | -- |
| Q006525 | CKMT2 | 1160 | ENSG00000131730 | 3814991 | 3255044 | 0,2173 | -0,0240 | -- | -- | | -- |
| Q024601 | CKMT2 | 1160 | ENSG00000131730 | 3348109 | 3680564 | 0,0504 | 0,1878 | -- | -- | | -- |
| Q006526 | CKS1B | 1163 | ENSG00000173207 | 2938851 | 2452756 | -0,0664 | -0,4843 | -- | -- | | -- |
| Q024602 | CKS1B | 1163 | ENSG00000173207 | 3326544 | 3087451 | 0,1103 | -0,0404 | -- | -- | | -- |
| Q006527 | CKS2 | 1164 | ENSG00000123975 | 3065419 | 2224985 | -0,1546 | -0,7736 | -- | -- | | -- |
| Q024603 | CKS2 | 1164 | ENSG00000123975 | 3349361 | 3284650 | -0,0386 | -0,1160 | -- | -- | | -- |
| Q006528 | CLK1 | 1195 | ENSG00000013441 | 3653715 | 3249078 | 0,0168 | -0,2702 | -- | -- | | -- |
| Q024604 | CLK1 | 1195 | ENSG00000013441 | 2777009 | 3519636 | -0,3947 | -0,1074 | -- | -- | | -- |
| Q006529 | CLK2 | 1196 | ENSG00000176444 | 3705543 | 3319456 | 0,0082 | -0,2618 | -- | -- | | -- |
| Q024605 | CLK2 | 1196 | ENSG00000176444 | 3179125 | 3722070 | -0,2450 | -0,0596 | -- | -- | | -- |
| Q006530 | CLK3 | 1198 | ENSG00000179335 | 3359846 | 2670409 | -0,1510 | -0,6109 | -- | -- | | -- |
| Q024606 | CLK3 | 1198 | ENSG00000179335 | 2261720 | 2595854 | -0,7465 | -0,6193 | DOWN | -- | | -- |
| Q007057 | CLK4 | 57396 | ENSG00000113240 | 4900655 | 4038010 | 0,2761 | 0,1354 | -- | -- | | -- |
| Q025133 | CLK4 | 57396 | ENSG00000113240 | 4608683 | 2853085 | 0,2945 | -0,2394 | -- | -- | | -- |
| Q006910 | CNKSR1 | 10256 | ENSG00000142675 | 3491013 | 3849501 | -0,1022 | 0,1076 | -- | -- | | -- |
| Q024986 | CNKSR1 | 10256 | ENSG00000142675 | 4175883 | 3916134 | 0,2425 | 0,2227 | -- | -- | | -- |
| Q007017 | CRK7 | 51755 | ENSG00000167258 | 2835841 | 2392030 | -0,1360 | -0,3153 | -- | -- | | -- |
| Q025093 | CRK7 | 51755 | ENSG00000167258 | 3288633 | 2227650 | 0,1163 | -0,2848 | -- | -- | | -- |
| Q006534 | CSK | 1445 | ENSG00000103653 | 2850956 | 2604144 | -0,1601 | -0,4948 | -- | -- | | -- |
| Q024610 | CSK | 1445 | ENSG00000103653 | 3003560 | 2135422 | -0,0968 | -0,6181 | -- | -- | | -- |
| Q006535 | CSNK1A1 | 1452 | ENSG00000113712 | 2846571 | 2579565 | -0,2891 | -0,6419 | -- | -- | | -- |
| Q024611 | CSNK1A1 | 1452 | ENSG00000113712 | 3994672 | 3665182 | 0,1416 | -0,0472 | -- | -- | | -- |
| Q007124 | CSNK1A1L | 122011 | ENSG00000180138 | 4404120 | 2393276 | 0,1945 | -0,4400 | -- | -- | | -- |
| Q025200 | CSNK1A1L | 122011 | ENSG00000180138 | 3229175 | 2210897 | -0,2203 | -0,5609 | -- | -- | | -- |
| Q006536 | CSNK1D | 1453 | ENSG00000141551 | 2537172 | 2009204 | -0,5285 | -1,1560 | -- | DOWN | | -- |
| Q024612 | CSNK1D | 1453 | ENSG00000141551 | 3080693 | 3158661 | -0,3086 | -0,3786 | -- | -- | | -- |
| Q006537 | CSNK1E | 1454 | ENSG00000100181 | 4209507 | 4331389 | 0,1756 | 0,0976 | -- | -- | | -- |
| Q024613 | CSNK1E | 1454 | ENSG00000100181 | 2380256 | 2515377 | -0,7336 | -0,8101 | DOWN | -- | | -- |
| Q007022 | CSNK1G1 | 53944 | ENSG00000169118 | 3951722 | 3832528 | 0,0166 | 0,0176 | -- | -- | | -- |
| Q025098 | CSNK1G1 | 53944 | ENSG00000169118 | 3074277 | 3172997 | -0,3177 | -0,2588 | -- | -- | | -- |
| Q006538 | CSNK1G2 | 1455 | ENSG00000133275 | 3821197 | 3153255 | 0,0238 | -0,4156 | -- | -- | | -- |
| Q024614 | CSNK1G2 | 1455 | ENSG00000133275 | 2615087 | 2267951 | -0,5868 | -0,9700 | DOWN | DOWN | | DOWN-HIT |
| Q006539 | CSNK1G3 | 1456 | ENSG00000151292 | 4309733 | 4201371 | 0,2072 | 0,0822 | -- | -- | | -- |
| Q024615 | CSNK1G3 | 1456 | ENSG00000151292 | 3688025 | 3887521 | -0,0426 | -0,0177 | -- | -- | | -- |
| Q006540 | CSNK2A1 | 1457 | ENSG00000101266 | 3746245 | 3751814 | 0,0549 | -0,0162 | -- | -- | | -- |
| Q024616 | CSNK2A1 | 1457 | ENSG00000101266 | 3431608 | 3538569 | -0,0846 | -0,0637 | -- | -- | | -- |
| Q006541 | CSNK2A2 | 1459 | ENSG00000070770 | 4592999 | 3224319 | 0,4443 | -0,0872 | -- | -- | | -- |
| Q024617 | CSNK2A2 | 1459 | ENSG00000070770 | 3219115 | 2811234 | -0,0678 | -0,2157 | -- | -- | | -- |
| Q006544 | CSNK2B | 1460 | ENSG00000111968 | 2400572 | 1965044 | -0,2419 | -0,4686 | -- | -- | | -- |
| Q024620 | CSNK2B | 1460 | ENSG00000111968 | 3161457 | 2959973 | 0,0409 | -0,0841 | -- | -- | | -- |
| Q006549 | DAPK1 | 1612 | ENSG00000196730 | 4727742 | 2963283 | 0,2894 | -0,2470 | -- | -- | | -- |
| Q024625 | DAPK1 | 1612 | ENSG00000196730 | 3574777 | 2737375 | -0,2975 | -0,4906 | -- | -- | | -- |
| Q006970 | DAPK2 | 23604 | ENSG00000035664 | 3302539 | 2169221 | -0,1068 | -0,7039 | -- | -- | | -- |
| Q025046 | DAPK2 | 23604 | ENSG00000035664 | 1731037 | 2539487 | -0,8112 | -0,4163 | DOWN | -- | | -- |
| Q006550 | DAPK3 | 1613 | ENSG00000167657 | 3775939 | 1813178 | 0,0536 | -0,7795 | -- | -- | | -- |
| Q024626 | DAPK3 | 1613 | ENSG00000167657 | 2938140 | 1351709 | -0,4551 | -1,3869 | -- | DOWN | | -- |
| Q006874 | DCAMKL1 | 9201 | ENSG00000133083 | 4474949 | 3513603 | 0,0587 | -0,1434 | -- | -- | | -- |
| Q024950 | DCAMKL1 | 9201 | ENSG00000133083 | 4107702 | 3745315 | 0,0581 | 0,0310 | -- | -- | | -- |
| Q007141 | DCAMKL2 | 166614 | ENSG00000170390 | 4522130 | 2662410 | 0,4160 | -0,1297 | -- | -- | | -- |
| Q025217 | DCAMKL2 | 166614 | ENSG00000170390 | 2516722 | 1745482 | -0,3028 | -0,5933 | -- | -- | | -- |
| Q006551 | DCK | 1633 | ENSG00000156136 | 3416335 | 2344544 | 0,0819 | -0,3168 | -- | -- | | -- |
| Q024627 | DCK | 1633 | ENSG00000156136 | 4489803 | 3791897 | 0,3422 | 0,1863 | -- | -- | | -- |
| Q006492 | DDR1 | 780 | ENSG00000137332 | 3957723 | 3295859 | 0,0633 | -0,0905 | -- | -- | | -- |
| Q024568 | DDR1 | 780 | ENSG00000137332 | 3738464 | 2474790 | 0,0033 | -0,4768 | -- | -- | | -- |
| Q006658 | DDR2 | 4921 | ENSG00000162733 | 2548972 | 2316694 | -0,2627 | -0,4632 | -- | -- | | -- |
| Q024734 | DDR2 | 4921 | ENSG00000162733 | 3036357 | 2809348 | -0,0017 | -0,1047 | -- | -- | | -- |
| Q006545 | DGKA | 1606 | ENSG00000065357 | 3772309 | 2138544 | 0,1003 | -0,5866 | -- | -- | | -- |
| Q024621 | DGKA | 1606 | ENSG00000065357 | 3426837 | 3085425 | -0,1178 | -0,1535 | -- | -- | | -- |
| Q006546 | DGKB | 1607 | ENSG00000136267 | 1899753 | 1310970 | -1,0886 | -1,4160 | DOWN | DOWN | | DOWN-HIT |
| Q024622 | DGKB | 1607 | ENSG00000136267 | 4433689 | 2536123 | 0,0944 | -0,5357 | -- | -- | | -- |
| Q006836 | DGKD | 8527 | ENSG00000077044 | 3026360 | 3026864 | -0,2856 | -0,2209 | -- | -- | | -- |
| Q024912 | DGKD | 8527 | ENSG00000077044 | 3601229 | 3418915 | 0,0458 | 0,0379 | -- | -- | | -- |
| Q006835 | DGKE | 8526 | ENSG00000153933 | 2347852 | 2079684 | -0,7215 | -0,8026 | DOWN | -- | | -- |
| Q024911 | DGKE | 8526 | ENSG00000153933 | 2695686 | 3137095 | -0,4620 | -0,1804 | -- | -- | | -- |
| Q006547 | DGKG | 1608 | ENSG00000058866 | 4632303 | 2393647 | 0,2233 | -0,6368 | -- | -- | | -- |
| Q024623 | DGKG | 1608 | ENSG00000058866 | 5021508 | 4360506 | 0,2119 | 0,2086 | -- | -- | | -- |
| Q007139 | DGKH | 160851 | ENSG00000102780 | 4781181 | 4025129 | 0,2850 | 0,2266 | -- | -- | | -- |
| Q025215 | DGKH | 160851 | ENSG00000102780 | 4267903 | 3612390 | 0,1782 | 0,1160 | -- | -- | | -- |
| Q006872 | DGKI | 9162 | ENSG00000157680 | 2242158 | 2131894 | -0,5362 | -0,7755 | DOWN | -- | | -- |
| Q024948 | DGKI | 9162 | ENSG00000157680 | 3473527 | 2733247 | 0,0920 | -0,2808 | -- | -- | | -- |
| Q006548 | DGKQ | 1609 | ENSG00000145214 | 4658944 | 3189167 | 0,2267 | -0,2054 | -- | -- | | -- |
| Q024624 | DGKQ | 1609 | ENSG00000145214 | 3274647 | 2892769 | -0,5472 | -0,4276 | DOWN | -- | | -- |
| Q006834 | DGKZ | 8525 | ENSG00000149091 | 3260312 | 2845755 | -0,2481 | -0,3634 | -- | -- | | -- |
| Q024910 | DGKZ | 8525 | ENSG00000149091 | 4354871 | 4077469 | 0,1758 | 0,1032 | -- | -- | | -- |
| Q006552 | DGUOK | 1716 | ENSG00000114956 | 3039397 | 2125072 | 0,0393 | -0,4101 | -- | -- | | -- |
| Q024628 | DGUOK | 1716 | ENSG00000114956 | 1954472 | 1514326 | -0,4237 | -0,7747 | -- | -- | | -- |
| Q006393 | DKFZP566K0524 | 26095 | ENSG00000183675 | 4574603 | 3941511 | 0,2855 | -0,0112 | -- | -- | | -- |
| Q024469 | DKFZP566K0524 | 26095 | ENSG00000183675 | 2790009 | 2232829 | -0,4157 | -0,8807 | -- | DOWN | | -- |
| Q006553 | DMPK | 1760 | ENSG00000104936 | 4482313 | 3730629 | 0,3379 | 0,1179 | -- | -- | | -- |
| Q024629 | DMPK | 1760 | ENSG00000104936 | 3889247 | 3075569 | 0,1111 | -0,0996 | -- | -- | | -- |
| Q006554 | DOK1 | 1796 | ENSG00000115325 | 4980794 | 3258715 | 0,3745 | -0,1727 | -- | -- | | -- |
| Q024630 | DOK1 | 1796 | ENSG00000115325 | 2688323 | 1951620 | -0,6674 | -0,8242 | DOWN | -- | | -- |
| Q006555 | DTYMK | 1841 | ENSG00000168393 | 4303251 | 4235057 | 0,0814 | 0,1672 | -- | -- | | -- |
| Q024631 | DTYMK | 1841 | ENSG00000168393 | 3898800 | 2865654 | -0,1842 | -0,3602 | -- | -- | | -- |
| Q006556 | DYRK1A | 1859 | ENSG00000157540 | 4158723 | 2679740 | 0,0130 | -0,5099 | -- | -- | | -- |
| Q024632 | DYRK1A | 1859 | ENSG00000157540 | 5072948 | 3869825 | 0,2449 | 0,0595 | -- | -- | | -- |
| Q006869 | DYRK1B | 9149 | ENSG00000105204 | 2363353 | 2114707 | -0,5595 | -0,6687 | DOWN | -- | | -- |
| Q024945 | DYRK1B | 9149 | ENSG00000105204 | 2183918 | 1688227 | -0,5183 | -0,8393 | -- | -- | | -- |
| Q006828 | DYRK2 | 8445 | ENSG00000127334 | 3796946 | 3323375 | 0,0000 | -0,1019 | -- | -- | | -- |
| Q024904 | DYRK2 | 8445 | ENSG00000127334 | 3874850 | 3675651 | 0,1321 | 0,1416 | -- | -- | | -- |
| Q006827 | DYRK3 | 8444 | ENSG00000143479 | 3614720 | 3414855 | -0,1349 | -0,1046 | -- | -- | | -- |
| Q024903 | DYRK3 | 8444 | ENSG00000143479 | 2542310 | 2466444 | -0,5690 | -0,5009 | DOWN | -- | | -- |
| Q006852 | DYRK4 | 8798 | ENSG00000010219 | 3442916 | 2654926 | -0,0840 | -0,3850 | -- | -- | | -- |
| Q024928 | DYRK4 | 8798 | ENSG00000010219 | 2940480 | 2499691 | -0,2374 | -0,4225 | -- | -- | | -- |
| Q006996 | EEF2K | 29904 | ENSG00000103319 | 4166784 | 4215050 | 0,1219 | 0,1970 | -- | -- | | -- |
| Q025072 | EEF2K | 29904 | ENSG00000103319 | 4313248 | 4623148 | 0,1744 | 0,2685 | -- | -- | | -- |
| Q006557 | EGFR | 1956 | ENSG00000146648 | 3654998 | 2933298 | -0,1473 | -0,3039 | -- | -- | | -- |
| Q024633 | EGFR | 1956 | ENSG00000146648 | 4614642 | 3046385 | 0,1581 | -0,2862 | -- | -- | | -- |
| Q006987 | EIF2AK1 | 27102 | ENSG00000086232 | 3927608 | 1957078 | 0,0659 | -0,8737 | -- | -- | | -- |
| Q025063 | EIF2AK1 | 27102 | ENSG00000086232 | 3462280 | 2703710 | -0,0704 | -0,4734 | -- | -- | | -- |
| Q006753 | EIF2AK2 | 5610 | ENSG00000055332 | 4689110 | 4610111 | 0,3006 | 0,3107 | -- | -- | | -- |
| Q024829 | EIF2AK2 | 5610 | ENSG00000055332 | 3714366 | 3636311 | 0,0059 | -0,0108 | -- | -- | | -- |
| Q006883 | EIF2AK3 | 9451 | ENSG00000172071 | 5387437 | 4280768 | 0,3253 | 0,1918 | -- | -- | | -- |
| Q024959 | EIF2AK3 | 9451 | ENSG00000172071 | 3408325 | 3406434 | -0,2690 | -0,0821 | -- | -- | | -- |
| Q006988 | EIF2AK4 | 27104 | NA | 3283630 | 2752165 | -0,2643 | -0,4202 | -- | -- | | -- |
| Q025064 | EIF2AK4 | 27104 | NA | 4206487 | 3458076 | 0,1263 | -0,1589 | -- | -- | | -- |
| Q006560 | EPHA1 | 2041 | ENSG00000146904 | 2510957 | 2017599 | -0,1658 | -0,4909 | -- | -- | | -- |
| Q024636 | EPHA1 | 2041 | ENSG00000146904 | 3437684 | 3679847 | 0,2266 | 0,2678 | -- | -- | | -- |
| Q006558 | EPHA2 | 1969 | ENSG00000142627 | 3699112 | 2040035 | -0,0164 | -0,6835 | -- | -- | | -- |
| Q024634 | EPHA2 | 1969 | ENSG00000142627 | 2631361 | 1469305 | -0,5752 | -1,1885 | DOWN | DOWN | | DOWN-HIT |
| Q006561 | EPHA3 | 2042 | ENSG00000044524 | 2102165 | 1803568 | -0,6767 | -0,8675 | DOWN | -- | | -- |
| Q024637 | EPHA3 | 2042 | ENSG00000044524 | 3218121 | 2633984 | -0,1006 | -0,2648 | -- | -- | | -- |
| Q006562 | EPHA4 | 2043 | ENSG00000116106 | 4243340 | 3953157 | 0,1433 | 0,0953 | -- | -- | | -- |
| Q024638 | EPHA4 | 2043 | ENSG00000116106 | 2968550 | 2390115 | -0,4439 | -0,5167 | -- | -- | | -- |
| Q006563 | EPHA5 | 2044 | ENSG00000145242 | 4137205 | 3699719 | 0,0287 | -0,0497 | -- | -- | | -- |
| Q024639 | EPHA5 | 2044 | ENSG00000145242 | 2218448 | 1861766 | -1,1207 | -0,9648 | DOWN | DOWN | | DOWN-HIT |
| Q006564 | EPHA7 | 2045 | ENSG00000135333 | 4341053 | 3101391 | 0,0861 | -0,3144 | -- | -- | | -- |
| Q024640 | EPHA7 | 2045 | ENSG00000135333 | 2592423 | 1786158 | -0,8625 | -1,0711 | DOWN | DOWN | | DOWN-HIT |
| Q006565 | EPHA8 | 2046 | ENSG00000070886 | 3032674 | 2898479 | -0,4636 | -0,3565 | -- | -- | | -- |
| Q024641 | EPHA8 | 2046 | ENSG00000070886 | 3812825 | 2751832 | -0,1066 | -0,4296 | -- | -- | | -- |
| Q006566 | EPHB1 | 2047 | ENSG00000154928 | 3301978 | 3083737 | -0,2037 | -0,1690 | -- | -- | | -- |
| Q024642 | EPHB1 | 2047 | ENSG00000154928 | 2031380 | 1940711 | -0,9017 | -0,8016 | DOWN | -- | | -- |
| Q006567 | EPHB2 | 2048 | ENSG00000133216 | 3820460 | 3207586 | 0,1932 | 0,0184 | -- | -- | | -- |
| Q024643 | EPHB2 | 2048 | ENSG00000133216 | 1756792 | 1476958 | -0,7757 | -0,8736 | DOWN | -- | | -- |
| Q006568 | EPHB3 | 2049 | ENSG00000182580 | 3297451 | 3143540 | 0,1565 | 0,0153 | -- | -- | | -- |
| Q024644 | EPHB3 | 2049 | ENSG00000182580 | 2150530 | 2804778 | -0,2422 | -0,0334 | -- | -- | | -- |
| Q006569 | EPHB4 | 2050 | ENSG00000196411 | 2676770 | 2425100 | -0,3247 | -0,4838 | -- | -- | | -- |
| Q024645 | EPHB4 | 2050 | ENSG00000196411 | 3774219 | 3560129 | 0,1492 | 0,1257 | -- | -- | | -- |
| Q006570 | EPHB6 | 2051 | ENSG00000106123 | 3133513 | 2148463 | -0,2695 | -0,7958 | -- | -- | | -- |
| Q024646 | EPHB6 | 2051 | ENSG00000106123 | 2154463 | 1516097 | -0,8626 | -1,1321 | DOWN | DOWN | | DOWN-HIT |
| Q007025 | EPS8L1 | 54869 | ENSG00000131037 | 3420074 | 2639397 | 0,0567 | -0,2255 | -- | -- | | -- |
| Q025101 | EPS8L1 | 54869 | ENSG00000131037 | 2746969 | 2783017 | -0,1173 | -0,0722 | -- | -- | | -- |
| Q006571 | ERBB2 | 2064 | ENSG00000141736 | 3735069 | 3522997 | -0,0836 | -0,1186 | -- | -- | | -- |
| Q024647 | ERBB2 | 2064 | ENSG00000141736 | 4047062 | 3166501 | -0,0283 | -0,2172 | -- | -- | | -- |
| Q006572 | ERBB3 | 2065 | ENSG00000065361 | 3474686 | 2941683 | -0,2306 | -0,3999 | -- | -- | | -- |
| Q024648 | ERBB3 | 2065 | ENSG00000065361 | 4767045 | 4135865 | 0,2147 | 0,1462 | -- | -- | | -- |
| Q006573 | ERBB4 | 2066 | ENSG00000178568 | 3151224 | 2201285 | -0,3908 | -0,7971 | -- | -- | | -- |
| Q024649 | ERBB4 | 2066 | ENSG00000178568 | 4570430 | 4076018 | 0,2102 | 0,1332 | -- | -- | | -- |
| Q007149 | ERK8 | 225689 | ENSG00000181085 | 2812697 | 2425154 | -0,1615 | -0,2526 | -- | -- | | -- |
| Q025225 | ERK8 | 225689 | ENSG00000181085 | 2706569 | 2098944 | -0,1805 | -0,3897 | -- | -- | | -- |
| Q006574 | ERN1 | 2081 | ENSG00000178607 | 4656217 | 2956366 | 0,3049 | -0,2539 | -- | -- | | -- |
| Q024650 | ERN1 | 2081 | ENSG00000178607 | 2666785 | 2881973 | -0,4604 | -0,2740 | -- | -- | | -- |
| Q006921 | ERN2 | 10595 | ENSG00000134398 | 3443921 | 4191640 | -0,0315 | 0,2150 | -- | -- | | -- |
| Q024997 | ERN2 | 10595 | ENSG00000134398 | 2679503 | 1972687 | -0,3342 | -0,8872 | -- | DOWN | | -- |
| Q007042 | ETNK1 | 55500 | ENSG00000139163 | 3711761 | 2860110 | -0,1033 | -0,3555 | -- | -- | | -- |
| Q025118 | ETNK1 | 55500 | ENSG00000139163 | 3517705 | 3325505 | -0,0602 | -0,0262 | -- | -- | | -- |
| Q007030 | ETNK2 | 55224 | ENSG00000143845 | 3700889 | 3013712 | -0,1397 | -0,4441 | -- | -- | | -- |
| Q025106 | ETNK2 | 55224 | ENSG00000143845 | 4316798 | 2711397 | 0,1723 | -0,5567 | -- | -- | | -- |
| Q006929 | FASTK | 10922 | ENSG00000164896 | 2521087 | 2096181 | -0,4617 | -0,7998 | -- | -- | | -- |
| Q025005 | FASTK | 10922 | ENSG00000164896 | 3554344 | 3345140 | 0,0287 | -0,1356 | -- | -- | | -- |
| Q006576 | FER | 2241 | ENSG00000151422 | 3004686 | 1700350 | 0,0505 | -0,7348 | -- | -- | | -- |
| Q024652 | FER | 2241 | ENSG00000151422 | 1805969 | 1600601 | -0,3994 | -0,6275 | -- | -- | | -- |
| Q006577 | FES | 2242 | ENSG00000182511 | 2660115 | 2494419 | -0,3181 | -0,4640 | -- | -- | | -- |
| Q024653 | FES | 2242 | ENSG00000182511 | 2929007 | 2553371 | -0,1575 | -0,3023 | -- | -- | | -- |
| Q006578 | FGFR1 | 2260 | ENSG00000077782 | 4591706 | 2876837 | 0,3181 | -0,3703 | -- | -- | | -- |
| Q024654 | FGFR1 | 2260 | ENSG00000077782 | 3645035 | 3428418 | -0,0427 | -0,0403 | -- | -- | | -- |
| Q006580 | FGFR2 | 2263 | ENSG00000066468 | 3627054 | 2277368 | -0,1202 | -0,7979 | -- | -- | | -- |
| Q024656 | FGFR2 | 2263 | ENSG00000066468 | 2123512 | 2177328 | -1,0503 | -0,8468 | DOWN | -- | | -- |
| Q006579 | FGFR3 | 2261 | ENSG00000068078 | 3723855 | 3451973 | -0,0431 | -0,1452 | -- | -- | | -- |
| Q024655 | FGFR3 | 2261 | ENSG00000068078 | 4978608 | 3406423 | 0,3318 | -0,1294 | -- | -- | | -- |
| Q006581 | FGFR4 | 2264 | ENSG00000160867 | 4402583 | 3548201 | 0,1558 | -0,1001 | -- | -- | | -- |
| Q024657 | FGFR4 | 2264 | ENSG00000160867 | 2732952 | 2635160 | -0,5405 | -0,5318 | DOWN | -- | | -- |
| Q006582 | FGR | 2268 | ENSG00000000938 | 4100389 | 3206196 | 0,1247 | -0,1617 | -- | -- | | -- |
| Q024658 | FGR | 2268 | ENSG00000000938 | 3769187 | 3000199 | 0,0536 | -0,2309 | -- | -- | | -- |
| Q007032 | FLJ10986 | 55277 | ENSG00000172456 | 3321284 | 1769131 | -0,1721 | -1,0190 | -- | DOWN | | -- |
| Q025108 | FLJ10986 | 55277 | ENSG00000172456 | 3674924 | 3028174 | 0,0791 | -0,1858 | -- | -- | | -- |
| Q007071 | FLJ13052 | 65220 | ENSG00000008130 | 2887571 | 3217943 | -0,4443 | -0,1923 | -- | -- | | -- |
| Q025147 | FLJ13052 | 65220 | ENSG00000008130 | 3271660 | 3678229 | -0,2747 | -0,0781 | -- | -- | | -- |
| Q007102 | FLJ14800 | 84926 | ENSG00000167778 | 4542627 | 4432859 | 0,2116 | 0,1877 | -- | -- | | -- |
| Q025178 | FLJ14800 | 84926 | ENSG00000167778 | 4303103 | 3390238 | 0,1757 | -0,2022 | -- | -- | | -- |
| Q006583 | FLT1 | 2321 | ENSG00000102755 | 2729789 | 2700204 | -0,2373 | -0,2328 | -- | -- | | -- |
| Q024659 | FLT1 | 2321 | ENSG00000102755 | 1921803 | 1977696 | -0,6179 | -0,5450 | DOWN | -- | | -- |
| Q006584 | FLT3 | 2322 | ENSG00000122025 | 3223251 | 2288276 | 0,1177 | -0,4289 | -- | -- | | -- |
| Q024660 | FLT3 | 2322 | ENSG00000122025 | 2219250 | 1746006 | -0,2175 | -0,5591 | -- | -- | | -- |
| Q006585 | FLT3LG | 2323 | ENSG00000090554 | 2666895 | 2259256 | -0,3100 | -0,6241 | -- | -- | | -- |
| Q024661 | FLT3LG | 2323 | ENSG00000090554 | 3371433 | 3073142 | 0,0273 | -0,0853 | -- | -- | | -- |
| Q006586 | FLT4 | 2324 | ENSG00000037280 | 3788852 | 2128247 | 0,0581 | -0,8269 | -- | -- | | -- |
| Q024662 | FLT4 | 2324 | ENSG00000037280 | 2914147 | 2349320 | -0,3294 | -0,5794 | -- | -- | | -- |
| Q007064 | FN3K | 64122 | ENSG00000167363 | 3866909 | 2880522 | -0,0760 | -0,3557 | -- | -- | | -- |
| Q025140 | FN3K | 64122 | ENSG00000167363 | 3861165 | 2708314 | -0,0065 | -0,4165 | -- | -- | | -- |
| Q007078 | FN3KRP | 79672 | ENSG00000141560 | 4490318 | 2287956 | 0,2390 | -0,6800 | -- | -- | | -- |
| Q025154 | FN3KRP | 79672 | ENSG00000141560 | 3384368 | 2613823 | -0,1786 | -0,6060 | -- | -- | | -- |
| Q006587 | FRK | 2444 | ENSG00000111816 | 3753131 | 3924343 | -0,0037 | 0,0471 | -- | -- | | -- |
| Q024663 | FRK | 2444 | ENSG00000111816 | 3879572 | 3485330 | -0,0033 | -0,0979 | -- | -- | | -- |
| Q007142 | FUK | 197258 | ENSG00000157353 | 2594897 | 3204878 | -0,1054 | 0,0091 | -- | -- | | -- |
| Q025218 | FUK | 197258 | ENSG00000157353 | 3002535 | 3439526 | 0,0432 | 0,1054 | -- | -- | | -- |
| Q024664 | FYN | 2534 | ENSG00000010810 | 4156815 | 3492446 | 0,0844 | -0,1203 | -- | -- | | -- |
| Q006588 | FYN | 2534 | ENSG00000010810 | 1867676 | 1842377 | -1,1090 | -1,1315 | DOWN | DOWN | | DOWN-HIT |
| Q006589 | GAK | 2580 | ENSG00000178950 | 3821260 | 3102732 | -0,0300 | -0,3064 | -- | -- | | -- |
| Q024665 | GAK | 2580 | ENSG00000178950 | 2446395 | 2173779 | -0,6519 | -0,8109 | DOWN | -- | | -- |
| Q006590 | GALK1 | 2584 | ENSG00000108479 | 2933807 | 3350473 | -0,3398 | -0,1095 | -- | -- | | -- |
| Q024666 | GALK1 | 2584 | ENSG00000108479 | 2251865 | 2417313 | -0,6223 | -0,5274 | DOWN | -- | | -- |
| Q006591 | GALK2 | 2585 | ENSG00000156958 | 2347731 | 2386293 | -0,4142 | -0,4027 | -- | -- | | -- |
| Q024667 | GALK2 | 2585 | ENSG00000156958 | 3575511 | 2717505 | 0,1349 | -0,1752 | -- | -- | | -- |
| Q006592 | GCK | 2645 | ENSG00000106633 | 2510066 | 2787153 | -0,1920 | -0,2214 | -- | -- | | -- |
| Q024668 | GCK | 2645 | ENSG00000106633 | 2621256 | 2568282 | -0,0771 | -0,1737 | -- | -- | | -- |
| C(GFP) | GFP Control | GFP Control | NA | 3671359 | 3680583 | 0,1281 | 0,0564 | -- | -- | | -- |
| C(GFP) | GFP Control | GFP Control | NA | 3118889 | 3019016 | -0,1039 | -0,2473 | -- | -- | | -- |
| C(GFP) | GFP Control | GFP Control | NA | 3136651 | 2800574 | 0,0178 | -0,2019 | -- | -- | | -- |
| C(GFP) | GFP Control | GFP Control | NA | 2822849 | 3241839 | -0,1310 | -0,0370 | -- | -- | | -- |
| C(GFP) | GFP Control | GFP Control | NA | 3065887 | 2940233 | -0,1878 | -0,2145 | -- | -- | | -- |
| C(GFP) | GFP Control | GFP Control | NA | 2749819 | 3114844 | -0,3746 | -0,1722 | -- | -- | | -- |
| C(GFP) | GFP Control | GFP Control | NA | 3213798 | 2802940 | -0,0621 | -0,1609 | -- | -- | | -- |
| C(GFP) | GFP Control | GFP Control | NA | 2928699 | 2772127 | -0,2199 | -0,2094 | -- | -- | | -- |
| C(GFP) | GFP Control | GFP Control | NA | 3603478 | 3100981 | -0,0721 | -0,2525 | -- | -- | | -- |
| C(GFP) | GFP Control | GFP Control | NA | 3208725 | 2738463 | -0,2893 | -0,5063 | -- | -- | | -- |
| C(GFP) | GFP Control | GFP Control | NA | 3047197 | 2537248 | -0,2292 | -0,4352 | -- | -- | | -- |
| C(GFP) | GFP Control | GFP Control | NA | 2931418 | 2714197 | -0,3365 | -0,4119 | -- | -- | | -- |
| C(GFP) | GFP Control | GFP Control | NA | 3258768 | 3161446 | -0,0530 | -0,1236 | -- | -- | | -- |
| C(GFP) | GFP Control | GFP Control | NA | 3142336 | 2824849 | -0,1211 | -0,3274 | -- | -- | | -- |
| C(GFP) | GFP Control | GFP Control | NA | 2951005 | 2726663 | -0,1099 | -0,2253 | -- | -- | | -- |
| C(GFP) | GFP Control | GFP Control | NA | 2948708 | 2684108 | -0,1329 | -0,2944 | -- | -- | | -- |
| C(GFP) | GFP Control | GFP Control | NA | 3295507 | 2774079 | -0,0010 | -0,2778 | -- | -- | | -- |
| C(GFP) | GFP Control | GFP Control | NA | 3108977 | 2916085 | -0,0635 | -0,1814 | -- | -- | | -- |
| C(GFP) | GFP Control | GFP Control | NA | 2946194 | 2960578 | -0,0605 | -0,0825 | -- | -- | | -- |
| C(GFP) | GFP Control | GFP Control | NA | 2680983 | 2633608 | -0,1616 | -0,1994 | -- | -- | | -- |
| C(GFP) | GFP Control | GFP Control | NA | 3330461 | 2758033 | -0,0439 | -0,2512 | -- | -- | | -- |
| C(GFP) | GFP Control | GFP Control | NA | 2857532 | 2844676 | -0,2443 | -0,1868 | -- | -- | | -- |
| C(GFP) | GFP Control | GFP Control | NA | 2850063 | 2868742 | -0,1846 | -0,0785 | -- | -- | | -- |
| C(GFP) | GFP Control | GFP Control | NA | 2998254 | 2487195 | -0,1211 | -0,2259 | -- | -- | | -- |
| Q006992 | GIT1 | 28964 | ENSG00000108262 | 3509850 | 3477565 | -0,0551 | 0,0006 | -- | -- | | -- |
| Q025068 | GIT1 | 28964 | ENSG00000108262 | 3564665 | 3639534 | 0,0762 | 0,1787 | -- | -- | | -- |
| Q006897 | GIT2 | 9815 | ENSG00000139436 | 3816587 | 2433818 | 0,0652 | -0,5410 | -- | -- | | -- |
| Q024973 | GIT2 | 9815 | ENSG00000139436 | 4081981 | 4214178 | 0,2179 | 0,2259 | -- | -- | | -- |
| Q006593 | GK | 2710 | ENSG00000170095 | 3029141 | 2863901 | -0,1393 | -0,3170 | -- | -- | | -- |
| Q024669 | GK | 2710 | ENSG00000170095 | 2619337 | 2422960 | -0,2909 | -0,4287 | -- | -- | | -- |
| Q006594 | GK2 | 2712 | ENSG00000196475 | 3918690 | 3155279 | 0,1220 | -0,2532 | -- | -- | | -- |
| Q024670 | GK2 | 2712 | ENSG00000196475 | 3213800 | 2420110 | -0,1625 | -0,5667 | -- | -- | | -- |
| Q007087 | GKAP1 | 80318 | ENSG00000165113 | 3677153 | 3003171 | -0,0509 | -0,2913 | -- | -- | | -- |
| Q025163 | GKAP1 | 80318 | ENSG00000165113 | 4186410 | 4972244 | 0,1243 | 0,4004 | -- | -- | | -- |
| Q006904 | GNE | 10020 | ENSG00000159921 | 3316168 | 3403556 | 0,0786 | 0,0061 | -- | -- | | -- |
| Q024980 | GNE | 10020 | ENSG00000159921 | 2440445 | 2650220 | -0,2236 | -0,3261 | -- | -- | | -- |
| Q006767 | GRK1 | 6011 | ENSG00000185974 | 3890248 | 5016671 | 0,0717 | 0,3907 | -- | -- | | -- |
| Q024843 | GRK1 | 6011 | ENSG00000185974 | 3149710 | 3181815 | -0,2530 | -0,2721 | -- | -- | | -- |
| Q024671 | GRK4 | 2868 | ENSG00000125388 | 3736545 | 2881354 | -0,0125 | -0,3821 | -- | -- | | -- |
| Q006595 | GRK4 | 2868 | ENSG00000125388 | 1907390 | 1975246 | -0,9671 | -1,0099 | DOWN | DOWN | | DOWN-HIT |
| Q006596 | GRK5 | 2869 | ENSG00000198873 | 3556888 | 3034928 | -0,0831 | -0,3544 | -- | -- | | -- |
| Q024672 | GRK5 | 2869 | ENSG00000198873 | 3309750 | 2811606 | -0,2003 | -0,4324 | -- | -- | | -- |
| Q006597 | GRK6 | 2870 | ENSG00000198055 | 4142241 | 2646220 | 0,1351 | -0,5443 | -- | -- | | -- |
| Q024673 | GRK6 | 2870 | ENSG00000198055 | 4385552 | 4113024 | 0,2393 | 0,1543 | -- | -- | | -- |
| Q007127 | GRK7 | 131890 | ENSG00000114124 | 3140873 | 2681575 | -0,1967 | -0,3464 | -- | -- | | -- |
| Q025203 | GRK7 | 131890 | ENSG00000114124 | 3939465 | 3990583 | 0,1063 | 0,2000 | -- | -- | | -- |
| Q006599 | GSK3A | 2931 | ENSG00000105723 | 2448828 | 2443598 | -0,3433 | -0,3852 | -- | -- | | -- |
| Q024675 | GSK3A | 2931 | ENSG00000105723 | 3757530 | 2875903 | 0,1997 | -0,1151 | -- | -- | | -- |
| Q006600 | GSK3B | 2932 | ENSG00000082701 | 3028413 | 3314077 | -0,0089 | -0,0427 | -- | -- | | -- |
| Q024676 | GSK3B | 2932 | ENSG00000082701 | 2254101 | 1438098 | -0,2928 | -0,8794 | -- | -- | | -- |
| Q006601 | GUK1 | 2987 | ENSG00000143774 | 3133209 | 2714174 | -0,1099 | -0,4400 | -- | -- | | -- |
| Q024677 | GUK1 | 2987 | ENSG00000143774 | 2614613 | 2707728 | -0,3298 | -0,3453 | -- | -- | | -- |
| Q024678 | HCK | 3055 | ENSG00000101336 | 4171798 | 3629067 | 0,1954 | -0,0298 | -- | -- | | -- |
| Q006602 | HCK | 3055 | ENSG00000101336 | 2057473 | 1951383 | -0,7786 | -1,0284 | DOWN | DOWN | | DOWN-HIT |
| Q006868 | HGS | 9146 | ENSG00000185359 | 3596454 | 3647404 | -0,1075 | -0,0453 | -- | -- | | -- |
| Q024944 | HGS | 9146 | ENSG00000185359 | 2659784 | 3310994 | -0,4188 | -0,1244 | -- | -- | | -- |
| Q007147 | HIPK1 | 204851 | ENSG00000163349 | 2327412 | 1794374 | -0,7645 | -0,8917 | DOWN | DOWN | | DOWN-HIT |
| Q025223 | HIPK1 | 204851 | ENSG00000163349 | 3296374 | 2083274 | -0,1668 | -0,6285 | -- | -- | | -- |
| Q006993 | HIPK2 | 28996 | ENSG00000064393 | 2263842 | 2049639 | -0,4085 | -0,5374 | -- | -- | | -- |
| Q025069 | HIPK2 | 28996 | ENSG00000064393 | 3082273 | 2497937 | 0,0533 | -0,1302 | -- | -- | | -- |
| Q006906 | HIPK3 | 10114 | ENSG00000110422 | 3537424 | 2698668 | -0,1050 | -0,4373 | -- | -- | | -- |
| Q024982 | HIPK3 | 10114 | ENSG00000110422 | 3340360 | 2438876 | -0,1730 | -0,6920 | -- | -- | | -- |
| Q007133 | HIPK4 | 147746 | ENSG00000160396 | 3142816 | 1378369 | -0,0879 | -0,8538 | -- | -- | | -- |
| Q025209 | HIPK4 | 147746 | ENSG00000160396 | 3870392 | 1681733 | 0,1893 | -0,6767 | -- | -- | | -- |
| Q006603 | HK1 | 3098 | ENSG00000156515 | 2664928 | 2651129 | -0,4650 | -0,5866 | -- | -- | | -- |
| Q024679 | HK1 | 3098 | ENSG00000156515 | 2903316 | 1614227 | -0,3625 | -1,3173 | -- | DOWN | | -- |
| Q006604 | HK2 | 3099 | ENSG00000159399 | 3204475 | 2796911 | -0,2193 | -0,5079 | -- | -- | | -- |
| Q024680 | HK2 | 3099 | ENSG00000159399 | 3481414 | 2968367 | -0,1078 | -0,3750 | -- | -- | | -- |
| Q006605 | HK3 | 3101 | ENSG00000160883 | 3552607 | 2552901 | -0,0686 | -0,6209 | -- | -- | | -- |
| Q024681 | HK3 | 3101 | ENSG00000160883 | 3381206 | 3334887 | -0,1244 | -0,1664 | -- | -- | | -- |
| Q006981 | HSPB8 | 26353 | ENSG00000152137 | 4447982 | 3265085 | 0,0939 | -0,1754 | -- | -- | | -- |
| Q025057 | HSPB8 | 26353 | ENSG00000152137 | 4295097 | 3408174 | 0,1157 | -0,1185 | -- | -- | | -- |
| Q007002 | HUNK | 30811 | ENSG00000142149 | 1996156 | 2520937 | -0,6254 | -0,4485 | DOWN | -- | | -- |
| Q025078 | HUNK | 30811 | ENSG00000142149 | 3276231 | 2453562 | 0,0126 | -0,5142 | -- | -- | | -- |
| Q006979 | IBTK | 25998 | ENSG00000005700 | 3233834 | 2521999 | -0,2628 | -0,5267 | -- | -- | | -- |
| Q025055 | IBTK | 25998 | ENSG00000005700 | 3096774 | 2695544 | -0,2384 | -0,4341 | -- | -- | | -- |
| Q006949 | ICK | 22858 | ENSG00000112144 | 4195329 | 3133762 | 0,0309 | -0,4196 | -- | -- | | -- |
| Q025025 | ICK | 22858 | ENSG00000112144 | 3797993 | 3889628 | -0,0057 | -0,0001 | -- | -- | | -- |
| Q006896 | IHPK1 | 9807 | ENSG00000176095 | 3378152 | 3451833 | 0,0842 | 0,0156 | -- | -- | | -- |
| Q024972 | IHPK1 | 9807 | ENSG00000176095 | 2820503 | 3067905 | -0,0711 | -0,1210 | -- | -- | | -- |
| Q007012 | IHPK2 | 51447 | ENSG00000068745 | 4299399 | 4372508 | 0,1795 | 0,2360 | -- | -- | | -- |
| Q025088 | IHPK2 | 51447 | ENSG00000068745 | 4935973 | 3567942 | 0,3879 | -0,0897 | -- | -- | | -- |
| Q007120 | IHPK3 | 117283 | ENSG00000161896 | 2486379 | 1833521 | -0,7744 | -1,0156 | DOWN | DOWN | | DOWN-HIT |
| Q025196 | IHPK3 | 117283 | ENSG00000161896 | 3080450 | 2342432 | -0,4161 | -0,6791 | -- | -- | | -- |
| Q006833 | IKBKAP | 8518 | ENSG00000070061 | 3406489 | 2295449 | -0,1777 | -0,6794 | -- | -- | | -- |
| Q024909 | IKBKAP | 8518 | ENSG00000070061 | 4537387 | 3434932 | 0,2332 | -0,1960 | -- | -- | | -- |
| Q006606 | IKBKB | 3551 | ENSG00000104365 | 3307268 | 3263524 | -0,1143 | -0,1690 | -- | -- | | -- |
| Q024682 | IKBKB | 3551 | ENSG00000104365 | 3516116 | 3978557 | -0,0058 | 0,1619 | -- | -- | | -- |
| Q006893 | IKBKE | 9641 | ENSG00000143466 | 4964005 | 3322311 | 0,2673 | -0,1583 | -- | -- | | -- |
| Q024969 | IKBKE | 9641 | ENSG00000143466 | 3342206 | 2284671 | -0,2212 | -0,5810 | -- | -- | | -- |
| Q006832 | IKBKG | 8517 | ENSG00000073009 | 2850668 | 2664174 | -0,3954 | -0,4538 | -- | -- | | -- |
| Q024908 | IKBKG | 8517 | ENSG00000073009 | 3707030 | 3422660 | -0,0158 | -0,1711 | -- | -- | | -- |
| Q006607 | ILK | 3611 | ENSG00000166333 | 2161544 | 1770567 | -0,4885 | -0,8088 | -- | -- | | -- |
| Q024683 | ILK | 3611 | ENSG00000166333 | 3227656 | 2323985 | -0,0074 | -0,3954 | -- | -- | | -- |
| Q006608 | IRAK1 | 3654 | ENSG00000184216 | 3870900 | 3909842 | 0,2525 | 0,1263 | -- | -- | | -- |
| Q024684 | IRAK1 | 3654 | ENSG00000184216 | 2589505 | 2320985 | -0,2250 | -0,4450 | -- | -- | | -- |
| Q007128 | IRAK1BP1 | 134728 | ENSG00000146243 | 4998227 | 3540290 | 0,3376 | -0,0091 | -- | -- | | -- |
| Q025204 | IRAK1BP1 | 134728 | ENSG00000146243 | 4305692 | 4105723 | 0,1034 | 0,2082 | -- | -- | | -- |
| Q006609 | IRAK2 | 3656 | ENSG00000134070 | 2816380 | 3137821 | -0,2849 | -0,2947 | -- | -- | | -- |
| Q024685 | IRAK2 | 3656 | ENSG00000134070 | 2361487 | 3224577 | -0,5354 | -0,1860 | DOWN | -- | | -- |
| Q006940 | IRAK3 | 11213 | ENSG00000090376 | 4199319 | 2250117 | 0,0773 | -0,8634 | -- | -- | | -- |
| Q025016 | IRAK3 | 11213 | ENSG00000090376 | 3724826 | 2677904 | -0,0531 | -0,5648 | -- | -- | | -- |
| Q007007 | IRAK4 | 51135 | ENSG00000198001 | 3588169 | 3585812 | -0,0612 | 0,0003 | -- | -- | | -- |
| Q025083 | IRAK4 | 51135 | ENSG00000198001 | 2858877 | 2512494 | -0,3220 | -0,4509 | -- | -- | | -- |
| Q006610 | ITK | 3702 | ENSG00000113263 | 4167161 | 3502363 | 0,1948 | -0,1854 | -- | -- | | -- |
| Q024686 | ITK | 3702 | ENSG00000113263 | 3883311 | 4024216 | 0,0574 | 0,0557 | -- | -- | | -- |
| Q006611 | ITPK1 | 3705 | ENSG00000100605 | 4560500 | 3136766 | 0,3020 | -0,3862 | -- | -- | | -- |
| Q024687 | ITPK1 | 3705 | ENSG00000100605 | 4552478 | 4216112 | 0,2607 | 0,0961 | -- | -- | | -- |
| Q024688 | ITPKA | 3706 | ENSG00000137825 | 2458961 | 2748800 | -0,6474 | -0,5774 | DOWN | -- | | -- |
| Q006612 | ITPKA | 3706 | ENSG00000137825 | 2369761 | 1973938 | -0,6710 | -1,1616 | DOWN | DOWN | | DOWN-HIT |
| Q006613 | ITPKB | 3707 | ENSG00000143772 | 3977955 | 3713968 | 0,0939 | -0,0863 | -- | -- | | -- |
| Q024689 | ITPKB | 3707 | ENSG00000143772 | 4026726 | 3704693 | 0,1020 | -0,0595 | -- | -- | | -- |
| Q007085 | ITPKC | 80271 | ENSG00000086544 | 3556259 | 2726405 | -0,0342 | -0,4162 | -- | -- | | -- |
| Q025161 | ITPKC | 80271 | ENSG00000086544 | 3511174 | 3811592 | -0,0353 | 0,0378 | -- | -- | | -- |
| Q006614 | JAK1 | 3716 | ENSG00000162434 | 4241402 | 3078761 | 0,2331 | -0,2901 | -- | -- | | -- |
| Q024690 | JAK1 | 3716 | ENSG00000162434 | 2686354 | 3144704 | -0,4044 | -0,2087 | -- | -- | | -- |
| Q006615 | JAK2 | 3717 | ENSG00000096968 | 3699714 | 2825859 | 0,1681 | -0,2467 | -- | -- | | -- |
| Q024691 | JAK2 | 3717 | ENSG00000096968 | 3580236 | 2708799 | 0,0904 | -0,2409 | -- | -- | | -- |
| Q006616 | JAK3 | 3718 | ENSG00000105639 | 3045973 | 2366946 | -0,1085 | -0,6937 | -- | -- | | -- |
| Q024692 | JAK3 | 3718 | ENSG00000105639 | 2292571 | 2234008 | -0,4927 | -0,6401 | -- | -- | | -- |
| Q006617 | KDR | 3791 | ENSG00000128052 | 3865554 | 3284685 | 0,1155 | -0,3035 | -- | -- | | -- |
| Q024693 | KDR | 3791 | ENSG00000128052 | 2893013 | 2918107 | -0,3537 | -0,4621 | -- | -- | | -- |
| Q006618 | KHK | 3795 | ENSG00000138030 | 2793279 | 2448435 | -0,4095 | -0,8769 | -- | -- | | -- |
| Q024694 | KHK | 3795 | ENSG00000138030 | 4185298 | 3247940 | 0,1102 | -0,4046 | -- | -- | | -- |
| Q007060 | KIAA1446 | 57596 | ENSG00000183092 | 3410030 | 4246325 | -0,0040 | 0,2622 | -- | -- | | -- |
| Q025136 | KIAA1446 | 57596 | ENSG00000183092 | 3697357 | 3650140 | 0,1608 | 0,0622 | -- | -- | | -- |
| Q007100 | KIAA1804 | 84451 | ENSG00000143674 | 3194817 | 2897011 | -0,1246 | -0,3453 | -- | -- | | -- |
| Q025176 | KIAA1804 | 84451 | ENSG00000143674 | 3673933 | 3521073 | 0,1156 | -0,0614 | -- | -- | | -- |
| Q007058 | KIDINS220 | 57498 | ENSG00000134313 | 4668446 | 3511271 | 0,2941 | -0,0072 | -- | -- | | -- |
| Q025134 | KIDINS220 | 57498 | ENSG00000134313 | 4278826 | 3360836 | 0,2846 | 0,0668 | -- | -- | | -- |
| Q006619 | KIT | 3815 | ENSG00000157404 | 3273557 | 2876033 | -0,2090 | -0,6299 | -- | -- | | -- |
| Q024695 | KIT | 3815 | ENSG00000157404 | 4316670 | 3989876 | 0,1338 | -0,0940 | -- | -- | | -- |
| Q006854 | KSR | 8844 | NA | 3300204 | 3445517 | 0,0175 | -0,0269 | -- | -- | | -- |
| Q024930 | KSR | 8844 | NA | 2584055 | 2942925 | -0,2166 | -0,2583 | -- | -- | | -- |
| Q007155 | KSR2 | 283455 | ENSG00000171435 | 3236442 | 3128471 | -0,2253 | -0,1784 | -- | -- | | -- |
| Q025231 | KSR2 | 283455 | ENSG00000171435 | 2863533 | 3015972 | -0,3479 | -0,1807 | -- | -- | | -- |
| Q006867 | LATS1 | 9113 | ENSG00000131023 | 4165524 | 4060971 | 0,0531 | 0,0480 | -- | -- | | -- |
| Q024943 | LATS1 | 9113 | ENSG00000131023 | 4453088 | 4419918 | 0,2375 | 0,2225 | -- | -- | | -- |
| Q006982 | LATS2 | 26524 | ENSG00000150457 | 3820491 | 3041134 | -0,1321 | -0,2814 | -- | -- | | -- |
| Q025058 | LATS2 | 26524 | ENSG00000150457 | 4493489 | 3418206 | 0,2088 | -0,0671 | -- | -- | | -- |
| Q006620 | LCK | 3932 | ENSG00000182866 | 4125234 | 4216709 | 0,1262 | 0,0254 | -- | -- | | -- |
| Q024696 | LCK | 3932 | ENSG00000182866 | 3227402 | 2385458 | -0,3025 | -0,9730 | -- | DOWN | | -- |
| Q006621 | LIMK1 | 3984 | ENSG00000106683 | 2720132 | 2360403 | -0,4790 | -0,9017 | -- | DOWN | | -- |
| Q024697 | LIMK1 | 3984 | ENSG00000106683 | 2499746 | 1855545 | -0,6476 | -1,3076 | DOWN | DOWN | | DOWN-HIT |
| Q006622 | LIMK2 | 3985 | ENSG00000182541 | 4505857 | 3863905 | 0,3119 | 0,0060 | -- | -- | | -- |
| Q024698 | LIMK2 | 3985 | ENSG00000182541 | 2972877 | 3741253 | -0,3102 | -0,0111 | -- | -- | | -- |
| Q006948 | LMTK2 | 22853 | ENSG00000164715 | 3412201 | 3351167 | -0,3138 | -0,3580 | -- | -- | | -- |
| Q025024 | LMTK2 | 22853 | ENSG00000164715 | 3856978 | 2924233 | -0,0181 | -0,5005 | -- | -- | | -- |
| Q007115 | LMTK3 | 114783 | ENSG00000142235 | 2188094 | 2243294 | -0,6040 | -0,5357 | DOWN | -- | | -- |
| Q025191 | LMTK3 | 114783 | ENSG00000142235 | 1812907 | 1925235 | -0,6558 | -0,5996 | DOWN | -- | | -- |
| Q007140 | LOC161635 | 161635 | NA | 4749968 | 3287746 | 0,3590 | 0,0221 | -- | -- | | -- |
| Q025216 | LOC161635 | 161635 | NA | 4663717 | 3707817 | 0,3859 | 0,2109 | -- | -- | | -- |
| Q007154 | LOC283155 | 283155 | ENSG00000185932 | 2381799 | 1992385 | -0,7453 | -0,8692 | DOWN | -- | | -- |
| Q025230 | LOC283155 | 283155 | ENSG00000185932 | 3416119 | 2997789 | -0,2438 | -0,2568 | -- | -- | | -- |
| Q007157 | LOC285940 | 285940 | NA | 4089495 | 3691886 | 0,3195 | 0,2314 | -- | -- | | -- |
| Q025233 | LOC285940 | 285940 | NA | 3348037 | 3075553 | 0,0882 | 0,0385 | -- | -- | | -- |
| Q007160 | LOC375449 | 375449 | ENSG00000196567 | 4697421 | 3682112 | 0,3357 | -0,0345 | -- | -- | | -- |
| Q025236 | LOC375449 | 375449 | ENSG00000196567 | 4217132 | 3329554 | 0,1128 | -0,1659 | -- | -- | | -- |
| Q007162 | LOC390777 | 390777 | NA | 3272576 | 2771709 | -0,2389 | -0,4702 | -- | -- | | -- |
| Q025238 | LOC390777 | 390777 | NA | 2854198 | 2731219 | -0,5164 | -0,4726 | -- | -- | | -- |
| Q007163 | LOC390975 | 390975 | ENSG00000187550 | 3656254 | 3108877 | -0,0378 | -0,2510 | -- | -- | | -- |
| Q025239 | LOC390975 | 390975 | ENSG00000187550 | 2595895 | 2863218 | -0,4927 | -0,3202 | -- | -- | | -- |
| Q007164 | LOC391295 | 391295 | NA | 3996148 | 3455492 | 0,1721 | -0,0086 | -- | -- | | -- |
| Q025240 | LOC391295 | 391295 | NA | 3040589 | 2862572 | -0,1452 | -0,2110 | -- | -- | | -- |
| Q007165 | LOC391533 | 391533 | NA | 2452921 | 2756953 | -0,3056 | -0,1653 | -- | -- | | -- |
| Q025241 | LOC391533 | 391533 | NA | 2842905 | 2623072 | -0,1137 | -0,1797 | -- | -- | | -- |
| Q025242 | LOC392265 | 392265 | NA | 1434492 | 1663784 | -0,7044 | -0,9467 | DOWN | DOWN | | DOWN-HIT |
| Q007166 | LOC392265 | 392265 | NA | 2319672 | 2570698 | -0,2195 | -0,3854 | -- | -- | | -- |
| Q007167 | LOC400301 | 400301 | NA | 3240582 | 3218827 | -0,0619 | -0,2240 | -- | -- | | -- |
| Q025243 | LOC400301 | 400301 | NA | 2474553 | 2646396 | -0,4560 | -0,4848 | -- | -- | | -- |
| Q007168 | LOC407835 | 407835 | NA | 4298674 | 3552561 | 0,2060 | -0,1685 | -- | -- | | -- |
| Q025244 | LOC407835 | 407835 | NA | 3832111 | 3814467 | -0,0136 | -0,0264 | -- | -- | | -- |
| Q006459 | LOC474338 | 474338 | NA | 2854013 | 2491493 | -0,3576 | -0,5846 | -- | -- | | -- |
| Q024535 | LOC474338 | 474338 | NA | 4022284 | 3372546 | 0,1646 | -0,0821 | -- | -- | | -- |
| Q007048 | LOC55971 | 55971 | ENSG00000006453 | 3392590 | 3013937 | -0,4401 | -0,3244 | -- | -- | | -- |
| Q025124 | LOC55971 | 55971 | ENSG00000006453 | 3704590 | 2249714 | -0,1365 | -0,6408 | -- | -- | | -- |
| Q007111 | LOC91807 | 91807 | ENSG00000140795 | 4402380 | 3131728 | 0,0882 | -0,4819 | -- | -- | | -- |
| Q025187 | LOC91807 | 91807 | ENSG00000140795 | 4207444 | 3990450 | 0,1096 | -0,0067 | -- | -- | | -- |
| Q007079 | LRRK1 | 79705 | ENSG00000154237 | 3589057 | 2700305 | -0,0924 | -0,4385 | -- | -- | | -- |
| Q025155 | LRRK1 | 79705 | ENSG00000154237 | 4343841 | 3615520 | 0,1694 | -0,1014 | -- | -- | | -- |
| Q007123 | LRRK2 | 120892 | ENSG00000188906 | 4382899 | 2854789 | 0,0971 | -0,2960 | -- | -- | | -- |
| Q025199 | LRRK2 | 120892 | ENSG00000188906 | 4017278 | 2819451 | 0,0227 | -0,3126 | -- | -- | | -- |
| Q006623 | LTK | 4058 | ENSG00000062524 | 2971901 | 2176128 | -0,1321 | -0,6391 | -- | -- | | -- |
| Q024699 | LTK | 4058 | ENSG00000062524 | 2883083 | 2627003 | -0,2377 | -0,3314 | -- | -- | | -- |
| Q007112 | LYK5 | 92335 | ENSG00000125695 | 3221200 | 3511511 | -0,3981 | -0,2619 | -- | -- | | -- |
| Q025188 | LYK5 | 92335 | ENSG00000125695 | 3856668 | 2596461 | -0,0050 | -0,6727 | -- | -- | | -- |
| Q006626 | LYN | 4067 | ENSG00000147507 | 4017698 | 3278419 | 0,2666 | 0,0465 | -- | -- | | -- |
| Q024702 | LYN | 4067 | ENSG00000147507 | 2284613 | 1788301 | -0,5637 | -0,8455 | DOWN | -- | | -- |
| Q006841 | MADD | 8567 | ENSG00000110514 | 3596877 | 4460218 | -0,0791 | 0,2787 | -- | -- | | -- |
| Q024917 | MADD | 8567 | ENSG00000110514 | 3955351 | 4269802 | 0,0406 | 0,1586 | -- | -- | | -- |
| Q007137 | MAGI1 | 154043 | ENSG00000153721 | 4222435 | 3091798 | 0,0253 | -0,1890 | -- | -- | | -- |
| Q025213 | MAGI1 | 154043 | ENSG00000153721 | 3573420 | 2572664 | -0,2415 | -0,4202 | -- | -- | | -- |
| Q007152 | MAGI-3 | 260425 | ENSG00000081026 | 4730821 | 3294203 | 0,3349 | -0,1500 | -- | -- | | -- |
| Q025228 | MAGI-3 | 260425 | ENSG00000081026 | 3388657 | 2714017 | -0,2288 | -0,4151 | -- | -- | | -- |
| Q006628 | MAK | 4117 | ENSG00000111837 | 3847460 | 3222892 | -0,0240 | -0,1631 | -- | -- | | -- |
| Q024704 | MAK | 4117 | ENSG00000111837 | 5316666 | 3096848 | 0,3235 | -0,3254 | -- | -- | | -- |
| Q006747 | MAP2K1 | 5604 | ENSG00000169032 | 3200503 | 3224318 | 0,0989 | 0,0764 | -- | -- | | -- |
| Q024823 | MAP2K1 | 5604 | ENSG00000169032 | 1836857 | 1694054 | -0,5251 | -0,5629 | -- | -- | | -- |
| Q006847 | MAP2K1IP1 | 8649 | ENSG00000109270 | 2191337 | 1574499 | -0,6234 | -1,1743 | DOWN | DOWN | | DOWN-HIT |
| Q024923 | MAP2K1IP1 | 8649 | ENSG00000109270 | 3588590 | 2607818 | 0,0490 | -0,4873 | -- | -- | | -- |
| Q006748 | MAP2K2 | 5605 | ENSG00000126934 | 3255092 | 4682926 | 0,0310 | 0,4332 | -- | -- | | -- |
| Q024824 | MAP2K2 | 5605 | ENSG00000126934 | 1860021 | 3048652 | -0,5481 | -0,0248 | DOWN | -- | | -- |
| Q006749 | MAP2K3 | 5606 | ENSG00000034152 | 4254069 | 4876640 | 0,2594 | 0,4189 | -- | -- | | -- |
| Q024825 | MAP2K3 | 5606 | ENSG00000034152 | 2270914 | 2449167 | -0,5800 | -0,4526 | DOWN | -- | | -- |
| Q006777 | MAP2K4 | 6416 | ENSG00000065559 | 4309434 | 4480760 | 0,2262 | 0,2291 | -- | -- | | -- |
| Q024853 | MAP2K4 | 6416 | ENSG00000065559 | 3433508 | 2975528 | -0,1023 | -0,3498 | -- | -- | | -- |
| Q006750 | MAP2K5 | 5607 | ENSG00000137764 | 4164224 | 4371366 | 0,1672 | 0,2191 | -- | -- | | -- |
| Q024826 | MAP2K5 | 5607 | ENSG00000137764 | 3658744 | 1825866 | -0,0572 | -1,0037 | -- | DOWN | | -- |
| Q006751 | MAP2K6 | 5608 | ENSG00000108984 | 3969092 | 4663430 | 0,0657 | 0,3004 | -- | -- | | -- |
| Q024827 | MAP2K6 | 5608 | ENSG00000108984 | 3027767 | 2726318 | -0,3982 | -0,4843 | -- | -- | | -- |
| Q006752 | MAP2K7 | 5609 | ENSG00000076984 | 4554391 | 4537061 | 0,2407 | 0,2561 | -- | -- | | -- |
| Q024828 | MAP2K7 | 5609 | ENSG00000076984 | 3285005 | 2550513 | -0,2532 | -0,5927 | -- | -- | | -- |
| Q006632 | MAP3K1 | 4214 | ENSG00000095015 | 3830919 | 2443769 | 0,1698 | -0,3211 | -- | -- | | -- |
| Q024708 | MAP3K1 | 4214 | ENSG00000095015 | 2395734 | 2324724 | -0,6652 | -0,5852 | DOWN | -- | | -- |
| Q006638 | MAP3K10 | 4294 | ENSG00000130758 | 4856226 | 4069787 | 0,2867 | 0,1516 | -- | -- | | -- |
| Q024714 | MAP3K10 | 4294 | ENSG00000130758 | 5230128 | 4301977 | 0,3056 | 0,1989 | -- | -- | | -- |
| Q006639 | MAP3K11 | 4296 | ENSG00000173327 | 3479509 | 2974780 | -0,1551 | -0,2195 | -- | -- | | -- |
| Q024715 | MAP3K11 | 4296 | ENSG00000173327 | 3799181 | 2880467 | -0,1143 | -0,3488 | -- | -- | | -- |
| Q006818 | MAP3K12 | 7786 | ENSG00000139625 | 4197422 | 1662299 | 0,0038 | -1,1503 | -- | DOWN | | -- |
| Q024894 | MAP3K12 | 7786 | ENSG00000139625 | 3866331 | 2978676 | -0,0406 | -0,2952 | -- | -- | | -- |
| Q006873 | MAP3K13 | 9175 | ENSG00000073803 | 4253538 | 3299560 | 0,1031 | -0,2121 | -- | -- | | -- |
| Q024949 | MAP3K13 | 9175 | ENSG00000073803 | 3501546 | 3102542 | -0,0629 | -0,1993 | -- | -- | | -- |
| Q006863 | MAP3K14 | 9020 | ENSG00000006062 | 3202106 | 2277476 | -0,2138 | -0,8029 | -- | -- | | -- |
| Q024939 | MAP3K14 | 9020 | ENSG00000006062 | 3692109 | 3424975 | 0,0477 | -0,1535 | -- | -- | | -- |
| Q006926 | MAP3K2 | 10746 | ENSG00000169967 | 4423367 | 2592257 | 0,2631 | -0,4132 | -- | -- | | -- |
| Q025002 | MAP3K2 | 10746 | ENSG00000169967 | 3528857 | 3077068 | 0,0151 | -0,1306 | -- | -- | | -- |
| Q006633 | MAP3K3 | 4215 | ENSG00000198909 | 3146905 | 2729345 | 0,1058 | -0,0618 | -- | -- | | -- |
| Q024709 | MAP3K3 | 4215 | ENSG00000198909 | 2155607 | 1616896 | -0,5036 | -0,8000 | -- | -- | | -- |
| Q006634 | MAP3K4 | 4216 | ENSG00000085511 | 2644370 | 2969721 | -0,2621 | -0,0999 | -- | -- | | -- |
| Q024710 | MAP3K4 | 4216 | ENSG00000085511 | 3933906 | 2169485 | 0,2133 | -0,5038 | -- | -- | | -- |
| Q006635 | MAP3K5 | 4217 | ENSG00000197442 | 4204079 | 3884578 | 0,1802 | 0,1340 | -- | -- | | -- |
| Q024711 | MAP3K5 | 4217 | ENSG00000197442 | 2558701 | 1971328 | -0,6482 | -0,7819 | DOWN | -- | | -- |
| Q006865 | MAP3K6 | 9064 | ENSG00000142733 | 4003402 | 2844818 | -0,0160 | -0,5671 | -- | -- | | -- |
| Q024941 | MAP3K6 | 9064 | ENSG00000142733 | 2302112 | 1882097 | -0,7935 | -1,1978 | DOWN | DOWN | | DOWN-HIT |
| Q006796 | MAP3K7 | 6885 | ENSG00000135341 | 4156583 | 2256000 | -0,0120 | -0,7726 | -- | -- | | -- |
| Q024872 | MAP3K7 | 6885 | ENSG00000135341 | 3456495 | 2600350 | -0,1822 | -0,4554 | -- | -- | | -- |
| Q006916 | MAP3K7IP1 | 10454 | ENSG00000100324 | 1575045 | 1904769 | -1,2755 | -0,9321 | DOWN | DOWN | | DOWN-HIT |
| Q024992 | MAP3K7IP1 | 10454 | ENSG00000100324 | 3817961 | 3317677 | -0,0151 | -0,2059 | -- | -- | | -- |
| Q006960 | MAP3K7IP2 | 23118 | ENSG00000055208 | 3033903 | 2246599 | -0,4169 | -0,7338 | -- | -- | | -- |
| Q025036 | MAP3K7IP2 | 23118 | ENSG00000055208 | 3751850 | 1722206 | 0,0107 | -0,9566 | -- | DOWN | | -- |
| Q006532 | MAP3K8 | 1326 | ENSG00000107968 | 4185272 | 2663579 | 0,2157 | -0,4829 | -- | -- | | -- |
| Q024608 | MAP3K8 | 1326 | ENSG00000107968 | 4019330 | 3627821 | 0,1649 | 0,0163 | -- | -- | | -- |
| Q006637 | MAP3K9 | 4293 | ENSG00000006432 | 4889787 | 4012034 | 0,2835 | 0,0996 | -- | -- | | -- |
| Q024713 | MAP3K9 | 4293 | ENSG00000006432 | 4844321 | 4169088 | 0,1487 | 0,1569 | -- | -- | | -- |
| Q006938 | MAP4K1 | 11184 | ENSG00000104814 | 2378995 | 2080856 | -0,7111 | -0,9767 | DOWN | DOWN | | DOWN-HIT |
| Q025014 | MAP4K1 | 11184 | ENSG00000104814 | 3627811 | 3276607 | -0,0514 | -0,2426 | -- | -- | | -- |
| Q006763 | MAP4K2 | 5871 | ENSG00000168067 | 3498500 | 3377477 | 0,2125 | 0,1192 | -- | -- | | -- |
| Q024839 | MAP4K2 | 5871 | ENSG00000168067 | 1696499 | 1627254 | -0,6124 | -0,6237 | DOWN | -- | | -- |
| Q006830 | MAP4K3 | 8491 | ENSG00000011566 | 2853746 | 3404889 | -0,0947 | 0,0072 | -- | -- | | -- |
| Q024906 | MAP4K3 | 8491 | ENSG00000011566 | 2465856 | 1949331 | -0,2124 | -0,7325 | -- | -- | | -- |
| Q006882 | MAP4K4 | 9448 | ENSG00000071054 | 5086792 | 3233273 | 0,2919 | -0,2188 | -- | -- | | -- |
| Q024958 | MAP4K4 | 9448 | ENSG00000071054 | 2239859 | 3307710 | -0,8343 | -0,1243 | DOWN | -- | | -- |
| Q006937 | MAP4K5 | 11183 | ENSG00000012983 | 3781021 | 2983537 | 0,0398 | -0,3493 | -- | -- | | -- |
| Q025013 | MAP4K5 | 11183 | ENSG00000012983 | 2942319 | 2047265 | -0,2495 | -0,9058 | -- | DOWN | | -- |
| Q006737 | MAPK1 | 5594 | ENSG00000100030 | 4782380 | 4302690 | 0,2963 | 0,2313 | -- | -- | | -- |
| Q024813 | MAPK1 | 5594 | ENSG00000100030 | 3953121 | 3650573 | 0,0391 | 0,0021 | -- | -- | | -- |
| Q006745 | MAPK10 | 5602 | ENSG00000109339 | 5096351 | 4414788 | 0,4015 | 0,2524 | -- | -- | | -- |
| Q024821 | MAPK10 | 5602 | ENSG00000109339 | 3204315 | 3041171 | -0,2399 | -0,2765 | -- | -- | | -- |
| Q006743 | MAPK11 | 5600 | ENSG00000185386 | 4489542 | 4866214 | 0,2235 | 0,3634 | -- | -- | | -- |
| Q024819 | MAPK11 | 5600 | ENSG00000185386 | 4349696 | 3617374 | 0,1059 | -0,0620 | -- | -- | | -- |
| Q006776 | MAPK12 | 6300 | ENSG00000188130 | 3824206 | 4306821 | 0,0309 | 0,1269 | -- | -- | | -- |
| Q024852 | MAPK12 | 6300 | ENSG00000188130 | 3321135 | 3267494 | -0,1940 | -0,2816 | -- | -- | | -- |
| Q006746 | MAPK13 | 5603 | ENSG00000156711 | 4601872 | 4298928 | 0,3592 | 0,3063 | -- | -- | | -- |
| Q024822 | MAPK13 | 5603 | ENSG00000156711 | 2110889 | 3122643 | -0,6340 | -0,0935 | DOWN | -- | | -- |
| Q006533 | MAPK14 | 1432 | ENSG00000112062 | 2147653 | 2174296 | -0,5024 | -0,5702 | -- | -- | | -- |
| Q024609 | MAPK14 | 1432 | ENSG00000112062 | 3140004 | 2240220 | -0,0592 | -0,4569 | -- | -- | | -- |
| Q006738 | MAPK3 | 5595 | ENSG00000102882 | 4551154 | 3678825 | 0,3399 | 0,1101 | -- | -- | | -- |
| Q024814 | MAPK3 | 5595 | ENSG00000102882 | 3448534 | 2981919 | -0,0159 | -0,1470 | -- | -- | | -- |
| Q006739 | MAPK4 | 5596 | ENSG00000141639 | 3372741 | 3207293 | 0,1588 | 0,0836 | -- | -- | | -- |
| Q024815 | MAPK4 | 5596 | ENSG00000141639 | 2160696 | 1980405 | -0,3655 | -0,4063 | -- | -- | | -- |
| Q006740 | MAPK6 | 5597 | ENSG00000069956 | 3837342 | 4427662 | 0,2429 | 0,3738 | -- | -- | | -- |
| Q024816 | MAPK6 | 5597 | ENSG00000069956 | 3724027 | 2335475 | 0,2439 | -0,3255 | -- | -- | | -- |
| Q006741 | MAPK7 | 5598 | ENSG00000166484 | 4693174 | 4721712 | 0,3876 | 0,3814 | -- | -- | | -- |
| Q024817 | MAPK7 | 5598 | ENSG00000166484 | 2246615 | 1699130 | -0,6211 | -0,9034 | DOWN | DOWN | | DOWN-HIT |
| Q006742 | MAPK8 | 5599 | ENSG00000107643 | 4638196 | 5024278 | 0,3049 | 0,4276 | -- | -- | | -- |
| Q024818 | MAPK8 | 5599 | ENSG00000107643 | 2615126 | 2612408 | -0,6094 | -0,4762 | DOWN | -- | | -- |
| Q006888 | MAPK8IP1 | 9479 | ENSG00000121653 | 1930245 | 2099096 | -0,5974 | -0,6690 | DOWN | -- | | -- |
| Q024964 | MAPK8IP1 | 9479 | ENSG00000121653 | 2878897 | 2807863 | -0,0692 | -0,2278 | -- | -- | | -- |
| Q006968 | MAPK8IP2 | 23542 | ENSG00000008735 | 3446799 | 3066382 | -0,1990 | -0,2356 | -- | -- | | -- |
| Q025044 | MAPK8IP2 | 23542 | ENSG00000008735 | 3929927 | 3429244 | 0,1169 | 0,0441 | -- | -- | | -- |
| Q006962 | MAPK8IP3 | 23162 | ENSG00000138834 | 2544337 | 2286569 | -0,5000 | -0,6884 | -- | -- | | -- |
| Q025038 | MAPK8IP3 | 23162 | ENSG00000138834 | 2864812 | 2712343 | -0,2295 | -0,3368 | -- | -- | | -- |
| Q006744 | MAPK9 | 5601 | ENSG00000050748 | 4799453 | 4642761 | 0,2928 | 0,2914 | -- | -- | | -- |
| Q024820 | MAPK9 | 5601 | ENSG00000050748 | 3044038 | 2940567 | -0,4264 | -0,3856 | -- | -- | | -- |
| Q007076 | MAPKAP1 | 79109 | ENSG00000119487 | 2934452 | 2600728 | -0,1649 | -0,4012 | -- | -- | | -- |
| Q025152 | MAPKAP1 | 79109 | ENSG00000119487 | 2786603 | 2087506 | -0,1864 | -0,7337 | -- | -- | | -- |
| Q006877 | MAPKAPK2 | 9261 | ENSG00000162889 | 2389167 | 1946442 | -1,0843 | -1,0308 | DOWN | DOWN | | DOWN-HIT |
| Q024953 | MAPKAPK2 | 9261 | ENSG00000162889 | 3694235 | 3065715 | -0,1548 | -0,2382 | -- | -- | | -- |
| Q006819 | MAPKAPK3 | 7867 | ENSG00000114738 | 3877673 | 2580765 | -0,0863 | -0,5175 | -- | -- | | -- |
| Q024895 | MAPKAPK3 | 7867 | ENSG00000114738 | 2961681 | 2330348 | -0,3817 | -0,5589 | -- | -- | | -- |
| Q006838 | MAPKAPK5 | 8550 | ENSG00000089022 | 2624127 | 2856597 | -0,1975 | -0,2299 | -- | -- | | -- |
| Q024914 | MAPKAPK5 | 8550 | ENSG00000089022 | 3281443 | 3738630 | 0,1063 | 0,1183 | -- | -- | | -- |
| Q006951 | MAPKBP1 | 23005 | ENSG00000137802 | 3992928 | 3245919 | 0,1095 | -0,1358 | -- | -- | | -- |
| Q025027 | MAPKBP1 | 23005 | ENSG00000137802 | 3809403 | 2434856 | 0,1685 | -0,4288 | -- | -- | | -- |
| Q024703 | MARCKS | 4082 | ENSG00000155130 | 3396816 | 2165569 | -0,2727 | -0,7607 | -- | -- | | -- |
| Q006627 | MARCKS | 4082 | ENSG00000155130 | 2423021 | 1768817 | -0,6039 | -0,8943 | DOWN | DOWN | | DOWN-HIT |
| Q006629 | MARK1 | 4139 | ENSG00000116141 | 3420390 | 2419700 | -0,2368 | -0,5903 | -- | -- | | -- |
| Q024705 | MARK1 | 4139 | ENSG00000116141 | 3867299 | 3223069 | -0,2684 | -0,3007 | -- | -- | | -- |
| Q006559 | MARK2 | 2011 | ENSG00000072518 | 2450544 | 2141266 | -0,3634 | -0,4478 | -- | -- | | -- |
| Q024635 | MARK2 | 2011 | ENSG00000072518 | 2285097 | 2062081 | -0,5067 | -0,5300 | -- | -- | | -- |
| Q006630 | MARK3 | 4140 | ENSG00000075413 | 4316217 | 2218457 | 0,1497 | -0,6628 | -- | -- | | -- |
| Q024706 | MARK3 | 4140 | ENSG00000075413 | 4504061 | 3685109 | 0,0312 | -0,0894 | -- | -- | | -- |
| Q007061 | MARK4 | 57787 | ENSG00000007047 | 3967146 | 2606230 | 0,0592 | -0,4738 | -- | -- | | -- |
| Q025137 | MARK4 | 57787 | ENSG00000007047 | 3860154 | 3387790 | 0,0792 | -0,1220 | -- | -- | | -- |
| Q006961 | MAST2 | 23139 | ENSG00000086015 | 2316473 | 1951369 | -0,5237 | -0,8073 | -- | -- | | -- |
| Q025037 | MAST2 | 23139 | ENSG00000086015 | 2484533 | 1475777 | -0,3320 | -0,9438 | -- | DOWN | | -- |
| Q006955 | MAST3 | 23031 | ENSG00000099308 | 4436362 | 3614041 | 0,0818 | -0,1221 | -- | -- | | -- |
| Q025031 | MAST3 | 23031 | ENSG00000099308 | 3449720 | 2224374 | -0,1734 | -0,7298 | -- | -- | | -- |
| Q006964 | MAST4 | 23227 | ENSG00000069020 | 4043355 | 3004074 | -0,1329 | -0,3548 | -- | -- | | -- |
| Q025040 | MAST4 | 23227 | ENSG00000069020 | 4820027 | 3341741 | 0,2834 | -0,1248 | -- | -- | | -- |
| Q007103 | MASTL | 84930 | ENSG00000120539 | 3805577 | 3541141 | -0,0684 | -0,1595 | -- | -- | | -- |
| Q025179 | MASTL | 84930 | ENSG00000120539 | 3913700 | 4043255 | 0,0172 | 0,0652 | -- | -- | | -- |
| Q006631 | MATK | 4145 | ENSG00000007264 | 3890233 | 2329824 | 0,0684 | -0,4988 | -- | -- | | -- |
| Q024707 | MATK | 4145 | ENSG00000007264 | 4070929 | 2813211 | -0,0382 | -0,4516 | -- | -- | | -- |
| Q006898 | MELK | 9833 | ENSG00000165304 | 3063000 | 2413735 | -0,3669 | -0,6014 | -- | -- | | -- |
| Q024974 | MELK | 9833 | ENSG00000165304 | 2700779 | 2100032 | -0,4966 | -0,8673 | -- | -- | | -- |
| Q006917 | MERTK | 10461 | ENSG00000153208 | 3516087 | 2998024 | -0,1214 | -0,2745 | -- | -- | | -- |
| Q024993 | MERTK | 10461 | ENSG00000153208 | 3692879 | 3016580 | -0,0060 | -0,2432 | -- | -- | | -- |
| Q006636 | MET | 4233 | ENSG00000105976 | 5009168 | 3887601 | 0,3540 | 0,0701 | -- | -- | | -- |
| Q024712 | MET | 4233 | ENSG00000105976 | 3591808 | 2152153 | -0,2957 | -0,7769 | -- | -- | | -- |
| Q007151 | MGC40579 | 256356 | ENSG00000175066 | 2930061 | 1835636 | -0,2030 | -0,9144 | -- | DOWN | | -- |
| Q025227 | MGC40579 | 256356 | ENSG00000175066 | 2480511 | 2601938 | -0,4754 | -0,4030 | -- | -- | | -- |
| Q007095 | MGC4796 | 83931 | ENSG00000196182 | 2187486 | 1608147 | -0,8236 | -1,2629 | DOWN | DOWN | | DOWN-HIT |
| Q025171 | MGC4796 | 83931 | ENSG00000196182 | 2627102 | 2212295 | -0,5726 | -0,8296 | DOWN | -- | | -- |
| Q007158 | MGC75495 | 341676 | NA | 2559033 | 2678189 | -0,0999 | -0,2773 | -- | -- | | -- |
| Q025234 | MGC75495 | 341676 | NA | 3261441 | 3504413 | 0,1509 | 0,0898 | -- | -- | | -- |
| Q007004 | MINK1 | 50488 | ENSG00000141503 | 2401048 | 2347646 | -0,6384 | -0,6447 | DOWN | -- | | -- |
| Q025080 | MINK1 | 50488 | ENSG00000141503 | 3635374 | 3572361 | -0,0664 | -0,1067 | -- | -- | | -- |
| Q006842 | MKNK1 | 8569 | ENSG00000079277 | 3886582 | 3170697 | 0,0257 | -0,2037 | -- | -- | | -- |
| Q024918 | MKNK1 | 8569 | ENSG00000079277 | 3029726 | 2894533 | -0,3562 | -0,4063 | -- | -- | | -- |
| Q006598 | MKNK2 | 2872 | ENSG00000099875 | 3023946 | 3409582 | -0,2564 | -0,0894 | -- | -- | | -- |
| Q024674 | MKNK2 | 2872 | ENSG00000099875 | 3221270 | 2934420 | -0,1173 | -0,2504 | -- | -- | | -- |
| Q006640 | MOS | 4342 | ENSG00000172680 | 3761019 | 2215599 | 0,1013 | -0,4779 | -- | -- | | -- |
| Q024716 | MOS | 4342 | ENSG00000172680 | 4677882 | 4111384 | 0,3375 | 0,2555 | -- | -- | | -- |
| Q006387 | M-RIP | 23164 | ENSG00000133030 | 2871014 | 2524483 | -0,0305 | -0,2299 | -- | -- | | -- |
| Q024463 | M-RIP | 23164 | ENSG00000133030 | 1864508 | 1848787 | -0,5403 | -0,5373 | DOWN | -- | | -- |
| Q006641 | MST1R | 4486 | ENSG00000164078 | 2968457 | 2427072 | 0,0101 | -0,2092 | -- | -- | | -- |
| Q024717 | MST1R | 4486 | ENSG00000164078 | 2862140 | 2357527 | -0,1217 | -0,2889 | -- | -- | | -- |
| Q007018 | MST4 | 51765 | ENSG00000134602 | 3912248 | 3983279 | 0,1595 | 0,1328 | -- | -- | | -- |
| Q025094 | MST4 | 51765 | ENSG00000134602 | 2734230 | 3106047 | -0,2503 | -0,2245 | -- | -- | | -- |
| Q006275 | MTM1 | 4534 | ENSG00000171100 | 3497777 | 3417759 | 0,0204 | -0,0277 | -- | -- | | -- |
| Q024351 | MTM1 | 4534 | ENSG00000171100 | 2900654 | 2871782 | -0,2736 | -0,2494 | -- | -- | | -- |
| Q006367 | MTMR2 | 8898 | ENSG00000087053 | 4927042 | 4810220 | 0,3509 | 0,4038 | -- | -- | | -- |
| Q024443 | MTMR2 | 8898 | ENSG00000087053 | 3996088 | 2498994 | -0,0344 | -0,5434 | -- | -- | | -- |
| Q006366 | MTMR3 | 8897 | ENSG00000100330 | 4510990 | 4862188 | 0,2370 | 0,4129 | -- | -- | | -- |
| Q024442 | MTMR3 | 8897 | ENSG00000100330 | 4430403 | 3812697 | 0,1413 | 0,0698 | -- | -- | | -- |
| Q006371 | MTMR4 | 9110 | ENSG00000108389 | 2936752 | 2434286 | 0,0160 | -0,1794 | -- | -- | | -- |
| Q024447 | MTMR4 | 9110 | ENSG00000108389 | 2034682 | 2053035 | -0,4076 | -0,3447 | -- | -- | | -- |
| Q006370 | MTMR6 | 9107 | ENSG00000139505 | 2516726 | 2088063 | -0,3778 | -0,5474 | -- | -- | | -- |
| Q024446 | MTMR6 | 9107 | ENSG00000139505 | 3989276 | 3554186 | 0,2596 | 0,1674 | -- | -- | | -- |
| Q006411 | MTMR8 | 55613 | ENSG00000102043 | 2628159 | 2247132 | -0,1951 | -0,4546 | -- | -- | | -- |
| Q024487 | MTMR8 | 55613 | ENSG00000102043 | 3323345 | 1927057 | 0,0213 | -0,5803 | -- | -- | | -- |
| Q006423 | MTMR9 | 66036 | ENSG00000104643 | 4876203 | 4195651 | 0,3611 | 0,2574 | -- | -- | | -- |
| Q024499 | MTMR9 | 66036 | ENSG00000104643 | 4938162 | 4288574 | 0,3650 | 0,2952 | -- | -- | | -- |
| Q006642 | MUSK | 4593 | ENSG00000030304 | 3236271 | 2875671 | -0,0013 | -0,1625 | -- | -- | | -- |
| Q024718 | MUSK | 4593 | ENSG00000030304 | 3483933 | 3397611 | 0,1008 | 0,1013 | -- | -- | | -- |
| Q006643 | MVK | 4598 | ENSG00000110921 | 2934686 | 2727961 | -0,3459 | -0,3863 | -- | -- | | -- |
| Q024719 | MVK | 4598 | ENSG00000110921 | 3513010 | 2629015 | -0,1154 | -0,3416 | -- | -- | | -- |
| Q006644 | MYLK | 4638 | ENSG00000065534 | 4051152 | 3328238 | 0,0146 | -0,1868 | -- | -- | | -- |
| Q024720 | MYLK | 4638 | ENSG00000065534 | 4082575 | 2803406 | -0,0413 | -0,3483 | -- | -- | | -- |
| Q007105 | MYLK2 | 85366 | ENSG00000101306 | 4228019 | 4020994 | 0,1228 | 0,0982 | -- | -- | | -- |
| Q025181 | MYLK2 | 85366 | ENSG00000101306 | 3088609 | 2904359 | -0,2505 | -0,3379 | -- | -- | | -- |
| Q007044 | NAGK | 55577 | ENSG00000124357 | 2465373 | 2435462 | -0,5168 | -0,5675 | -- | -- | | -- |
| Q025120 | NAGK | 55577 | ENSG00000124357 | 2954058 | 2238082 | -0,1704 | -0,5882 | -- | -- | | -- |
| Q006645 | NEK1 | 4750 | ENSG00000137601 | 4474436 | 3231003 | 0,1277 | -0,2555 | -- | -- | | -- |
| Q024721 | NEK1 | 4750 | ENSG00000137601 | 4261825 | 3419376 | -0,0342 | -0,1045 | -- | -- | | -- |
| Q007081 | NEK11 | 79858 | ENSG00000114670 | 2638701 | 2366150 | -0,5040 | -0,5883 | -- | -- | | -- |
| Q025157 | NEK11 | 79858 | ENSG00000114670 | 3193202 | 2587528 | -0,1672 | -0,3981 | -- | -- | | -- |
| Q006646 | NEK2 | 4751 | ENSG00000117650 | 4509413 | 3278028 | 0,1505 | -0,1967 | -- | -- | | -- |
| Q024722 | NEK2 | 4751 | ENSG00000117650 | 4810955 | 2988239 | 0,2007 | -0,3163 | -- | -- | | -- |
| Q006647 | NEK3 | 4752 | ENSG00000136098 | 3884773 | 2606023 | -0,0170 | -0,4424 | -- | -- | | -- |
| Q024723 | NEK3 | 4752 | ENSG00000136098 | 2199431 | 1880187 | -0,9368 | -0,9351 | DOWN | DOWN | | DOWN-HIT |
| Q006785 | NEK4 | 6787 | ENSG00000114904 | 3647888 | 4426996 | -0,0234 | 0,1700 | -- | -- | | -- |
| Q024861 | NEK4 | 6787 | ENSG00000114904 | 3829322 | 2126436 | 0,0164 | -0,9725 | -- | DOWN | | -- |
| Q006928 | NEK6 | 10783 | ENSG00000119408 | 2614692 | 2417697 | -0,2407 | -0,4870 | -- | -- | | -- |
| Q025004 | NEK6 | 10783 | ENSG00000119408 | 3045544 | 3368779 | -0,0085 | -0,0538 | -- | -- | | -- |
| Q007130 | NEK7 | 140609 | ENSG00000151414 | 3299566 | 1913450 | -0,4185 | -0,8581 | -- | -- | | -- |
| Q025206 | NEK7 | 140609 | ENSG00000151414 | 4511427 | 3431964 | 0,1435 | -0,0185 | -- | -- | | -- |
| Q007156 | NEK8 | 284086 | ENSG00000160602 | 2872397 | 2808728 | -0,2842 | -0,2373 | -- | -- | | -- |
| Q025232 | NEK8 | 284086 | ENSG00000160602 | 3598747 | 3164239 | 0,0814 | -0,0269 | -- | -- | | -- |
| Q007110 | NEK9 | 91754 | ENSG00000119638 | 4021894 | 2767486 | -0,0261 | -0,6781 | -- | -- | | -- |
| Q025186 | NEK9 | 91754 | ENSG00000119638 | 4339283 | 2911598 | 0,1716 | -0,5060 | -- | -- | | -- |
| Q007065 | NJMU-R1 | 64149 | ENSG00000108666 | 3222837 | 2654583 | -0,3048 | -0,4545 | -- | -- | | -- |
| Q025141 | NJMU-R1 | 64149 | ENSG00000108666 | 4678933 | 4129638 | 0,3434 | 0,2611 | -- | -- | | -- |
| Q007015 | NLK | 51701 | ENSG00000087095 | 2855589 | 1951105 | -0,3778 | -0,8491 | -- | -- | | -- |
| Q025091 | NLK | 51701 | ENSG00000087095 | 3515399 | 2832770 | -0,0439 | -0,3148 | -- | -- | | -- |
| Q006648 | NME1 | 4830 | ENSG00000011052 | 3877363 | 2754092 | 0,1210 | -0,2377 | -- | -- | | -- |
| Q024724 | NME1 | 4830 | ENSG00000011052 | 4588984 | 2686571 | 0,3375 | -0,2862 | -- | -- | | -- |
| Q006649 | NME2 | 4831 | ENSG00000121054 | 2587294 | 1961018 | -0,1551 | -0,4548 | -- | -- | | -- |
| Q024725 | NME2 | 4831 | ENSG00000121054 | 3400844 | 2577326 | 0,1175 | -0,1465 | -- | -- | | -- |
| Q006650 | NME3 | 4832 | ENSG00000103024 | 2445074 | 2729119 | -0,3293 | -0,2407 | -- | -- | | -- |
| Q024726 | NME3 | 4832 | ENSG00000103024 | 1952335 | 2283094 | -0,5309 | -0,3536 | -- | -- | | -- |
| Q006651 | NME4 | 4833 | ENSG00000103200 | 3532195 | 2538970 | -0,0483 | -0,4934 | -- | -- | | -- |
| Q024727 | NME4 | 4833 | ENSG00000103200 | 2998684 | 2734821 | -0,2894 | -0,2760 | -- | -- | | -- |
| Q006821 | NME5 | 8382 | ENSG00000112981 | 3078921 | 2069127 | -0,1824 | -0,6419 | -- | -- | | -- |
| Q024897 | NME5 | 8382 | ENSG00000112981 | 2772970 | 1863793 | -0,1873 | -0,5523 | -- | -- | | -- |
| Q006909 | NME6 | 10201 | ENSG00000172113 | 3105340 | 2801620 | -0,3361 | -0,3849 | -- | -- | | -- |
| Q024985 | NME6 | 10201 | ENSG00000172113 | 3736614 | 3758260 | 0,0022 | 0,0804 | -- | -- | | -- |
| Q006997 | NME7 | 29922 | ENSG00000143156 | 2853777 | 3283497 | -0,4371 | -0,1607 | -- | -- | | -- |
| Q025073 | NME7 | 29922 | ENSG00000143156 | 4039434 | 3177735 | 0,0537 | -0,3084 | -- | -- | | -- |
| Q006999 | NRBP | 29959 | ENSG00000115216 | 3779439 | 3230353 | -0,0115 | -0,1603 | -- | -- | | -- |
| Q025075 | NRBP | 29959 | ENSG00000115216 | 1641889 | 2257107 | -1,0817 | -0,5702 | DOWN | -- | | -- |
| Q006652 | NRGN | 4900 | ENSG00000154146 | 4142258 | 3930801 | 0,0810 | 0,0598 | -- | -- | | -- |
| Q024728 | NRGN | 4900 | ENSG00000154146 | 4212318 | 3122547 | 0,0547 | -0,1952 | -- | -- | | -- |
| Q007146 | NRK | 203447 | ENSG00000123572 | 4436164 | 2742037 | 0,1517 | -0,3553 | -- | -- | | -- |
| Q025222 | NRK | 203447 | ENSG00000123572 | 4846487 | 4211235 | 0,2824 | 0,2741 | -- | -- | | -- |
| Q006653 | NTRK1 | 4914 | ENSG00000198400 | 2394392 | 2232859 | -0,8627 | -0,8333 | DOWN | -- | | -- |
| Q024729 | NTRK1 | 4914 | ENSG00000198400 | 3200197 | 2749327 | -0,4670 | -0,4325 | -- | -- | | -- |
| Q006654 | NTRK2 | 4915 | ENSG00000148053 | 3725212 | 2121358 | -0,1437 | -0,8794 | -- | -- | | -- |
| Q024730 | NTRK2 | 4915 | ENSG00000148053 | 4115559 | 3647278 | -0,0119 | -0,0320 | -- | -- | | -- |
| Q006655 | NTRK3 | 4916 | ENSG00000140538 | 3624795 | 2806109 | -0,1327 | -0,3730 | -- | -- | | -- |
| Q024731 | NTRK3 | 4916 | ENSG00000140538 | 2827574 | 2652676 | -0,4831 | -0,4500 | -- | -- | | -- |
| Q007066 | NUCKS | 64710 | ENSG00000069275 | 4239559 | 3036924 | 0,1902 | -0,1898 | -- | -- | | -- |
| Q025142 | NUCKS | 64710 | ENSG00000069275 | 2633752 | 2191787 | -0,3280 | -0,4477 | -- | -- | | -- |
| Q007108 | NYD-SP25 | 89882 | ENSG00000170777 | 2928591 | 2544401 | -0,2947 | -0,6131 | -- | -- | | -- |
| Q007107 | NYD-SP25 | 89882 | ENSG00000170777 | 2478773 | 2124010 | -0,3610 | -0,5125 | -- | -- | | -- |
| Q025184 | NYD-SP25 | 89882 | ENSG00000170777 | 3489537 | 3360183 | 0,0126 | -0,1813 | -- | -- | | -- |
| Q025183 | NYD-SP25 | 89882 | ENSG00000170777 | 3467728 | 2438827 | 0,1437 | -0,2495 | -- | -- | | -- |
| Q006902 | OXSR1 | 9943 | ENSG00000172939 | 3765719 | 3289103 | -0,0315 | -0,1210 | -- | -- | | -- |
| Q024978 | OXSR1 | 9943 | ENSG00000172939 | 2257228 | 1955581 | -0,6155 | -0,6634 | DOWN | -- | | -- |
| Q007000 | PACSIN1 | 29993 | ENSG00000124507 | 3171572 | 3441256 | -0,1671 | -0,0026 | -- | -- | | -- |
| Q025076 | PACSIN1 | 29993 | ENSG00000124507 | 2303211 | 2224599 | -0,4671 | -0,4351 | -- | -- | | -- |
| Q006944 | PACSIN2 | 11252 | ENSG00000100266 | 3179343 | 2608295 | -0,1015 | -0,4951 | -- | -- | | -- |
| Q025020 | PACSIN2 | 11252 | ENSG00000100266 | 2616661 | 2590237 | -0,2781 | -0,5360 | -- | -- | | -- |
| Q006995 | PACSIN3 | 29763 | ENSG00000165912 | 3861372 | 4256440 | 0,0649 | 0,2284 | -- | -- | | -- |
| Q025071 | PACSIN3 | 29763 | ENSG00000165912 | 3055838 | 3235173 | -0,2372 | -0,2209 | -- | -- | | -- |
| Q006659 | PAK1 | 5058 | ENSG00000149269 | 2961387 | 1950771 | -0,2643 | -0,8674 | -- | -- | | -- |
| Q024735 | PAK1 | 5058 | ENSG00000149269 | 3118442 | 2701235 | -0,1910 | -0,2991 | -- | -- | | -- |
| Q024736 | PAK2 | 5062 | ENSG00000180370 | 4266374 | 3054750 | 0,1165 | -0,2434 | -- | -- | | -- |
| Q006660 | PAK2 | 5062 | ENSG00000180370 | 2322160 | 1753469 | -0,7490 | -1,1514 | DOWN | DOWN | | DOWN-HIT |
| Q006661 | PAK3 | 5063 | ENSG00000077264 | 4629101 | 1790726 | 0,2537 | -1,1718 | -- | DOWN | | -- |
| Q024737 | PAK3 | 5063 | ENSG00000077264 | 4372006 | 3743552 | 0,0946 | -0,0028 | -- | -- | | -- |
| Q006914 | PAK4 | 10298 | ENSG00000130669 | 2904325 | 3651450 | -0,3562 | -0,0025 | -- | -- | | -- |
| Q024990 | PAK4 | 10298 | ENSG00000130669 | 4763908 | 4371877 | 0,3525 | 0,2057 | -- | -- | | -- |
| Q007051 | PAK6 | 56924 | ENSG00000137843 | 3143671 | 2786274 | -0,0989 | -0,2490 | -- | -- | | -- |
| Q025127 | PAK6 | 56924 | ENSG00000137843 | 3700557 | 2810667 | 0,2158 | -0,0656 | -- | -- | | -- |
| Q007055 | PAK7 | 57144 | ENSG00000101349 | 3755965 | 2927649 | -0,2185 | -0,3383 | -- | -- | | -- |
| Q025131 | PAK7 | 57144 | ENSG00000101349 | 3855671 | 3565304 | -0,0621 | -0,0267 | -- | -- | | -- |
| Q007020 | PANK1 | 53354 | ENSG00000152782 | 4163650 | 3162708 | 0,1051 | -0,2906 | -- | -- | | -- |
| Q025096 | PANK1 | 53354 | ENSG00000152782 | 2832046 | 2721512 | -0,4371 | -0,5222 | -- | -- | | -- |
| Q007083 | PANK2 | 80025 | ENSG00000125779 | 2216127 | 2140834 | -0,4108 | -0,4557 | -- | -- | | -- |
| Q025159 | PANK2 | 80025 | ENSG00000125779 | 2163736 | 2064842 | -0,3316 | -0,3320 | -- | -- | | -- |
| Q007077 | PANK3 | 79646 | ENSG00000120137 | 4561828 | 4717496 | 0,3027 | 0,3744 | -- | -- | | -- |
| Q025153 | PANK3 | 79646 | ENSG00000120137 | 2302650 | 2489526 | -0,6245 | -0,6096 | DOWN | -- | | -- |
| Q007031 | PANK4 | 55229 | ENSG00000157881 | 3972731 | 3270972 | 0,0044 | -0,2517 | -- | -- | | -- |
| Q025107 | PANK4 | 55229 | ENSG00000157881 | 3181940 | 2562960 | -0,2183 | -0,5561 | -- | -- | | -- |
| Q006963 | PASK | 23178 | ENSG00000115687 | 4163445 | 3669608 | 0,0183 | -0,0442 | -- | -- | | -- |
| Q025039 | PASK | 23178 | ENSG00000115687 | 5052999 | 3502447 | 0,4241 | -0,0403 | -- | -- | | -- |
| Q007047 | PBK | 55872 | ENSG00000168078 | 4122270 | 2471305 | -0,1302 | -0,6151 | -- | -- | | -- |
| Q025123 | PBK | 55872 | ENSG00000168078 | 3893281 | 2587507 | -0,0734 | -0,4674 | -- | -- | | -- |
| Q006662 | PCK1 | 5105 | ENSG00000124253 | 3874638 | 3580208 | -0,0515 | -0,0954 | -- | -- | | -- |
| Q024738 | PCK1 | 5105 | ENSG00000124253 | 3933841 | 3050331 | -0,0438 | -0,3208 | -- | -- | | -- |
| Q006663 | PCTK1 | 5127 | ENSG00000102225 | 3552139 | 3433892 | -0,1573 | -0,1064 | -- | -- | | -- |
| Q024739 | PCTK1 | 5127 | ENSG00000102225 | 3898235 | 3492624 | 0,0332 | -0,0688 | -- | -- | | -- |
| Q006664 | PCTK2 | 5128 | ENSG00000059758 | 4619213 | 3790765 | 0,3624 | 0,1421 | -- | -- | | -- |
| Q024740 | PCTK2 | 5128 | ENSG00000059758 | 3936527 | 3656646 | 0,1718 | 0,1189 | -- | -- | | -- |
| Q006665 | PCTK3 | 5129 | ENSG00000117266 | 2194661 | 2049444 | -0,3261 | -0,4381 | -- | -- | | -- |
| Q024741 | PCTK3 | 5129 | ENSG00000117266 | 1821046 | 1881229 | -0,5369 | -0,4573 | DOWN | -- | | -- |
| Q006666 | PDGFRA | 5156 | ENSG00000134853 | 3313566 | 4025657 | 0,0551 | 0,2356 | -- | -- | | -- |
| Q024742 | PDGFRA | 5156 | ENSG00000134853 | 2598489 | 2787316 | -0,1799 | -0,1237 | -- | -- | | -- |
| Q006667 | PDGFRB | 5159 | ENSG00000113721 | 3845944 | 3236377 | 0,1126 | -0,1664 | -- | -- | | -- |
| Q024743 | PDGFRB | 5159 | ENSG00000113721 | 3351725 | 2783561 | -0,0729 | -0,2722 | -- | -- | | -- |
| Q007134 | PDIK1L | 149420 | ENSG00000175087 | 1789418 | 2056676 | -0,5225 | -0,5233 | -- | -- | | -- |
| Q025210 | PDIK1L | 149420 | ENSG00000175087 | 3308389 | 3508116 | 0,1364 | 0,1355 | -- | -- | | -- |
| Q006668 | PDK1 | 5163 | ENSG00000152256 | 4352690 | 2545139 | 0,2212 | -0,5893 | -- | -- | | -- |
| Q024744 | PDK1 | 5163 | ENSG00000152256 | 4608080 | 4110863 | 0,2629 | 0,1727 | -- | -- | | -- |
| Q006669 | PDK2 | 5164 | ENSG00000005882 | 3827891 | 3626314 | -0,0028 | -0,0786 | -- | -- | | -- |
| Q024745 | PDK2 | 5164 | ENSG00000005882 | 3783993 | 3958800 | -0,0873 | 0,0711 | -- | -- | | -- |
| Q006670 | PDK3 | 5165 | ENSG00000067992 | 4045470 | 3981511 | 0,0427 | 0,0584 | -- | -- | | -- |
| Q024746 | PDK3 | 5165 | ENSG00000067992 | 3682943 | 3679096 | -0,1075 | -0,0470 | -- | -- | | -- |
| Q006671 | PDK4 | 5166 | ENSG00000004799 | 4108368 | 3637760 | 0,0850 | -0,0369 | -- | -- | | -- |
| Q024747 | PDK4 | 5166 | ENSG00000004799 | 3182780 | 2699369 | -0,2333 | -0,4509 | -- | -- | | -- |
| Q006922 | PDLIM5 | 10611 | ENSG00000163110 | 3671369 | 3186121 | -0,0208 | -0,2067 | -- | -- | | -- |
| Q024998 | PDLIM5 | 10611 | ENSG00000163110 | 4145361 | 4103314 | 0,1580 | 0,1270 | -- | -- | | -- |
| Q006672 | PDPK1 | 5170 | ENSG00000140992 | 2967563 | 2583825 | -0,2469 | -0,3919 | -- | -- | | -- |
| Q024748 | PDPK1 | 5170 | ENSG00000140992 | 3257958 | 3052659 | -0,0655 | -0,1250 | -- | -- | | -- |
| Q006409 | PDPR | 55066 | ENSG00000090857 | 3402623 | 2875709 | -0,1424 | -0,5440 | -- | -- | | -- |
| Q024485 | PDPR | 55066 | ENSG00000090857 | 3839598 | 4198721 | 0,0180 | 0,0925 | -- | -- | | -- |
| Q006840 | PDXK | 8566 | ENSG00000160209 | 2904035 | 2709774 | -0,3482 | -0,4319 | -- | -- | | -- |
| Q024916 | PDXK | 8566 | ENSG00000160209 | 4374960 | 4211067 | 0,2332 | 0,1577 | -- | -- | | -- |
| Q006677 | PFKL | 5211 | ENSG00000141959 | 4570190 | 4299102 | 0,2861 | 0,1783 | -- | -- | | -- |
| Q024753 | PFKL | 5211 | ENSG00000141959 | 3217904 | 2539147 | -0,2706 | -0,5854 | -- | -- | | -- |
| Q006678 | PFKM | 5213 | ENSG00000152556 | 2984312 | 2449402 | -0,3787 | -0,6806 | -- | -- | | -- |
| Q024754 | PFKM | 5213 | ENSG00000152556 | 3029205 | 2942592 | -0,3431 | -0,3645 | -- | -- | | -- |
| Q006679 | PFKP | 5214 | ENSG00000067057 | 3855371 | 3065451 | 0,0287 | -0,2901 | -- | -- | | -- |
| Q024755 | PFKP | 5214 | ENSG00000067057 | 4375487 | 2946854 | 0,2530 | -0,3059 | -- | -- | | -- |
| Q006680 | PFTK1 | 5218 | ENSG00000058091 | 3194190 | 3366871 | -0,1203 | -0,0434 | -- | -- | | -- |
| Q024756 | PFTK1 | 5218 | ENSG00000058091 | 3495013 | 3468437 | 0,0423 | 0,0445 | -- | -- | | -- |
| Q006681 | PGK1 | 5230 | ENSG00000102144 | 2305175 | 1991879 | -0,2652 | -0,4972 | -- | -- | | -- |
| Q024757 | PGK1 | 5230 | ENSG00000102144 | 2361884 | 2463628 | -0,2607 | -0,1806 | -- | -- | | -- |
| Q006682 | PGK2 | 5232 | ENSG00000170950 | 4363220 | 3617316 | 0,3918 | 0,0436 | -- | -- | | -- |
| Q024758 | PGK2 | 5232 | ENSG00000170950 | 2393864 | 1984736 | -0,3134 | -0,6121 | -- | -- | | -- |
| Q006683 | PHKA1 | 5255 | ENSG00000067177 | 3500130 | 2952396 | 0,0009 | -0,3340 | -- | -- | | -- |
| Q024759 | PHKA1 | 5255 | ENSG00000067177 | 3219627 | 2917813 | -0,1073 | -0,2739 | -- | -- | | -- |
| Q006684 | PHKA2 | 5256 | ENSG00000044446 | 2328446 | 2241325 | -0,6396 | -0,8100 | DOWN | -- | | -- |
| Q024760 | PHKA2 | 5256 | ENSG00000044446 | 3095592 | 2967795 | -0,2480 | -0,3298 | -- | -- | | -- |
| Q006685 | PHKB | 5257 | ENSG00000102893 | 3720956 | 3360026 | 0,0031 | -0,2057 | -- | -- | | -- |
| Q024761 | PHKB | 5257 | ENSG00000102893 | 3961983 | 4102406 | 0,0767 | 0,1270 | -- | -- | | -- |
| Q006686 | PHKG1 | 5260 | ENSG00000164776 | 4296545 | 2929765 | 0,2000 | -0,4112 | -- | -- | | -- |
| Q024762 | PHKG1 | 5260 | ENSG00000164776 | 4378625 | 3542913 | 0,2296 | -0,0822 | -- | -- | | -- |
| Q006687 | PHKG2 | 5261 | ENSG00000156873 | 3964981 | 3748487 | 0,1034 | 0,0030 | -- | -- | | -- |
| Q024763 | PHKG2 | 5261 | ENSG00000156873 | 3019060 | 2483567 | -0,2484 | -0,5441 | -- | -- | | -- |
| Q007033 | PI4K2B | 55300 | ENSG00000038210 | 3304670 | 2765774 | -0,0406 | -0,2244 | -- | -- | | -- |
| Q025109 | PI4K2B | 55300 | ENSG00000038210 | 2598769 | 2202763 | -0,2191 | -0,4000 | -- | -- | | -- |
| Q007039 | PI4KII | 55361 | ENSG00000043822 | 4228040 | 2573776 | -0,1213 | -0,6197 | -- | -- | | -- |
| Q025115 | PI4KII | 55361 | ENSG00000043822 | 3972158 | 2727141 | -0,0741 | -0,4430 | -- | -- | | -- |
| Q007122 | PIK3AP1 | 118788 | ENSG00000155629 | 3906351 | 2195186 | -0,1458 | -0,7366 | -- | -- | | -- |
| Q025198 | PIK3AP1 | 118788 | ENSG00000155629 | 2561642 | 2301603 | -0,7993 | -0,6764 | DOWN | -- | | -- |
| Q006688 | PIK3C2A | 5286 | ENSG00000011405 | 3684698 | 2820614 | 0,0926 | -0,2983 | -- | -- | | -- |
| Q024764 | PIK3C2A | 5286 | ENSG00000011405 | 2868684 | 2306953 | -0,2114 | -0,4972 | -- | -- | | -- |
| Q006689 | PIK3C2B | 5287 | ENSG00000133056 | 2109393 | 1972204 | -0,3545 | -0,5179 | -- | -- | | -- |
| Q024765 | PIK3C2B | 5287 | ENSG00000133056 | 2556341 | 2334527 | -0,1880 | -0,2555 | -- | -- | | -- |
| Q006690 | PIK3C2G | 5288 | ENSG00000139144 | 3998034 | 3580616 | 0,2518 | -0,0213 | -- | -- | | -- |
| Q024766 | PIK3C2G | 5288 | ENSG00000139144 | 3021461 | 2310407 | -0,0975 | -0,5121 | -- | -- | | -- |
| Q006691 | PIK3C3 | 5289 | ENSG00000078142 | 4619320 | 3664860 | 0,3707 | -0,0622 | -- | -- | | -- |
| Q024767 | PIK3C3 | 5289 | ENSG00000078142 | 3787481 | 2955691 | 0,0772 | -0,3253 | -- | -- | | -- |
| Q006692 | PIK3CA | 5290 | ENSG00000121879 | 3478586 | 3225903 | -0,0712 | -0,2978 | -- | -- | | -- |
| Q024768 | PIK3CA | 5290 | ENSG00000121879 | 4348344 | 4035082 | 0,2196 | 0,0756 | -- | -- | | -- |
| Q006693 | PIK3CB | 5291 | ENSG00000051382 | 3785826 | 2946774 | 0,0310 | -0,4543 | -- | -- | | -- |
| Q024769 | PIK3CB | 5291 | ENSG00000051382 | 3747573 | 3926369 | -0,0113 | 0,0190 | -- | -- | | -- |
| Q006695 | PIK3CD | 5293 | ENSG00000171608 | 2795804 | 2085177 | -0,3971 | -0,9187 | -- | DOWN | | -- |
| Q024771 | PIK3CD | 5293 | ENSG00000171608 | 2458696 | 2451478 | -0,5551 | -0,6067 | DOWN | -- | | -- |
| Q006696 | PIK3CG | 5294 | ENSG00000105851 | 3815446 | 2559086 | 0,1382 | -0,4612 | -- | -- | | -- |
| Q024772 | PIK3CG | 5294 | ENSG00000105851 | 2996633 | 2006187 | -0,1840 | -0,7143 | -- | -- | | -- |
| Q006697 | PIK3R1 | 5295 | ENSG00000145675 | 2416434 | 2245170 | -0,2180 | -0,3956 | -- | -- | | -- |
| Q024773 | PIK3R1 | 5295 | ENSG00000145675 | 2544777 | 1715017 | -0,2307 | -0,6154 | -- | -- | | -- |
| Q006698 | PIK3R2 | 5296 | ENSG00000105647 | 3539954 | 3090734 | 0,0559 | -0,3070 | -- | -- | | -- |
| Q024774 | PIK3R2 | 5296 | ENSG00000105647 | 2726855 | 2465887 | -0,3100 | -0,5464 | -- | -- | | -- |
| Q006831 | PIK3R3 | 8503 | ENSG00000117461 | 4200950 | 3581008 | 0,2260 | 0,0045 | -- | -- | | -- |
| Q024907 | PIK3R3 | 8503 | ENSG00000117461 | 3377394 | 3221355 | -0,0286 | -0,1812 | -- | -- | | -- |
| Q007003 | PIK3R4 | 30849 | ENSG00000196455 | 3731020 | 2148214 | 0,0305 | -0,7443 | -- | -- | | -- |
| Q025079 | PIK3R4 | 30849 | ENSG00000196455 | 3302325 | 3226387 | -0,1217 | -0,2141 | -- | -- | | -- |
| Q006967 | PIK3R5 | 23533 | ENSG00000141506 | 3453398 | 2466616 | -0,3441 | -0,6175 | -- | -- | | -- |
| Q025043 | PIK3R5 | 23533 | ENSG00000141506 | 3389521 | 2136792 | -0,2235 | -0,6787 | -- | -- | | -- |
| Q006699 | PIK4CA | 5297 | ENSG00000133511 | 3267069 | 2236991 | -0,1383 | -0,9265 | -- | DOWN | | -- |
| Q024775 | PIK4CA | 5297 | ENSG00000133511 | 3276466 | 2666149 | -0,1833 | -0,6018 | -- | -- | | -- |
| Q006700 | PIK4CB | 5298 | ENSG00000143393 | 3336363 | 4188282 | -0,1533 | 0,0506 | -- | -- | | -- |
| Q024776 | PIK4CB | 5298 | ENSG00000143393 | 3780793 | 3474900 | -0,0271 | -0,2509 | -- | -- | | -- |
| Q006694 | PIM1 | 5292 | ENSG00000137193 | 3683752 | 3077223 | -0,0205 | -0,3752 | -- | -- | | -- |
| Q024770 | PIM1 | 5292 | ENSG00000137193 | 3924769 | 3174291 | 0,0633 | -0,2906 | -- | -- | | -- |
| Q006933 | PIM2 | 11040 | ENSG00000102096 | 2856543 | 1958371 | -0,4182 | -0,9081 | -- | DOWN | | -- |
| Q025009 | PIM2 | 11040 | ENSG00000102096 | 3666796 | 3315145 | -0,0266 | -0,1482 | -- | -- | | -- |
| Q007068 | PINK1 | 65018 | ENSG00000158828 | 3243369 | 2833157 | -0,0459 | -0,2845 | -- | -- | | -- |
| Q025144 | PINK1 | 65018 | ENSG00000158828 | 3732184 | 3066283 | 0,1795 | -0,1932 | -- | -- | | -- |
| Q006404 | PIP3AP | 54545 | ENSG00000150712 | 2842645 | 2103284 | -0,1950 | -0,9363 | -- | DOWN | | -- |
| Q024480 | PIP3AP | 54545 | ENSG00000150712 | 3317960 | 3195091 | -0,0227 | -0,2654 | -- | -- | | -- |
| Q006822 | PIP5K1A | 8394 | ENSG00000143398 | 3328936 | 2807376 | 0,0777 | -0,2510 | -- | -- | | -- |
| Q024898 | PIP5K1A | 8394 | ENSG00000143398 | 3295396 | 4001622 | 0,1163 | 0,2297 | -- | -- | | -- |
| Q006823 | PIP5K1B | 8395 | ENSG00000107242 | 2755428 | 2728030 | -0,3532 | -0,3775 | -- | -- | | -- |
| Q024899 | PIP5K1B | 8395 | ENSG00000107242 | 3693293 | 4008500 | 0,0861 | 0,1447 | -- | -- | | -- |
| Q006966 | PIP5K1C | 23396 | ENSG00000186111 | 2930032 | 1993732 | -0,7288 | -0,9683 | DOWN | DOWN | | DOWN-HIT |
| Q025042 | PIP5K1C | 23396 | ENSG00000186111 | 4973281 | 3438733 | 0,3082 | -0,0626 | -- | -- | | -- |
| Q006701 | PIP5K2A | 5305 | ENSG00000150867 | 3558772 | 3491614 | -0,0766 | -0,2527 | -- | -- | | -- |
| Q024777 | PIP5K2A | 5305 | ENSG00000150867 | 3855748 | 3325486 | -0,0085 | -0,3340 | -- | -- | | -- |
| Q006824 | PIP5K2B | 8396 | ENSG00000141720 | 3640496 | 2146859 | -0,0837 | -0,7664 | -- | -- | | -- |
| Q024900 | PIP5K2B | 8396 | ENSG00000141720 | 3300167 | 2356790 | -0,1959 | -0,7248 | -- | -- | | -- |
| Q007080 | PIP5K2C | 79837 | ENSG00000166908 | 4888629 | 3313221 | 0,3470 | -0,1392 | -- | -- | | -- |
| Q025156 | PIP5K2C | 79837 | ENSG00000166908 | 3552635 | 2971702 | -0,0978 | -0,3226 | -- | -- | | -- |
| Q007143 | PIP5K3 | 200576 | ENSG00000115020 | 3477636 | 3200445 | -0,0002 | -0,1023 | -- | -- | | -- |
| Q025219 | PIP5K3 | 200576 | ENSG00000115020 | 3082812 | 3483177 | -0,2004 | 0,0291 | -- | -- | | -- |
| Q006715 | PKIA | 5569 | ENSG00000171033 | 3505135 | 3372936 | 0,2152 | 0,1747 | -- | -- | | -- |
| Q024791 | PKIA | 5569 | ENSG00000171033 | 3150925 | 2392669 | -0,0234 | -0,3042 | -- | -- | | -- |
| Q006716 | PKIB | 5570 | ENSG00000135549 | 4298893 | 5116605 | 0,3620 | 0,5977 | -- | -- | | -- |
| Q024792 | PKIB | 5570 | ENSG00000135549 | 2416951 | 2429341 | -0,3736 | -0,3334 | -- | -- | | -- |
| Q006936 | PKIG | 11142 | ENSG00000168734 | 3494653 | 3553710 | 0,0685 | -0,0023 | -- | -- | | -- |
| Q025012 | PKIG | 11142 | ENSG00000168734 | 3275980 | 2988567 | 0,0482 | -0,2595 | -- | -- | | -- |
| Q006702 | PKLR | 5313 | ENSG00000143627 | 3392181 | 2701126 | -0,1523 | -0,6580 | -- | -- | | -- |
| Q024778 | PKLR | 5313 | ENSG00000143627 | 2504133 | 2483395 | -0,6367 | -0,7791 | DOWN | -- | | -- |
| Q006703 | PKM2 | 5315 | ENSG00000067225 | 3099849 | 2601925 | -0,2534 | -0,6379 | -- | -- | | -- |
| Q024779 | PKM2 | 5315 | ENSG00000067225 | 3765657 | 3296939 | 0,0127 | -0,2281 | -- | -- | | -- |
| Q006866 | PKMYT1 | 9088 | ENSG00000127564 | 2937538 | 2598482 | -0,5025 | -0,7060 | -- | -- | | -- |
| Q024942 | PKMYT1 | 9088 | ENSG00000127564 | 3194096 | 3145694 | -0,2931 | -0,3428 | -- | -- | | -- |
| Q006729 | PKN1 | 5585 | ENSG00000123143 | 5030997 | 4310681 | 0,3736 | 0,2545 | -- | -- | | -- |
| Q024805 | PKN1 | 5585 | ENSG00000123143 | 1799587 | 1699987 | -1,2224 | -1,0785 | DOWN | DOWN | | DOWN-HIT |
| Q006730 | PKN2 | 5586 | ENSG00000065243 | 4375999 | 3733347 | 0,2860 | 0,1485 | -- | -- | | -- |
| Q024806 | PKN2 | 5586 | ENSG00000065243 | 3826828 | 2539977 | 0,0992 | -0,3532 | -- | -- | | -- |
| Q006998 | PKN3 | 29941 | ENSG00000160447 | 3684354 | 3818783 | -0,0738 | 0,0594 | -- | -- | | -- |
| Q025074 | PKN3 | 29941 | ENSG00000160447 | 4204972 | 3638768 | 0,1462 | -0,0223 | -- | -- | | -- |
| Q006704 | PLAU | 5328 | ENSG00000122861 | 3171930 | 2598519 | -0,1196 | -0,4834 | -- | -- | | -- |
| Q024780 | PLAU | 5328 | ENSG00000122861 | 3681019 | 2525089 | 0,0563 | -0,4631 | -- | -- | | -- |
| Q006705 | PLK1 | 5347 | ENSG00000166851 | 2175273 | 1631663 | -0,3651 | -0,8009 | -- | -- | | -- |
| Q024781 | PLK1 | 5347 | ENSG00000166851 | 2201836 | 1562040 | -0,4573 | -0,7671 | -- | -- | | -- |
| Q006927 | PLK2 | 10769 | ENSG00000145632 | 3490385 | 2676456 | 0,0417 | -0,2576 | -- | -- | | -- |
| Q025003 | PLK2 | 10769 | ENSG00000145632 | 2724561 | 2539351 | -0,1809 | -0,2218 | -- | -- | | -- |
| Q024607 | PLK3 | 1263 | ENSG00000173846 | 3469087 | 3203328 | -0,0991 | -0,2513 | -- | -- | | -- |
| Q006531 | PLK3 | 1263 | ENSG00000173846 | 2295578 | 1806532 | -0,7096 | -1,2042 | DOWN | DOWN | | DOWN-HIT |
| Q006925 | PLK4 | 10733 | ENSG00000142731 | 2306393 | 2033169 | -0,7007 | -0,8150 | DOWN | -- | | -- |
| Q025001 | PLK4 | 10733 | ENSG00000142731 | 3611518 | 2565668 | -0,0399 | -0,4937 | -- | -- | | -- |
| Q006401 | PME-1 | 51400 | ENSG00000189311 | 4238784 | 3991901 | 0,1685 | -0,0136 | -- | -- | | -- |
| Q024477 | PME-1 | 51400 | ENSG00000189311 | 3357917 | 2396971 | -0,1703 | -0,8168 | -- | -- | | -- |
| Q006924 | PMVK | 10654 | ENSG00000163344 | 4184098 | 3245066 | 0,1319 | -0,1745 | -- | -- | | -- |
| Q025000 | PMVK | 10654 | ENSG00000163344 | 4279296 | 3755799 | 0,1624 | -0,0069 | -- | -- | | -- |
| Q007129 | PNCK | 139728 | ENSG00000130822 | 4361615 | 3129706 | 0,0543 | -0,1910 | -- | -- | | -- |
| Q025205 | PNCK | 139728 | ENSG00000130822 | 4975986 | 3875993 | 0,2809 | 0,1284 | -- | -- | | -- |
| Q006708 | PRKAA1 | 5562 | ENSG00000132356 | 3685032 | 4232035 | 0,1563 | 0,3630 | -- | -- | | -- |
| Q024784 | PRKAA1 | 5562 | ENSG00000132356 | 2672132 | 2355991 | -0,3178 | -0,4420 | -- | -- | | -- |
| Q006709 | PRKAA2 | 5563 | ENSG00000162409 | 4651944 | 4225700 | 0,3316 | 0,2634 | -- | -- | | -- |
| Q024785 | PRKAA2 | 5563 | ENSG00000162409 | 4318055 | 3304075 | 0,1258 | -0,1094 | -- | -- | | -- |
| Q006710 | PRKAB1 | 5564 | ENSG00000111725 | 4517582 | 4635766 | 0,2060 | 0,3410 | -- | -- | | -- |
| Q024786 | PRKAB1 | 5564 | ENSG00000111725 | 3758094 | 2286113 | -0,2395 | -0,7352 | -- | -- | | -- |
| Q006711 | PRKAB2 | 5565 | ENSG00000131791 | 4679860 | 4390589 | 0,2307 | 0,2493 | -- | -- | | -- |
| Q024787 | PRKAB2 | 5565 | ENSG00000131791 | 4870088 | 3927837 | 0,1455 | 0,0424 | -- | -- | | -- |
| Q006712 | PRKACA | 5566 | ENSG00000072062 | 4511861 | 4694166 | 0,1914 | 0,3730 | -- | -- | | -- |
| Q024788 | PRKACA | 5566 | ENSG00000072062 | 3856661 | 2670206 | -0,2200 | -0,5476 | -- | -- | | -- |
| Q006713 | PRKACB | 5567 | ENSG00000142875 | 4716575 | 4166787 | 0,3232 | 0,2601 | -- | -- | | -- |
| Q024789 | PRKACB | 5567 | ENSG00000142875 | 2455055 | 1797415 | -0,8540 | -1,0772 | DOWN | DOWN | | DOWN-HIT |
| Q006714 | PRKACG | 5568 | ENSG00000165059 | 4580679 | 4087273 | 0,3893 | 0,3105 | -- | -- | | -- |
| Q024790 | PRKACG | 5568 | ENSG00000165059 | 3534358 | 1958853 | -0,0797 | -0,7665 | -- | -- | | -- |
| Q006717 | PRKAG1 | 5571 | ENSG00000181929 | 4170635 | 4341881 | 0,1653 | 0,2791 | -- | -- | | -- |
| Q024793 | PRKAG1 | 5571 | ENSG00000181929 | 3385563 | 3499629 | -0,1945 | 0,0240 | -- | -- | | -- |
| Q007011 | PRKAG2 | 51422 | ENSG00000106617 | 4605841 | 3542238 | 0,3169 | -0,0540 | -- | -- | | -- |
| Q025087 | PRKAG2 | 51422 | ENSG00000106617 | 3101549 | 3017883 | -0,2102 | -0,3060 | -- | -- | | -- |
| Q007021 | PRKAG3 | 53632 | ENSG00000115592 | 3230204 | 1955633 | -0,2827 | -1,0272 | -- | DOWN | | -- |
| Q025097 | PRKAG3 | 53632 | ENSG00000115592 | 3507735 | 3630814 | -0,1413 | -0,0823 | -- | -- | | -- |
| Q006718 | PRKAR1A | 5573 | ENSG00000108946 | 4459079 | 4288036 | 0,1656 | 0,1999 | -- | -- | | -- |
| Q024794 | PRKAR1A | 5573 | ENSG00000108946 | 3653899 | 3488978 | -0,2454 | -0,0516 | -- | -- | | -- |
| Q006719 | PRKAR1B | 5575 | ENSG00000108946 | 4545671 | 5111158 | 0,1556 | 0,4442 | -- | -- | | -- |
| Q024795 | PRKAR1B | 5575 | ENSG00000108946 | 2610278 | 2228650 | -0,9350 | -0,7452 | DOWN | -- | | -- |
| Q006720 | PRKAR2A | 5576 | ENSG00000114302 | 4988261 | 5138474 | 0,3160 | 0,4767 | -- | -- | | -- |
| Q024796 | PRKAR2A | 5576 | ENSG00000114302 | 5132631 | 3768095 | 0,2891 | 0,0174 | -- | -- | | -- |
| Q006721 | PRKAR2B | 5577 | ENSG00000005249 | 4996725 | 4530126 | 0,3767 | 0,3486 | -- | -- | | -- |
| Q024797 | PRKAR2B | 5577 | ENSG00000005249 | 4673983 | 3457354 | 0,2264 | -0,0712 | -- | -- | | -- |
| Q006722 | PRKCA | 5578 | ENSG00000154229 | 4565421 | 3750065 | 0,3575 | 0,1753 | -- | -- | | -- |
| Q024798 | PRKCA | 5578 | ENSG00000154229 | 3978151 | 3163712 | 0,1228 | -0,0824 | -- | -- | | -- |
| Q006884 | PRKCABP | 9463 | ENSG00000100151 | 2199065 | 2277422 | -1,2693 | -0,7366 | DOWN | -- | | -- |
| Q024960 | PRKCABP | 9463 | ENSG00000100151 | 3343869 | 2981250 | -0,3171 | -0,2583 | -- | -- | | -- |
| Q006723 | PRKCB1 | 5579 | ENSG00000166501 | 3593209 | 3327176 | 0,2298 | 0,1471 | -- | -- | | -- |
| Q024799 | PRKCB1 | 5579 | ENSG00000166501 | 3160306 | 2529820 | 0,0146 | -0,1846 | -- | -- | | -- |
| Q006971 | PRKCBP1 | 23613 | ENSG00000101040 | 3944674 | 3260646 | -0,0266 | -0,1805 | -- | -- | | -- |
| Q025047 | PRKCBP1 | 23613 | ENSG00000101040 | 3915949 | 2940527 | 0,0786 | -0,2797 | -- | -- | | -- |
| Q006724 | PRKCD | 5580 | ENSG00000163932 | 4169747 | 4251425 | 0,3314 | 0,3404 | -- | -- | | -- |
| Q024800 | PRKCD | 5580 | ENSG00000163932 | 2622347 | 2491196 | -0,2217 | -0,2624 | -- | -- | | -- |
| Q007113 | PRKCDBP | 112464 | ENSG00000170955 | 4056134 | 3547079 | 0,0027 | -0,1806 | -- | -- | | -- |
| Q025189 | PRKCDBP | 112464 | ENSG00000170955 | 3112782 | 3578907 | -0,2625 | -0,0916 | -- | -- | | -- |
| Q006725 | PRKCE | 5581 | ENSG00000171132 | 4528091 | 4483971 | 0,2953 | 0,3142 | -- | -- | | -- |
| Q024801 | PRKCE | 5581 | ENSG00000171132 | 2718907 | 2655330 | -0,4590 | -0,3179 | -- | -- | | -- |
| Q006726 | PRKCG | 5582 | ENSG00000126583 | 4328174 | 4950176 | 0,1296 | 0,4010 | -- | -- | | -- |
| Q024802 | PRKCG | 5582 | ENSG00000126583 | 4454737 | 2938156 | 0,1201 | -0,2777 | -- | -- | | -- |
| Q006727 | PRKCH | 5583 | ENSG00000027075 | 4352988 | 4945749 | 0,0944 | 0,3829 | -- | -- | | -- |
| Q024803 | PRKCH | 5583 | ENSG00000027075 | 4287808 | 3034984 | -0,0027 | -0,2785 | -- | -- | | -- |
| Q006728 | PRKCI | 5584 | ENSG00000163558 | 4355179 | 4651445 | 0,0975 | 0,3120 | -- | -- | | -- |
| Q024804 | PRKCI | 5584 | ENSG00000163558 | 3802583 | 2934141 | -0,1615 | -0,3449 | -- | -- | | -- |
| Q006732 | PRKCQ | 5588 | ENSG00000065675 | 3744860 | 3894016 | 0,2079 | 0,2166 | -- | -- | | -- |
| Q024808 | PRKCQ | 5588 | ENSG00000065675 | 2142161 | 1891885 | -0,4085 | -0,5602 | -- | -- | | -- |
| Q006733 | PRKCZ | 5590 | ENSG00000067606 | 4285230 | 4569156 | 0,2425 | 0,3406 | -- | -- | | -- |
| Q024809 | PRKCZ | 5590 | ENSG00000067606 | 4102326 | 3392142 | 0,1728 | 0,0073 | -- | -- | | -- |
| Q006731 | PRKD1 | 5587 | ENSG00000184304 | 3390547 | 3269092 | 0,1598 | 0,1167 | -- | -- | | -- |
| Q024807 | PRKD1 | 5587 | ENSG00000184304 | 3076434 | 2233989 | 0,0105 | -0,2922 | -- | -- | | -- |
| Q006977 | PRKD2 | 25865 | ENSG00000105287 | 1749948 | 1662915 | -0,7798 | -0,8657 | DOWN | -- | | -- |
| Q025053 | PRKD2 | 25865 | ENSG00000105287 | 2454926 | 2097095 | -0,2548 | -0,3693 | -- | -- | | -- |
| Q006974 | PRKD3 | 23683 | ENSG00000115825 | 4700874 | 3508488 | 0,1301 | -0,0883 | -- | -- | | -- |
| Q025050 | PRKD3 | 23683 | ENSG00000115825 | 4031388 | 2439836 | 0,0135 | -0,5206 | -- | -- | | -- |
| Q006734 | PRKDC | 5591 | ENSG00000121031 | 4108214 | 4578274 | 0,0909 | 0,2891 | -- | -- | | -- |
| Q024810 | PRKDC | 5591 | ENSG00000121031 | 4298206 | 3633173 | 0,1081 | 0,0127 | -- | -- | | -- |
| Q006735 | PRKG1 | 5592 | ENSG00000185532 | 4513528 | 4765022 | 0,1936 | 0,3292 | -- | -- | | -- |
| Q024811 | PRKG1 | 5592 | ENSG00000185532 | 4484258 | 3737980 | 0,1140 | 0,0063 | -- | -- | | -- |
| Q006736 | PRKG2 | 5593 | ENSG00000138669 | 4318893 | 4795543 | 0,1053 | 0,3470 | -- | -- | | -- |
| Q024812 | PRKG2 | 5593 | ENSG00000138669 | 4697214 | 4539643 | 0,2152 | 0,2820 | -- | -- | | -- |
| Q006844 | PRKRA | 8575 | ENSG00000180228 | 3774754 | 2780626 | 0,0414 | -0,3215 | -- | -- | | -- |
| Q024920 | PRKRA | 8575 | ENSG00000180228 | 3945626 | 3193890 | 0,1702 | -0,0692 | -- | -- | | -- |
| Q006754 | PRKRIR | 5612 | ENSG00000137492 | 3864140 | 4222862 | 0,1291 | 0,2721 | -- | -- | | -- |
| Q024830 | PRKRIR | 5612 | ENSG00000137492 | 2934776 | 2644723 | -0,1923 | -0,3124 | -- | -- | | -- |
| Q006755 | PRKX | 5613 | ENSG00000183943 | 3306456 | 3221610 | 0,1396 | 0,0658 | -- | -- | | -- |
| Q024831 | PRKX | 5613 | ENSG00000183943 | 2938107 | 2709833 | -0,0154 | -0,0699 | -- | -- | | -- |
| Q006756 | PRKY | 5616 | ENSG00000099725 | 3914584 | 4436207 | 0,2583 | 0,3410 | -- | -- | | -- |
| Q024832 | PRKY | 5616 | ENSG00000099725 | 3333563 | 3302308 | 0,0932 | 0,0442 | -- | -- | | -- |
| Q006859 | PRPF4B | 8899 | ENSG00000112739 | 3702147 | 1575908 | -0,0671 | -1,2810 | -- | DOWN | | -- |
| Q024935 | PRPF4B | 8899 | ENSG00000112739 | 4088298 | 3530318 | 0,1258 | -0,0710 | -- | -- | | -- |
| Q007148 | PRPS1L1 | 221823 | ENSG00000153287 | 4410217 | 3156156 | 0,2823 | -0,0473 | -- | -- | | -- |
| Q025224 | PRPS1L1 | 221823 | ENSG00000153287 | 3121340 | 2908225 | -0,1231 | -0,1053 | -- | -- | | -- |
| Q006757 | PRSS7 | 5651 | ENSG00000154646 | 4056363 | 4570229 | 0,2035 | 0,3157 | -- | -- | | -- |
| Q024833 | PRSS7 | 5651 | ENSG00000154646 | 3204583 | 2366588 | -0,1097 | -0,5361 | -- | -- | | -- |
| Q006758 | PSKH1 | 5681 | ENSG00000159792 | 3470985 | 3634127 | -0,0762 | -0,0619 | -- | -- | | -- |
| Q024834 | PSKH1 | 5681 | ENSG00000159792 | 3405150 | 3113996 | -0,1218 | -0,2439 | -- | -- | | -- |
| Q007106 | PSKH2 | 85481 | ENSG00000147613 | 3657213 | 3070469 | -0,0095 | -0,2021 | -- | -- | | -- |
| Q025182 | PSKH2 | 85481 | ENSG00000147613 | 3952981 | 2827551 | 0,1882 | -0,2484 | -- | -- | | -- |
| Q006369 | PSTPIP1 | 9051 | ENSG00000140368 | 4065658 | 2840360 | 0,1451 | -0,2745 | -- | -- | | -- |
| Q024445 | PSTPIP1 | 9051 | ENSG00000140368 | 2823690 | 2895247 | -0,2921 | -0,1907 | -- | -- | | -- |
| Q006368 | PSTPIP2 | 9050 | ENSG00000152229 | 4889104 | 3746912 | 0,3565 | 0,0591 | -- | -- | | -- |
| Q024444 | PSTPIP2 | 9050 | ENSG00000152229 | 3265806 | 2926039 | -0,2418 | -0,2614 | -- | -- | | -- |
| Q006759 | PTK2 | 5747 | ENSG00000169398 | 3917591 | 4879249 | 0,0702 | 0,3655 | -- | -- | | -- |
| Q024835 | PTK2 | 5747 | ENSG00000169398 | 3330901 | 3185227 | -0,1916 | -0,2439 | -- | -- | | -- |
| Q006575 | PTK2B | 2185 | ENSG00000120899 | 3578322 | 2431481 | 0,1081 | -0,3421 | -- | -- | | -- |
| Q024651 | PTK2B | 2185 | ENSG00000120899 | 3819621 | 3430689 | 0,2021 | 0,1179 | -- | -- | | -- |
| Q006760 | PTK6 | 5753 | ENSG00000101213 | 4201140 | 4516403 | 0,1545 | 0,2506 | -- | -- | | -- |
| Q024836 | PTK6 | 5753 | ENSG00000101213 | 2541863 | 2382333 | -0,5710 | -0,6727 | DOWN | -- | | -- |
| Q006761 | PTK7 | 5754 | ENSG00000112655 | 4655920 | 4226819 | 0,3240 | 0,1836 | -- | -- | | -- |
| Q024837 | PTK7 | 5754 | ENSG00000112655 | 3007793 | 3567615 | -0,2598 | -0,0244 | -- | -- | | -- |
| Q006762 | PTK9 | 5756 | ENSG00000151239 | 4349192 | 4110591 | 0,3123 | 0,2275 | -- | -- | | -- |
| Q024838 | PTK9 | 5756 | ENSG00000151239 | 2891161 | 2554912 | -0,1990 | -0,3589 | -- | -- | | -- |
| Q006946 | PTK9L | 11344 | ENSG00000173366 | 3871291 | 3173551 | -0,0580 | -0,4186 | -- | -- | | -- |
| Q025022 | PTK9L | 11344 | ENSG00000173366 | 2822864 | 2637272 | -0,4451 | -0,6490 | -- | -- | | -- |
| Q025102 | PXK | 54899 | ENSG00000168297 | 2752536 | 3200190 | -0,2787 | -0,2248 | -- | -- | | -- |
| Q007026 | PXK | 54899 | ENSG00000168297 | 1994830 | 1947714 | -0,7602 | -0,9651 | DOWN | DOWN | | DOWN-HIT |
| Q006765 | RAF1 | 5894 | ENSG00000132155 | 3655500 | 4352542 | 0,0544 | 0,2183 | -- | -- | | -- |
| Q024841 | RAF1 | 5894 | ENSG00000132155 | 4446178 | 5237106 | 0,3172 | 0,5318 | -- | -- | | -- |
| Q006764 | RAGE | 5891 | ENSG00000080823 | 3620681 | 4677639 | 0,1380 | 0,3840 | -- | -- | | -- |
| Q024840 | RAGE | 5891 | ENSG00000080823 | 4307580 | 4434570 | 0,3772 | 0,3861 | -- | -- | | -- |
| Q007063 | RBKS | 64080 | ENSG00000171174 | 3935856 | 2697246 | -0,0546 | -0,4487 | -- | -- | | -- |
| Q025139 | RBKS | 64080 | ENSG00000171174 | 4583789 | 4870261 | 0,2238 | 0,3825 | -- | -- | | -- |
| Q006766 | RET | 5979 | ENSG00000165731 | 4491468 | 4807338 | 0,2989 | 0,3358 | -- | -- | | -- |
| Q024842 | RET | 5979 | ENSG00000165731 | 3367084 | 4476631 | -0,1328 | 0,2575 | -- | -- | | -- |
| Q007036 | RFK | 55312 | ENSG00000135002 | 2919698 | 3110325 | -0,3395 | -0,2730 | -- | -- | | -- |
| Q025112 | RFK | 55312 | ENSG00000135002 | 2232548 | 2276992 | -0,5785 | -0,5847 | DOWN | -- | | -- |
| Q007094 | RIOK1 | 83732 | ENSG00000124784 | 2927442 | 3395521 | -0,3816 | -0,1530 | -- | -- | | -- |
| Q025170 | RIOK1 | 83732 | ENSG00000124784 | 3284493 | 3342687 | -0,2119 | -0,1921 | -- | -- | | -- |
| Q007046 | RIOK2 | 55781 | ENSG00000058729 | 3439159 | 3293514 | -0,3829 | -0,1948 | -- | -- | | -- |
| Q025122 | RIOK2 | 55781 | ENSG00000058729 | 2876577 | 2402833 | -0,5040 | -0,5845 | -- | -- | | -- |
| Q006851 | RIOK3 | 8780 | ENSG00000101782 | 3934660 | 2762798 | 0,0513 | -0,3920 | -- | -- | | -- |
| Q024927 | RIOK3 | 8780 | ENSG00000101782 | 2983409 | 2728895 | -0,3194 | -0,4177 | -- | -- | | -- |
| Q006849 | RIPK1 | 8737 | ENSG00000137275 | 3803873 | 2282657 | -0,0046 | -0,7107 | -- | -- | | -- |
| Q024925 | RIPK1 | 8737 | ENSG00000137275 | 4032317 | 2887558 | 0,0762 | -0,4162 | -- | -- | | -- |
| Q006850 | RIPK2 | 8767 | ENSG00000104312 | 4504282 | 4358568 | 0,2325 | 0,2401 | -- | -- | | -- |
| Q024926 | RIPK2 | 8767 | ENSG00000104312 | 3633619 | 3401658 | -0,0793 | -0,1533 | -- | -- | | -- |
| Q006932 | RIPK3 | 11035 | ENSG00000129465 | 2704769 | 2609646 | -0,5172 | -0,5294 | -- | -- | | -- |
| Q025008 | RIPK3 | 11035 | ENSG00000129465 | 4159404 | 3799066 | 0,1202 | 0,0070 | -- | -- | | -- |
| Q007023 | RIPK4 | 54101 | ENSG00000183421 | 3668070 | 2955840 | -0,0593 | -0,3170 | -- | -- | | -- |
| Q025099 | RIPK4 | 54101 | ENSG00000183421 | 3103772 | 3314235 | -0,2344 | -0,1243 | -- | -- | | -- |
| Q006976 | RIPK5 | 25778 | ENSG00000133059 | 4201506 | 2778727 | 0,1260 | -0,3412 | -- | -- | | -- |
| Q025052 | RIPK5 | 25778 | ENSG00000133059 | 3067420 | 1739774 | -0,1841 | -0,7522 | -- | -- | | -- |
| Q006768 | ROCK1 | 6093 | ENSG00000067900 | 4122458 | 4728467 | 0,1447 | 0,3058 | -- | -- | | -- |
| Q024844 | ROCK1 | 6093 | ENSG00000067900 | 3514795 | 3370063 | -0,0861 | -0,1718 | -- | -- | | -- |
| Q006887 | ROCK2 | 9475 | ENSG00000134318 | 3944068 | 2660613 | 0,0983 | -0,3678 | -- | -- | | -- |
| Q024963 | ROCK2 | 9475 | ENSG00000134318 | 2771417 | 2217043 | -0,2441 | -0,4154 | -- | -- | | -- |
| Q006656 | ROR1 | 4919 | ENSG00000185483 | 3350051 | 2294346 | -0,0908 | -0,5001 | -- | -- | | -- |
| Q024732 | ROR1 | 4919 | ENSG00000185483 | 2533092 | 2275860 | -0,4423 | -0,4925 | -- | -- | | -- |
| Q006657 | ROR2 | 4920 | ENSG00000169071 | 1871812 | 1652329 | -0,5016 | -0,6485 | -- | -- | | -- |
| Q024733 | ROR2 | 4920 | ENSG00000169071 | 1726927 | 1586705 | -0,6111 | -0,6350 | DOWN | -- | | -- |
| Q006769 | ROS1 | 6098 | ENSG00000047936 | 4310314 | 4214747 | 0,2286 | 0,1671 | -- | -- | | -- |
| Q024845 | ROS1 | 6098 | ENSG00000047936 | 3483721 | 2760064 | -0,0554 | -0,4068 | -- | -- | | -- |
| Q006770 | RPS6KA1 | 6195 | ENSG00000117676 | 4251485 | 4234564 | 0,2881 | 0,2555 | -- | -- | | -- |
| Q024846 | RPS6KA1 | 6195 | ENSG00000117676 | 3523782 | 3434090 | 0,0446 | 0,0155 | -- | -- | | -- |
| Q006771 | RPS6KA2 | 6196 | ENSG00000071242 | 3475323 | 3425898 | 0,2042 | 0,1255 | -- | -- | | -- |
| Q024847 | RPS6KA2 | 6196 | ENSG00000071242 | 3619830 | 2633766 | 0,2043 | -0,1312 | -- | -- | | -- |
| Q006772 | RPS6KA3 | 6197 | ENSG00000177189 | 3816469 | 4323573 | 0,1744 | 0,2243 | -- | -- | | -- |
| Q024848 | RPS6KA3 | 6197 | ENSG00000177189 | 3294856 | 3512832 | -0,0226 | 0,0052 | -- | -- | | -- |
| Q006861 | RPS6KA4 | 8986 | ENSG00000162302 | 3396169 | 2431439 | -0,0266 | -0,4122 | -- | -- | | -- |
| Q024937 | RPS6KA4 | 8986 | ENSG00000162302 | 3216538 | 2978203 | -0,0077 | -0,0789 | -- | -- | | -- |
| Q006876 | RPS6KA5 | 9252 | ENSG00000100784 | 3026864 | 2868079 | -0,7197 | -0,4489 | DOWN | -- | | -- |
| Q024952 | RPS6KA5 | 9252 | ENSG00000100784 | 4475311 | 2834889 | 0,1158 | -0,3802 | -- | -- | | -- |
| Q025066 | RPS6KA6 | 27330 | ENSG00000072133 | 4907564 | 4685130 | 0,3614 | 0,3595 | -- | -- | | -- |
| Q006990 | RPS6KA6 | 27330 | ENSG00000072133 | 1914893 | 1843517 | -1,1104 | -0,9950 | DOWN | DOWN | | DOWN-HIT |
| Q006773 | RPS6KB1 | 6198 | ENSG00000108443 | 4268368 | 4129557 | 0,2506 | 0,0919 | -- | -- | | -- |
| Q024849 | RPS6KB1 | 6198 | ENSG00000108443 | 4042186 | 3387695 | 0,1448 | -0,1693 | -- | -- | | -- |
| Q006774 | RPS6KB2 | 6199 | ENSG00000175634 | 4284355 | 4822867 | 0,2203 | 0,3006 | -- | -- | | -- |
| Q024850 | RPS6KB2 | 6199 | ENSG00000175634 | 2578042 | 2520280 | -0,5602 | -0,7096 | DOWN | -- | | -- |
| Q006984 | RPS6KC1 | 26750 | ENSG00000136643 | 2725495 | 2237381 | -0,4438 | -0,6104 | -- | -- | | -- |
| Q025060 | RPS6KC1 | 26750 | ENSG00000136643 | 2256077 | 1960324 | -0,5442 | -0,5839 | DOWN | -- | | -- |
| Q007093 | RPS6KL1 | 83694 | ENSG00000198208 | 3171273 | 2667477 | -0,2096 | -0,4848 | -- | -- | | -- |
| Q025169 | RPS6KL1 | 83694 | ENSG00000198208 | 4154408 | 1890580 | 0,1934 | -1,0163 | -- | DOWN | | -- |
| Q006775 | RYK | 6259 | ENSG00000163785 | 3490086 | 4688075 | -0,0941 | 0,2491 | -- | -- | | -- |
| Q024851 | RYK | 6259 | ENSG00000163785 | 3020408 | 3156678 | -0,3458 | -0,3632 | -- | -- | | -- |
| Q006348 | SBF1 | 6305 | ENSG00000100241 | 2923774 | 3029456 | -0,1398 | -0,1126 | -- | -- | | -- |
| Q024424 | SBF1 | 6305 | ENSG00000100241 | 2367631 | 2059323 | -0,4219 | -0,6119 | -- | -- | | -- |
| Q007161 | SBK1 | 388228 | NA | 3778193 | 3434213 | -0,0257 | -0,1589 | -- | -- | | -- |
| Q025237 | SBK1 | 388228 | NA | 5084817 | 4232611 | 0,3472 | 0,1711 | -- | -- | | -- |
| C | Scrambled control | Scrambl | NA | 3769493 | 4103767 | 0,0491 | 0,0735 | -- | -- | | -- |
| C | Scrambled control | Scrambl | NA | 3204481 | 3411956 | -0,1996 | -0,2465 | -- | -- | | -- |
| C | Scrambled control | Scrambl | NA | 3970530 | 3863937 | 0,1699 | 0,0462 | -- | -- | | -- |
| C | Scrambled control | Scrambl | NA | 3687253 | 3670476 | 0,0523 | -0,0564 | -- | -- | | -- |
| C | Scrambled control | Scrambl | NA | 3457095 | 3893727 | -0,1236 | 0,0143 | -- | -- | | -- |
| C | Scrambled control | Scrambl | NA | 3518326 | 3329871 | -0,1335 | -0,2749 | -- | -- | | -- |
| C | Scrambled control | Scrambl | NA | 3804344 | 3601812 | 0,0568 | -0,0260 | -- | -- | | -- |
| C | Scrambled control | Scrambl | NA | 3415464 | 3309457 | -0,1295 | -0,1893 | -- | -- | | -- |
| C | Scrambled control | Scrambl | NA | 3657285 | 3837305 | -0,1586 | -0,0669 | -- | -- | | -- |
| C | Scrambled control | Scrambl | NA | 3512644 | 3238176 | -0,2711 | -0,4131 | -- | -- | | -- |
| C | Scrambled control | Scrambl | NA | 3703116 | 3695000 | -0,0913 | -0,0668 | -- | -- | | -- |
| C | Scrambled control | Scrambl | NA | 3599645 | 3318519 | -0,1814 | -0,2973 | -- | -- | | -- |
| C | Scrambled control | Scrambl | NA | 3870717 | 3599346 | 0,0780 | -0,0732 | -- | -- | | -- |
| C | Scrambled control | Scrambl | NA | 3182869 | 3352673 | -0,2190 | -0,2286 | -- | -- | | -- |
| C | Scrambled control | Scrambl | NA | 3382821 | 3419728 | -0,0622 | -0,0880 | -- | -- | | -- |
| C | Scrambled control | Scrambl | NA | 3079788 | 3289138 | -0,2108 | -0,1907 | -- | -- | | -- |
| C | Scrambled control | Scrambl | NA | 3643303 | 3545951 | 0,0207 | -0,0963 | -- | -- | | -- |
| C | Scrambled control | Scrambl | NA | 3516966 | 3397724 | -0,0120 | -0,1276 | -- | -- | | -- |
| C | Scrambled control | Scrambl | NA | 3515836 | 3599599 | 0,0212 | -0,0044 | -- | -- | | -- |
| C | Scrambled control | Scrambl | NA | 3113339 | 3223389 | -0,1263 | -0,1335 | -- | -- | | -- |
| C | Scrambled control | Scrambl | NA | 3823143 | 3674534 | 0,0516 | -0,0337 | -- | -- | | -- |
| C | Scrambled control | Scrambl | NA | 3612830 | 3283195 | -0,0244 | -0,1807 | -- | -- | | -- |
| C | Scrambled control | Scrambl | NA | 3737068 | 3367959 | 0,0621 | -0,0805 | -- | -- | | -- |
| C | Scrambled control | Scrambl | NA | 3372135 | 3179707 | -0,0768 | -0,1403 | -- | -- | | -- |
| Q006778 | SGK | 6446 | ENSG00000118515 | 3919750 | 3839471 | 0,1722 | 0,0934 | -- | -- | | -- |
| Q024854 | SGK | 6446 | ENSG00000118515 | 2567697 | 1503347 | -0,4057 | -1,1212 | -- | DOWN | | -- |
| Q006905 | SGK2 | 10110 | ENSG00000101049 | 2076209 | 2217929 | -0,7043 | -0,6603 | DOWN | -- | | -- |
| Q024981 | SGK2 | 10110 | ENSG00000101049 | 3044791 | 3273344 | -0,1692 | -0,1550 | -- | -- | | -- |
| Q006973 | SGKL | 23678 | ENSG00000104205 | 4551622 | 4383623 | 0,0592 | 0,2396 | -- | -- | | -- |
| Q025049 | SGKL | 23678 | ENSG00000104205 | 4284766 | 2827981 | 0,0905 | -0,3408 | -- | -- | | -- |
| Q007001 | SH3KBP1 | 30011 | ENSG00000147010 | 3396967 | 2986490 | 0,0782 | -0,0691 | -- | -- | | -- |
| Q025077 | SH3KBP1 | 30011 | ENSG00000147010 | 3269467 | 2779185 | 0,1276 | -0,0102 | -- | -- | | -- |
| Q007091 | Sharpin | 81858 | ENSG00000179526 | 3568101 | 2645486 | 0,1431 | -0,1955 | -- | -- | | -- |
| Q025167 | Sharpin | 81858 | ENSG00000179526 | 2926372 | 2056768 | -0,0108 | -0,3548 | -- | -- | | -- |
| Q007086 | SKIP | 80309 | ENSG00000153820 | 2674763 | 2636585 | -0,4853 | -0,4852 | -- | -- | | -- |
| Q025162 | SKIP | 80309 | ENSG00000153820 | 4287425 | 4809255 | 0,1803 | 0,3525 | -- | -- | | -- |
| Q006403 | SKIP | 51763 | ENSG00000132376 | 3098092 | 2137391 | 0,0136 | -0,5159 | -- | -- | | -- |
| Q024479 | SKIP | 51763 | ENSG00000132376 | 2792084 | 2123530 | -0,1685 | -0,4657 | -- | -- | | -- |
| Q006779 | SKP1A | 6500 | ENSG00000113558 | 3137700 | 3325135 | 0,0678 | 0,0668 | -- | -- | | -- |
| Q024855 | SKP1A | 6500 | ENSG00000113558 | 3174044 | 3027741 | 0,0073 | 0,0033 | -- | -- | | -- |
| Q006780 | SKP2 | 6502 | ENSG00000145604 | 3804381 | 3967302 | 0,1326 | 0,0341 | -- | -- | | -- |
| Q024856 | SKP2 | 6502 | ENSG00000145604 | 3164841 | 2224623 | -0,1621 | -0,7811 | -- | -- | | -- |
| Q006895 | SLK | 9748 | ENSG00000065613 | 3455551 | 2809141 | -0,0479 | -0,2616 | -- | -- | | -- |
| Q024971 | SLK | 9748 | ENSG00000065613 | 2528372 | 2260050 | -0,3170 | -0,3506 | -- | -- | | -- |
| Q025033 | SMG1 | 23049 | ENSG00000157106 | 4775684 | 3070545 | 0,2061 | -0,3076 | -- | -- | | -- |
| Q006957 | SMG1 | 23049 | ENSG00000157106 | 2531905 | 1753132 | -1,0875 | -1,2956 | DOWN | DOWN | | DOWN-HIT |
| Q006363 | SNAP23 | 8773 | ENSG00000092531 | 2458853 | 2107769 | -0,1960 | -0,3108 | -- | -- | | -- |
| Q024439 | SNAP23 | 8773 | ENSG00000092531 | 2260796 | 2035173 | -0,3129 | -0,3456 | -- | -- | | -- |
| Q007090 | SNARK | 81788 | ENSG00000163545 | 4104641 | 3718286 | 0,1985 | 0,1157 | -- | -- | | -- |
| Q025166 | SNARK | 81788 | ENSG00000163545 | 3531684 | 2983658 | 0,0722 | -0,1049 | -- | -- | | -- |
| Q007135 | SNF1LK | 150094 | ENSG00000142178 | 4232799 | 4254978 | 0,2417 | 0,3047 | -- | -- | | -- |
| Q025211 | SNF1LK | 150094 | ENSG00000142178 | 3299953 | 2625301 | -0,1272 | -0,3484 | -- | -- | | -- |
| Q006965 | SNF1LK2 | 23235 | ENSG00000170145 | 5311515 | 3634404 | 0,2762 | -0,0655 | -- | -- | | -- |
| Q025041 | SNF1LK2 | 23235 | ENSG00000170145 | 4911623 | 3176064 | 0,2794 | -0,1926 | -- | -- | | -- |
| Q007024 | SNRK | 54861 | ENSG00000163788 | 3978103 | 3346319 | 0,1272 | -0,0565 | -- | -- | | -- |
| Q025100 | SNRK | 54861 | ENSG00000163788 | 3170726 | 2795240 | -0,0943 | -0,2387 | -- | -- | | -- |
| Q025026 | SORCS3 | 22986 | ENSG00000156395 | 3508387 | 3275818 | -0,0471 | -0,1737 | -- | -- | | -- |
| Q006950 | SORCS3 | 22986 | ENSG00000156395 | 2422916 | 1893612 | -0,7340 | -1,1113 | DOWN | DOWN | | DOWN-HIT |
| Q006424 | SPAP1 | 79368 | ENSG00000132704 | 5323034 | 3883959 | 0,4042 | 0,1279 | -- | -- | | -- |
| Q024500 | SPAP1 | 79368 | ENSG00000132704 | 4675328 | 3804010 | 0,2007 | 0,1137 | -- | -- | | -- |
| Q006858 | SPHK1 | 8877 | ENSG00000176170 | 4293108 | 4372691 | 0,1340 | 0,2032 | -- | -- | | -- |
| Q024934 | SPHK1 | 8877 | ENSG00000176170 | 1720513 | 2185125 | -1,2202 | -0,8556 | DOWN | -- | | -- |
| Q007050 | SPHK2 | 56848 | ENSG00000063176 | 3071869 | 2364281 | -0,3490 | -0,5807 | -- | -- | | -- |
| Q025126 | SPHK2 | 56848 | ENSG00000063176 | 2642761 | 1555464 | -0,4099 | -0,9119 | -- | DOWN | | -- |
| Q006781 | SRC | 6714 | ENSG00000197122 | 4391227 | 4332627 | 0,2674 | 0,1058 | -- | -- | | -- |
| Q024857 | SRC | 6714 | ENSG00000197122 | 3441376 | 2929728 | -0,1513 | -0,5279 | -- | -- | | -- |
| Q006782 | SRMS | 6725 | ENSG00000125508 | 4187642 | 4149365 | 0,1642 | 0,0000 | -- | -- | | -- |
| Q024858 | SRMS | 6725 | ENSG00000125508 | 3218029 | 3504743 | -0,3053 | -0,3036 | -- | -- | | -- |
| Q006783 | SRPK1 | 6732 | ENSG00000096063 | 3878242 | 4231574 | 0,0368 | 0,0248 | -- | -- | | -- |
| Q024859 | SRPK1 | 6732 | ENSG00000096063 | 3764858 | 3350394 | -0,0741 | -0,3881 | -- | -- | | -- |
| Q006784 | SRPK2 | 6733 | ENSG00000135250 | 4065442 | 4308472 | 0,1063 | 0,0738 | -- | -- | | -- |
| Q024860 | SRPK2 | 6733 | ENSG00000135250 | 3522303 | 3361144 | -0,1550 | -0,3340 | -- | -- | | -- |
| Q007097 | SSTK | 83983 | ENSG00000178093 | 3548119 | 2539826 | -0,0869 | -0,5030 | -- | -- | | -- |
| Q025173 | SSTK | 83983 | ENSG00000178093 | 3409086 | 2549631 | -0,0940 | -0,4790 | -- | -- | | -- |
| Q006792 | STK10 | 6793 | ENSG00000072786 | 3558491 | 2480142 | -0,3359 | -0,7254 | -- | -- | | -- |
| Q024868 | STK10 | 6793 | ENSG00000072786 | 2691850 | 1727080 | -0,6246 | -1,1414 | DOWN | DOWN | | DOWN-HIT |
| Q006793 | STK11 | 6794 | ENSG00000118046 | 4364296 | 3484458 | -0,0656 | -0,1930 | -- | -- | | -- |
| Q024869 | STK11 | 6794 | ENSG00000118046 | 3372234 | 2637044 | -0,3457 | -0,5345 | -- | -- | | -- |
| Q007118 | STK11IP | 114790 | ENSG00000144589 | 2892091 | 1758796 | -0,0717 | -0,8150 | -- | -- | | -- |
| Q025194 | STK11IP | 114790 | ENSG00000144589 | 2433421 | 2110152 | -0,2582 | -0,6136 | -- | -- | | -- |
| Q006845 | STK16 | 8576 | ENSG00000115661 | 2584925 | 2539264 | -0,3351 | -0,3263 | -- | -- | | -- |
| Q024921 | STK16 | 8576 | ENSG00000115661 | 1916296 | 1878350 | -0,5823 | -0,5496 | DOWN | -- | | -- |
| Q006879 | STK17A | 9263 | ENSG00000164543 | 3547677 | 2984323 | -0,0764 | -0,2541 | -- | -- | | -- |
| Q024955 | STK17A | 9263 | ENSG00000164543 | 4501156 | 2581352 | 0,3578 | -0,3011 | -- | -- | | -- |
| Q006878 | STK17B | 9262 | ENSG00000081320 | 4856572 | 3329518 | 0,2381 | -0,1676 | -- | -- | | -- |
| Q024954 | STK17B | 9262 | ENSG00000081320 | 3863256 | 4131238 | 0,0070 | 0,2335 | -- | -- | | -- |
| Q006857 | STK19 | 8859 | ENSG00000166301 | 3236755 | 2921931 | -0,2736 | -0,4134 | -- | -- | | -- |
| Q024933 | STK19 | 8859 | ENSG00000166301 | 4013990 | 3262411 | 0,0652 | -0,2477 | -- | -- | | -- |
| Q006972 | STK22B | 23617 | ENSG00000182490 | 3749936 | 2680716 | -0,2125 | -0,4808 | -- | -- | | -- |
| Q025048 | STK22B | 23617 | ENSG00000182490 | 3912997 | 3697523 | -0,0112 | 0,0325 | -- | -- | | -- |
| Q007089 | STK22C | 81629 | ENSG00000162526 | 2793138 | 2598513 | -0,4051 | -0,4466 | -- | -- | | -- |
| Q025165 | STK22C | 81629 | ENSG00000162526 | 4381483 | 3806178 | 0,2708 | 0,1108 | -- | -- | | -- |
| Q007096 | STK22D | 83942 | ENSG00000164216 | 3561079 | 2739499 | -0,1144 | -0,4448 | -- | -- | | -- |
| Q025172 | STK22D | 83942 | ENSG00000164216 | 3285969 | 2745819 | -0,2139 | -0,4595 | -- | -- | | -- |
| Q006983 | STK23 | 26576 | ENSG00000184343 | 4393591 | 3337475 | 0,1332 | -0,1327 | -- | -- | | -- |
| Q025059 | STK23 | 26576 | ENSG00000184343 | 3242471 | 2853163 | -0,1978 | -0,2372 | -- | -- | | -- |
| Q006826 | STK24 | 8428 | ENSG00000102572 | 3565714 | 2376592 | -0,1664 | -0,6279 | -- | -- | | -- |
| Q024902 | STK24 | 8428 | ENSG00000102572 | 2865036 | 2765943 | -0,4764 | -0,4620 | -- | -- | | -- |
| Q006918 | STK25 | 10494 | ENSG00000115694 | 1712024 | 2061143 | -1,0364 | -0,7350 | DOWN | -- | | -- |
| Q024994 | STK25 | 10494 | ENSG00000115694 | 2914252 | 2427753 | -0,2362 | -0,4171 | -- | -- | | -- |
| Q006864 | STK29 | 9024 | ENSG00000174672 | 2736022 | 3047360 | -0,5436 | -0,4256 | DOWN | -- | | -- |
| Q024940 | STK29 | 9024 | ENSG00000174672 | 2487203 | 2544249 | -0,6164 | -0,6665 | DOWN | -- | | -- |
| Q006786 | STK3 | 6788 | ENSG00000104375 | 3774899 | 4300063 | 0,1102 | 0,2235 | -- | -- | | -- |
| Q024862 | STK3 | 6788 | ENSG00000104375 | 2660973 | 2167142 | -0,4095 | -0,7138 | -- | -- | | -- |
| Q007049 | STK31 | 56164 | ENSG00000196335 | 4751154 | 3969130 | 0,1874 | 0,0951 | -- | -- | | -- |
| Q025125 | STK31 | 56164 | ENSG00000196335 | 3268245 | 2736201 | -0,2542 | -0,3177 | -- | -- | | -- |
| Q007144 | STK32A | 202374 | ENSG00000169302 | 4080224 | 4340061 | 0,0857 | 0,2824 | -- | -- | | -- |
| Q025220 | STK32A | 202374 | ENSG00000169302 | 4621647 | 3537484 | 0,2326 | 0,0081 | -- | -- | | -- |
| Q007037 | STK32B | 55351 | ENSG00000152953 | 2987097 | 2557842 | -0,5256 | -0,6177 | -- | -- | | -- |
| Q025113 | STK32B | 55351 | ENSG00000152953 | 4249078 | 3201543 | 0,1560 | -0,1807 | -- | -- | | -- |
| Q007153 | STK32C | 282974 | ENSG00000165752 | 3498822 | 2178267 | -0,1605 | -0,7660 | -- | -- | | -- |
| Q025229 | STK32C | 282974 | ENSG00000165752 | 4567380 | 3090669 | 0,1721 | -0,2403 | -- | -- | | -- |
| Q025151 | STK33 | 65975 | ENSG00000130413 | 3076867 | 2442040 | 0,0559 | -0,1517 | -- | -- | | -- |
| Q007075 | STK33 | 65975 | ENSG00000130413 | 1792917 | 1372461 | -0,6532 | -0,9706 | DOWN | DOWN | | DOWN-HIT |
| Q007131 | STK35 | 140901 | ENSG00000125834 | 3148765 | 2106084 | -0,4047 | -0,6604 | -- | -- | | -- |
| Q025207 | STK35 | 140901 | ENSG00000125834 | 4358023 | 4653271 | 0,1790 | 0,4440 | -- | -- | | -- |
| Q006989 | STK36 | 27148 | ENSG00000163482 | 4383924 | 4014790 | 0,1436 | 0,1254 | -- | -- | | -- |
| Q025065 | STK36 | 27148 | ENSG00000163482 | 3047322 | 3026519 | -0,3939 | -0,3600 | -- | -- | | -- |
| Q006945 | STK38 | 11329 | ENSG00000112079 | 3118285 | 2659001 | -0,2815 | -0,6148 | -- | -- | | -- |
| Q025021 | STK38 | 11329 | ENSG00000112079 | 3862313 | 3313476 | 0,0955 | -0,2321 | -- | -- | | -- |
| Q006954 | STK38L | 23012 | ENSG00000152945 | 3807689 | 3388873 | 0,0082 | -0,1781 | -- | -- | | -- |
| Q025030 | STK38L | 23012 | ENSG00000152945 | 3117049 | 2583485 | -0,1643 | -0,4272 | -- | -- | | -- |
| Q006991 | STK39 | 27347 | ENSG00000198648 | 3730799 | 2904736 | -0,0642 | -0,3216 | -- | -- | | -- |
| Q025067 | STK39 | 27347 | ENSG00000198648 | 3791076 | 3160762 | 0,0555 | -0,1055 | -- | -- | | -- |
| Q006787 | STK4 | 6789 | ENSG00000101109 | 3196138 | 3264935 | 0,0592 | 0,0141 | -- | -- | | -- |
| Q024863 | STK4 | 6789 | ENSG00000101109 | 3204757 | 2415084 | -0,0340 | -0,3108 | -- | -- | | -- |
| Q006790 | STK6 | 6790 | ENSG00000087586 | 3094881 | 2104369 | -0,1607 | -0,8459 | -- | -- | | -- |
| Q024866 | STK6 | 6790 | ENSG00000087586 | 3199222 | 2359968 | -0,0360 | -0,4886 | -- | -- | | -- |
| Q007038 | STYK1 | 55359 | ENSG00000060140 | 4811130 | 3528869 | 0,1374 | -0,1402 | -- | -- | | -- |
| Q025114 | STYK1 | 55359 | ENSG00000060140 | 3508270 | 3036573 | -0,2284 | -0,2837 | -- | -- | | -- |
| Q006795 | SYK | 6850 | ENSG00000165025 | 4304778 | 3134126 | -0,0571 | -0,3272 | -- | -- | | -- |
| Q024871 | SYK | 6850 | ENSG00000165025 | 4122635 | 3786497 | -0,0005 | 0,0321 | -- | -- | | -- |
| Q007088 | T3JAM | 80342 | ENSG00000009790 | 2519187 | 2296377 | -0,5895 | -0,6602 | DOWN | -- | | -- |
| Q025164 | T3JAM | 80342 | ENSG00000009790 | 2641877 | 2694158 | -0,5313 | -0,4713 | DOWN | -- | | -- |
| Q007059 | TAOK1 | 57551 | ENSG00000160551 | 4015348 | 3000170 | 0,2405 | -0,1162 | -- | -- | | -- |
| Q025135 | TAOK1 | 57551 | ENSG00000160551 | 3929976 | 3723319 | 0,3155 | 0,3019 | -- | -- | | -- |
| Q006880 | TAOK2 | 9344 | ENSG00000149930 | 2993930 | 2945556 | -0,1321 | -0,2573 | -- | -- | | -- |
| Q006881 | TAOK2 | 9344 | ENSG00000149930 | 3906586 | 3828697 | 0,0137 | 0,0519 | -- | -- | | -- |
| Q024956 | TAOK2 | 9344 | ENSG00000149930 | 2617723 | 2464296 | -0,2124 | -0,3983 | -- | -- | | -- |
| Q024957 | TAOK2 | 9344 | ENSG00000149930 | 3338032 | 2826046 | -0,0941 | -0,3126 | -- | -- | | -- |
| Q007010 | TAOK3 | 51347 | ENSG00000135090 | 3099495 | 3235669 | -0,1096 | -0,1281 | -- | -- | | -- |
| Q025086 | TAOK3 | 51347 | ENSG00000135090 | 1824237 | 1960651 | -0,7109 | -0,8432 | DOWN | -- | | -- |
| Q006994 | TBK1 | 29110 | ENSG00000183735 | 3574102 | 2817471 | 0,0796 | -0,2914 | -- | -- | | -- |
| Q025070 | TBK1 | 29110 | ENSG00000183735 | 3963061 | 3142552 | 0,2573 | -0,1629 | -- | -- | | -- |
| Q006797 | TEC | 7006 | ENSG00000135605 | 3935529 | 2593232 | 0,0609 | -0,4779 | -- | -- | | -- |
| Q024873 | TEC | 7006 | ENSG00000135605 | 3553036 | 2325245 | 0,0174 | -0,4843 | -- | -- | | -- |
| Q006798 | TEK | 7010 | ENSG00000120156 | 3351721 | 3155988 | -0,0105 | -0,1852 | -- | -- | | -- |
| Q024874 | TEK | 7010 | ENSG00000120156 | 3291340 | 3466552 | 0,0451 | 0,0433 | -- | -- | | -- |
| Q024875 | TESK1 | 7016 | ENSG00000107140 | 2798513 | 2619289 | -0,3570 | -0,4266 | -- | -- | | -- |
| Q006799 | TESK1 | 7016 | ENSG00000107140 | 1180475 | 1723629 | -1,7875 | -1,1463 | DOWN | DOWN | | DOWN-HIT |
| Q006915 | TESK2 | 10420 | ENSG00000070759 | 4527577 | 3409585 | 0,2432 | -0,1076 | -- | -- | | -- |
| Q024991 | TESK2 | 10420 | ENSG00000070759 | 3702715 | 2842126 | -0,0646 | -0,4838 | -- | -- | | -- |
| Q006800 | TGFBR1 | 7046 | ENSG00000106799 | 4124766 | 3665022 | -0,0549 | -0,0541 | -- | -- | | -- |
| Q024876 | TGFBR1 | 7046 | ENSG00000106799 | 3835437 | 2943025 | -0,0304 | -0,3030 | -- | -- | | -- |
| Q006801 | TGFBR2 | 7048 | ENSG00000163513 | 3935384 | 1976607 | -0,2104 | -0,9891 | -- | DOWN | | -- |
| Q024877 | TGFBR2 | 7048 | ENSG00000163513 | 3076710 | 2673448 | -0,4528 | -0,4462 | -- | -- | | -- |
| Q006802 | TIE1 | 7075 | ENSG00000066056 | 4807288 | 3792733 | 0,1109 | -0,0010 | -- | -- | | -- |
| Q024878 | TIE1 | 7075 | ENSG00000066056 | 4216732 | 3174458 | 0,0373 | -0,1866 | -- | -- | | -- |
| Q024879 | TK1 | 7083 | ENSG00000167900 | 4259243 | 3129812 | 0,0896 | -0,1803 | -- | -- | | -- |
| Q006803 | TK1 | 7083 | ENSG00000167900 | 3058624 | 1703805 | -0,6058 | -1,1996 | DOWN | DOWN | | DOWN-HIT |
| Q006804 | TK2 | 7084 | ENSG00000166548 | 3011956 | 2312413 | -0,4938 | -0,6772 | -- | -- | | -- |
| Q024880 | TK2 | 7084 | ENSG00000166548 | 3589003 | 2432611 | -0,0804 | -0,4577 | -- | -- | | -- |
| Q006899 | TLK1 | 9874 | ENSG00000198586 | 4214540 | 2696430 | 0,0571 | -0,4527 | -- | -- | | -- |
| Q024975 | TLK1 | 9874 | ENSG00000198586 | 4123452 | 4051345 | 0,0701 | 0,1015 | -- | -- | | -- |
| Q006931 | TLK2 | 11011 | ENSG00000146872 | 3452183 | 3000398 | -0,1575 | -0,3365 | -- | -- | | -- |
| Q025007 | TLK2 | 11011 | ENSG00000146872 | 4467533 | 4383084 | 0,2273 | 0,2123 | -- | -- | | -- |
| Q006956 | TNIK | 23043 | ENSG00000154310 | 4140714 | 3334056 | -0,1271 | -0,2622 | -- | -- | | -- |
| Q025032 | TNIK | 23043 | ENSG00000154310 | 2970092 | 1828959 | -0,5205 | -1,0805 | -- | DOWN | | -- |
| Q006848 | TNK1 | 8711 | ENSG00000174292 | 3043637 | 3131042 | -0,2874 | -0,2449 | -- | -- | | -- |
| Q024924 | TNK1 | 8711 | ENSG00000174292 | 3473906 | 3483756 | -0,0963 | -0,1133 | -- | -- | | -- |
| Q006908 | TNK2 | 10188 | ENSG00000061938 | 2716379 | 3249089 | -0,5381 | -0,1764 | DOWN | -- | | -- |
| Q024984 | TNK2 | 10188 | ENSG00000061938 | 2220732 | 2373940 | -0,8588 | -0,7165 | DOWN | -- | | -- |
| Q007006 | TNNI3K | 51086 | ENSG00000178961 | 2699313 | 2731048 | -0,4953 | -0,4114 | -- | -- | | -- |
| Q025082 | TNNI3K | 51086 | ENSG00000178961 | 4339071 | 3577979 | 0,1984 | -0,0506 | -- | -- | | -- |
| Q007114 | TP53RK | 112858 | ENSG00000172315 | 4032190 | 3026994 | 0,0704 | -0,3097 | -- | -- | | -- |
| Q025190 | TP53RK | 112858 | ENSG00000172315 | 3991285 | 3701708 | 0,1789 | 0,0610 | -- | -- | | -- |
| Q006986 | TPK1 | 27010 | ENSG00000196511 | 2384907 | 2101788 | -0,4330 | -0,6878 | -- | -- | | -- |
| Q025062 | TPK1 | 27010 | ENSG00000196511 | 2845860 | 2340409 | -0,1640 | -0,5587 | -- | -- | | -- |
| Q006935 | TRAD | 11139 | ENSG00000160145 | 3799533 | 3298473 | 0,1363 | -0,0033 | -- | -- | | -- |
| Q025011 | TRAD | 11139 | ENSG00000160145 | 4013857 | 3193776 | 0,2834 | 0,0261 | -- | -- | | -- |
| Q006805 | TRIO | 7204 | ENSG00000038382 | 3703313 | 2463802 | -0,0036 | -0,4938 | -- | -- | | -- |
| Q024881 | TRIO | 7204 | ENSG00000038382 | 3411811 | 2473853 | 0,0043 | -0,3157 | -- | -- | | -- |
| Q007062 | TSKS | 60385 | ENSG00000126467 | 3406891 | 2758377 | -0,2459 | -0,4183 | -- | -- | | -- |
| Q025138 | TSKS | 60385 | ENSG00000126467 | 3768918 | 3414140 | -0,0439 | -0,1522 | -- | -- | | -- |
| Q007101 | TTBK1 | 84630 | ENSG00000146216 | 3780585 | 2865074 | -0,0046 | -0,4464 | -- | -- | | -- |
| Q025177 | TTBK1 | 84630 | ENSG00000146216 | 4684616 | 3332689 | 0,3497 | -0,1978 | -- | -- | | -- |
| Q007132 | TTBK2 | 146057 | ENSG00000128881 | 4600191 | 3634128 | 0,2832 | 0,1461 | -- | -- | | -- |
| Q025208 | TTBK2 | 146057 | ENSG00000128881 | 4079260 | 2816116 | 0,1629 | -0,1667 | -- | -- | | -- |
| Q006806 | TTK | 7272 | ENSG00000112742 | 2721177 | 2473040 | -0,2285 | -0,4735 | -- | -- | | -- |
| Q024882 | TTK | 7272 | ENSG00000112742 | 2906306 | 2844128 | -0,0723 | -0,2096 | -- | -- | | -- |
| Q006807 | TXK | 7294 | ENSG00000074966 | 4142975 | 3328991 | 0,1163 | -0,1332 | -- | -- | | -- |
| Q024883 | TXK | 7294 | ENSG00000074966 | 3387560 | 3567747 | -0,0595 | 0,0145 | -- | -- | | -- |
| Q006808 | TYK2 | 7297 | ENSG00000105397 | 3694230 | 3232417 | -0,1887 | -0,2031 | -- | -- | | -- |
| Q024884 | TYK2 | 7297 | ENSG00000105397 | 3552487 | 3400442 | -0,1167 | -0,0846 | -- | -- | | -- |
| Q006809 | TYRO3 | 7301 | ENSG00000092445 | 4218278 | 3794260 | -0,0506 | 0,0272 | -- | -- | | -- |
| Q024885 | TYRO3 | 7301 | ENSG00000092445 | 2650993 | 2971325 | -0,6546 | -0,2777 | DOWN | -- | | -- |
| Q006810 | TYROBP | 7305 | ENSG00000011600 | 4792781 | 2910868 | 0,1447 | -0,3573 | -- | -- | | -- |
| Q024886 | TYROBP | 7305 | ENSG00000011600 | 3353033 | 2798949 | -0,2945 | -0,3450 | -- | -- | | -- |
| Q007092 | UCK1 | 83549 | ENSG00000130717 | 1457471 | 2215728 | -1,0249 | -0,6695 | DOWN | -- | | -- |
| Q025168 | UCK1 | 83549 | ENSG00000130717 | 2867342 | 2693069 | -0,1768 | -0,4116 | -- | -- | | -- |
| Q006812 | UCK2 | 7371 | ENSG00000143179 | 3460700 | 2495857 | -0,2288 | -0,5332 | -- | -- | | -- |
| Q024888 | UCK2 | 7371 | ENSG00000143179 | 4825381 | 3463283 | 0,3859 | 0,0511 | -- | -- | | -- |
| Q025103 | UCKL1 | 54963 | ENSG00000198276 | 3350243 | 3218451 | -0,1384 | -0,2738 | -- | -- | | -- |
| Q007027 | UCKL1 | 54963 | ENSG00000198276 | 2316884 | 2069102 | -0,7355 | -1,0153 | DOWN | DOWN | | DOWN-HIT |
| Q006811 | UGP2 | 7360 | ENSG00000169764 | 4261658 | 2349545 | 0,0022 | -0,6737 | -- | -- | | -- |
| Q024887 | UGP2 | 7360 | ENSG00000169764 | 4710949 | 3266203 | 0,2782 | -0,0934 | -- | -- | | -- |
| Q007126 | UHMK1 | 127933 | ENSG00000152332 | 3737931 | 3476556 | 0,2638 | 0,1047 | -- | -- | | -- |
| Q025202 | UHMK1 | 127933 | ENSG00000152332 | 2930470 | 3410211 | -0,0217 | 0,0730 | -- | -- | | -- |
| Q006825 | ULK1 | 8408 | ENSG00000177169 | 3847200 | 3168016 | -0,0457 | -0,2190 | -- | -- | | -- |
| Q024901 | ULK1 | 8408 | ENSG00000177169 | 4436281 | 4284731 | 0,1874 | 0,1627 | -- | -- | | -- |
| Q006894 | ULK2 | 9706 | ENSG00000083290 | 3304642 | 2456569 | -0,2720 | -0,5441 | -- | -- | | -- |
| Q024970 | ULK2 | 9706 | ENSG00000083290 | 4448456 | 3518014 | 0,2854 | 0,0804 | -- | -- | | -- |
| Q006978 | ULK3 | 25989 | ENSG00000140474 | 3244583 | 3246621 | -0,0867 | -0,1160 | -- | -- | | -- |
| Q025054 | ULK3 | 25989 | ENSG00000140474 | 3185425 | 2472826 | -0,0359 | -0,4634 | -- | -- | | -- |
| Q007028 | ULK4 | 54986 | NA | 3208724 | 2264419 | -0,3351 | -0,9376 | -- | DOWN | | -- |
| Q025104 | ULK4 | 54986 | NA | 2606031 | 2054718 | -0,5823 | -1,0367 | DOWN | DOWN | | DOWN-HIT |
| Q007016 | UMP-CMPK | 51727 | ENSG00000162368 | 2296746 | 2054324 | -0,5648 | -0,6666 | DOWN | -- | | -- |
| Q025092 | UMP-CMPK | 51727 | ENSG00000162368 | 3073962 | 2119599 | -0,1140 | -0,5494 | -- | -- | | -- |
| Q007136 | WDSAM1 | 151525 | ENSG00000196151 | 4195008 | 3095744 | 0,0898 | -0,1811 | -- | -- | | -- |
| Q025212 | WDSAM1 | 151525 | ENSG00000196151 | 4991204 | 3653883 | 0,3385 | 0,0668 | -- | -- | | -- |
| Q006815 | WEE1 | 7465 | ENSG00000166483 | 2199781 | 2493402 | -0,7100 | -0,5160 | DOWN | -- | | -- |
| Q024891 | WEE1 | 7465 | ENSG00000166483 | 3356520 | 3737051 | -0,0527 | 0,0652 | -- | -- | | -- |
| Q007070 | WNK1 | 65125 | ENSG00000060237 | 3228102 | 3229907 | -0,2623 | -0,1882 | -- | -- | | -- |
| Q025146 | WNK1 | 65125 | ENSG00000060237 | 3973775 | 4066880 | 0,0467 | 0,0745 | -- | -- | | -- |
| Q007074 | WNK2 | 65268 | ENSG00000165238 | 4351488 | 4021595 | 0,2505 | 0,2036 | -- | -- | | -- |
| Q025150 | WNK2 | 65268 | ENSG00000165238 | 4093513 | 3097835 | 0,2648 | -0,0260 | -- | -- | | -- |
| Q007073 | WNK3 | 65267 | ENSG00000196632 | 3357949 | 3021977 | -0,1974 | -0,2610 | -- | -- | | -- |
| Q025149 | WNK3 | 65267 | ENSG00000196632 | 3822035 | 2880787 | 0,0762 | -0,2358 | -- | -- | | -- |
| Q007072 | WNK4 | 65266 | ENSG00000126562 | 2768738 | 2807606 | -0,5129 | -0,3864 | -- | -- | | -- |
| Q025148 | WNK4 | 65266 | ENSG00000126562 | 2238980 | 2551762 | -0,8009 | -0,5368 | DOWN | -- | | -- |
| Q006813 | VRK1 | 7443 | ENSG00000100749 | 4063872 | 3523826 | 0,1548 | 0,0276 | -- | -- | | -- |
| Q024889 | VRK1 | 7443 | ENSG00000100749 | 4115628 | 3233688 | 0,2844 | 0,0599 | -- | -- | | -- |
| Q006814 | VRK2 | 7444 | ENSG00000028116 | 3563958 | 3344853 | 0,1342 | -0,0359 | -- | -- | | -- |
| Q024890 | VRK2 | 7444 | ENSG00000028116 | 2509517 | 2891772 | -0,2143 | -0,1934 | -- | -- | | -- |
| Q007008 | VRK3 | 51231 | ENSG00000105053 | 3250023 | 2975797 | -0,1150 | -0,1839 | -- | -- | | -- |
| Q025084 | VRK3 | 51231 | ENSG00000105053 | 2866072 | 2739716 | -0,1926 | -0,2000 | -- | -- | | -- |
| Q006901 | XYLB | 9942 | ENSG00000093217 | 4148004 | 3396398 | 0,0451 | -0,1160 | -- | -- | | -- |
| Q024977 | XYLB | 9942 | ENSG00000093217 | 3615336 | 2897892 | -0,0685 | -0,2680 | -- | -- | | -- |
| Q006816 | YES1 | 7525 | ENSG00000176105 | 4293974 | 3685750 | 0,1029 | 0,0030 | -- | -- | | -- |
| Q024892 | YES1 | 7525 | ENSG00000176105 | 4713272 | 4756139 | 0,3169 | 0,3717 | -- | -- | | -- |
| Q007019 | ZAK | 51776 | ENSG00000091436 | 4486917 | 3135249 | 0,2560 | -0,2752 | -- | -- | | -- |
| Q025095 | ZAK | 51776 | ENSG00000091436 | 3945323 | 4247150 | 0,1134 | 0,1812 | -- | -- | | -- |
| Q006817 | ZAP70 | 7535 | ENSG00000115085 | 4734730 | 3060596 | 0,2001 | -0,2718 | -- | -- | | -- |
| Q024893 | ZAP70 | 7535 | ENSG00000115085 | 4813432 | 3797401 | 0,2984 | 0,0329 | -- | -- | | -- |
| Q007013 | ZC3HC1 | 51530 | ENSG00000091732 | 2477170 | 3206646 | -0,6229 | -0,2099 | DOWN | -- | | -- |
| Q025089 | ZC3HC1 | 51530 | ENSG00000091732 | 3043445 | 2984730 | -0,3499 | -0,3632 | -- | -- | | -- |

**Supplemental Table S2: List of antiproliferative hits and used siRNAs**

|  |  | **siRNA catalogue number** | | | |
| --- | --- | --- | --- | --- | --- |
|  | **Gene symbol** | **siRNA used in the primary screen** | | **siRNA used in the validation** | |
|  |  | **siRNA1** | **siRNA2** | **siRNA3** | **siRNA4** |
| 1 | AAK1 | SI02224579 | SI02224586 | SI00108717 | SI00108724 |
| 2 | ACVR1B | SI02622046 | SI00288127 | SI03070977 | SI03117107 |
| 3 | ACVRL1 | SI02659972 | SI00000154 | SI02758392 | SI04894687 |
| 4 | AK3 | SI02224453 | SI02224460 | SI03084305 | SI03102253 |
| 5 | AKAP9 | SI00080948 | SI03024854 | SI02223963 | SI02223970 |
| 6 | AKT2 | SI00299166 | SI00287672 | SI00299173 | SI00287679 |
| 7 | CAMKIINalpha | SI02224873 | SI02224880 | SI00112882 | SI00112889 |
| 8 | CDK4 | SI00604744 | SI00001428 | SI00299789 | SI00299803 |
| 9 | CDKN2A | SI02659503 | SI02664396 | SI02664403 | SI00299817 |
| 10 | CIB3 | SI02225223 | SI02225230 | SI02647666 | SI02647673 |
| 11 | CIT | SI02224243 | SI00095088 | SI00095074 | SI04438840 |
| 12 | CSNK1G2 | SI00605171 | SI00605178 | SI03073217 | SI03105690 |
| 13 | DGKB | SI02225265 | SI02225272 | SI03043103 | SI03050551 |
| 14 | EPHA2 | SI02223508 | SI04434990 | SI00300181 | SI00300188 |
| 15 | EPHA5 | SI00063686 | SI00063672 | SI02223536 | SI00287511 |
| 16 | EPHA7 | SI00063707 | SI03091851 | SI02223543 | SI02223550 |
| 17 | EPHB2 | SI02642626 | SI02224796 | SI02224789 | SI04026330 |
| 18 | EPHB6 | SI00063805 | SI00063826 | SI02665292 | SI02758441 |
| 19 | FYN | SI02654729 | SI02659545 | SI00605451 | SI03095218 |
| 20 | GRK4 | SI02649920 | SI02622501 | SI02622508 | SI03032092 |
| 21 | HCK | SI02627807 | SI02665320 | SI02659986 | SI02665327 |
| 22 | HIPK1 | SI00288001 | SI00288008 | SI03054233 | SI04903899 |
| 23 | IHPK3 | SI02659846 | SI02659853 | SI04441318 | SI00148092 |
| 24 | ITPKA | SI00605500 | SI00605507 | SI00034482 | SI00034489 |
| 25 | LIMK1 | SI00036057 | SI00605542 | SI00605549 | SI00036064 |
| 26 | MAP2K1IP1 | SI02225027 | SI02225034 | SI00131845 | SI00131859 |
| 27 | MAP3K6 | SI00066955 | SI04438042 | SI00288218 | SI00288225 |
| 28 | MAP3K7IP1 | SI04900049 | SI02660329 | SI02758903 | SI04952311 |
| 29 | MAP4K1 | SI00095130 | SI04438854 | SI02224250 | SI02224257 |
| 30 | MAPK7 | SI00606039 | SI02629445 | SI00606046 | SI03024924 |
| 31 | MAPKAPK2 | SI00068033 | SI00068012 | SI02223697 | SI00288246 |
| 32 | MARCKS | SI00605584 | SI00605591 | SI00036603 | SI04897060 |
| 33 | NEK3 | SI02225293 | SI02225300 | SI04897193 | SI04897193 |
| 34 | PAK2 | SI00039802 | SI00605710 | SI00301077 | SI00301084 |
| 35 | PIP5K1C | SI02224376 | SI00099953 | SI02758966 | SI00099967 |
| 36 | PKN1 | SI00042350 | SI00605962 | SI00605955 | SI00042357 |
| 37 | PLK3 | SI02223466 | SI00059388 | SI02223473 | SI00059395 |
| 38 | PRKACB | SI03063228 | SI03022740 | SI02225468 | SI02225461 |
| 39 | PXK | SI02224817 | SI02224824 | SI00119609 | SI00119616 |
| 40 | RPS6KA6 | SI00106603 | SI04379592 | SI00287609 | SI02659748 |
| 41 | SMG1 | SI02640148 | SI00102753 | SI02622333 | SI00102767 |
| 42 | SORCS3 | SI00108983 | SI02641100 | SI00108990 | SI00108997 |
| 43 | STK10 | SI02224047 | SI02224054 | SI04713569 | SI04713576 |
| 44 | STK33 | SI02660210 | SI02660203 | SI02660217 | SI00139741 |
| 45 | TESK1 | SI02224117 | SI02224124 | SI04379522 | SI00086919 |
| 46 | TK1 | SI00049931 | SI02223193 | SI02223200 | SI02223200 |
| 47 | UCKL1 | SI00288484 | SI00288491 | SI04439939 | SI04439939 |
| 48 | ULK4 | SI04901631 | SI04025490 | SI02812061 | SI02812068 |

**Supplemental Table S3: Validation raw data (the 14 validated hits are highlighted in yellow: see text for details)**

|  |  | **LOESS LOG** | | | |  | **p value (Student t test vs control)** | | | |
| --- | --- | --- | --- | --- | --- | --- | --- | --- | --- | --- |
|  |  | ***siRNA1*** | ***siRNA2*** | ***siRNA3*** | ***siRNA4*** |  | ***siRNA1*** | ***siRNA2*** | ***siRNA3*** | ***siRNA4*** |
| 1 | **AAK1** | -0,470092655 | -0,607118659 | -0,279796166 | 0,707425265 |  | 0,034606263 | 0,047576371 | 0,000774005 | 0,006834795 |
| 2 | **ACVR1B** | -0,196886689 | -0,754839409 | -0,163676237 | 0,331104817 |  | 0,004227997 | 0,029252554 | 0,012103226 | 0,14316953 |
| 3 | **ACVRL1** | 0,092684882 | 0,295592275 | 0,038936179 | -0,88664031 |  | 0,006964831 | 0,144595179 | 0,743496267 | 0,027623624 |
| 4 | **AK3** | 0,957999937 | -1,154341929 | -0,319330364 | 0,250132771 |  | 6,13926E-05 | 0,01661293 | 0,005871305 | 0,188572685 |
| 5 | **AKAP9** | -0,788074395 | -0,705956483 | -0,763658875 | -0,69607014 |  | 2,18E-10 | 0,036402891 | 1,84694E-10 | 0,030678857 |
| 6 | **AKT2** | -2,387587344 | -1,245631258 | -0,855823125 | 0,225581434 |  | 1,7991E-11 | 0,01884097 | 1,55781E-11 | 0,026291476 |
| 7 | **CAMKIINalpha** | -0,076600797 | -0,223749889 | 0,547568284 | 0,686550673 |  | 0,695261756 | 0,282691411 | 0,000852989 | 0,011494531 |
| 8 | **CDK4** | 0,315638592 | -0,030293074 | 0,423657474 | -0,32959729 |  | 2,24115E-05 | 0,702851546 | 0,006041955 | 0,11446051 |
| 9 | **CDKN2A** | -1,387337648 | -0,407043732 | 0,887717695 | 0,041549566 |  | 0,000129906 | 0,069434642 | 4,59631E-06 | 0,399367578 |
| 10 | **CIB3** | 0,581427535 | -0,811386298 | -1,734152031 | -1,21758084 |  | 0,000106364 | 0,026937595 | 1,26933E-07 | 0,012024105 |
| 11 | **CIT** | 0,211905004 | -0,830411656 | -0,576520427 | -0,38294384 |  | 0,029684373 | 0,034674701 | 0,000717691 | 0,077549149 |
| 12 | **CSNK1G2** | 0,288007698 | -0,662102805 | -0,427953328 | 1,022509789 |  | 0,000376662 | 0,044051382 | 0,0013386 | 0,016099426 |
| 13 | **DGKB** | -1,70073577 | 0,824023703 | 0,345611971 | 0,108050583 |  | 7,78029E-08 | 0,012147063 | 0,000340786 | 0,688066095 |
| 14 | **EPHA2** | -0,759275217 | -1,053620181 | -0,439454873 | 1,142685073 |  | 1,5786E-09 | 0,01869657 | 7,59321E-08 | 0,01182452 |
| 15 | **EPHA5** | -0,758346601 | -0,905171413 | 0,108526499 | 1,176984134 |  | 4,91016E-10 | 0,026441215 | 0,789110116 | 0,019021598 |
| 16 | **EPHA7** | -0,075773838 | -1,277596317 | -0,570342365 | 1,185716678 |  | 1,42789E-06 | 0,015103367 | 8,53304E-10 | 0,033322326 |
| 17 | **EPHB2** | -0,414605337 | -0,771254295 | -0,312373023 | 1,015242975 |  | 1,34411E-08 | 0,029547885 | 6,35082E-09 | 0,014638144 |
| 18 | **EPHB6** | -0,194331971 | -0,782979515 | -0,592743394 | 0,553146466 |  | 0,005920027 | 0,027551239 | 4,5887E-10 | 0,039874221 |
| 19 | **FYN** | -0,857706064 | -1,084830128 | -0,200114138 | 2,061386053 |  | 5,72564E-10 | 0,025057784 | 2,26038E-08 | 0,006738087 |
| 20 | **GRK4** | -0,709843144 | -1,280694437 | -0,748436836 | 0,567810118 |  | 8,53589E-05 | 0,015146567 | 6,18218E-07 | 0,013831338 |
| 21 | **HCK** | -0,442832073 | -0,818906378 | -0,486246783 | 0,340835337 |  | 2,46318E-06 | 0,026961694 | 6,97297E-10 | 0,016180457 |
| 22 | **HIPK1** | -1,037551352 | -0,235152772 | -0,192220867 | 0,331953024 |  | 6,31838E-05 | 0,142330679 | 0,003777451 | 0,116643964 |
| 23 | **IHPK3** | -0,595570955 | -1,389109862 | -1,327466192 | 0,556559637 |  | 2,39595E-05 | 0,013281399 | 1,25976E-05 | 0,055030666 |
| 24 | **ITPKA** | -1,563322152 | -1,718696339 | -0,553724253 | 1,001807835 |  | 2,36495E-07 | 0,010202721 | 1,65719E-06 | 0,022332395 |
| 25 | **LIMK1** | -0,786884874 | -0,891844253 | -0,743323523 | 1,046801398 |  | 2,3809E-08 | 0,022464994 | 9,66759E-10 | 0,016561602 |
| 26 | **MAP2K1IP1** | -0,629302242 | 0,314715646 | -0,187128583 | 0,480566693 |  | 0,003068572 | 0,156007615 | 0,204904145 | 0,048358441 |
| 27 | **MAP3K6** | -0,41275787 | -1,672566276 | -0,428563364 | 0,635773133 |  | 5,94479E-08 | 0,010764336 | 1,64821E-09 | 0,12427825 |
| 28 | **MAP3K7IP1** | -0,688220927 | -1,552949546 | -0,325790493 | 1,022749915 |  | 1,52492E-08 | 0,012245973 | 6,90238E-09 | 0,02310996 |
| 29 | **MAP4K1** | -0,307400228 | -0,811716086 | -0,740890297 | 0,731123859 |  | 3,42145E-08 | 0,034043695 | 5,95085E-11 | 0,055452123 |
| 30 | **MAPK7** | -0,430350809 | -1,075170426 | -1,102077479 | 0,917687225 |  | 0,000408718 | 0,018756201 | 0,00011431 | 0,029535402 |
| 31 | **MAPKAPK2** | -1,199829778 | -0,888466734 | -0,639503895 | 0,601668137 |  | 8,81075E-11 | 0,021811061 | 1,69225E-10 | 0,026317117 |
| 32 | **MARCKS** | -0,738644027 | -1,161056004 | -3,335219146 | 0,998735192 |  | 9,27302E-05 | 0,019941902 | 2,34687E-09 | 0,019964154 |
| 33 | **NEK3** | 0,289333549 | 2,041582167 | -0,351585482 | 0,289333549 |  | 0,000750989 | 0,026155384 | 0,017062772 | 0,10196593 |
| 34 | **PAK2** | -1,578073537 | -1,519464485 | -1,030143834 | -2,96458094 |  | 1,70218E-05 | 0,014214106 | 7,89626E-06 | 0,006370615 |
| 35 | **PIP5K1C** | 0,378891337 | 0,518534125 | -0,071232454 | 0,685876788 |  | 0,000757348 | 0,03808083 | 0,072074512 | 0,024086493 |
| 36 | **PKN1** | -0,752844385 | -1,13364549 | -0,200408836 | 2,005635408 |  | 1,44264E-08 | 0,031893646 | 2,03217E-08 | 0,006775651 |
| 37 | **PLK3** | -0,393816393 | 0,689275999 | 0,567326222 | 1,130115959 |  | 0,000549392 | 0,036984443 | 0,003327269 | 0,012622382 |
| 38 | **PRKACB** | -0,76420587 | -0,582809933 | -0,422762418 | -0,60443617 |  | 3,15748E-10 | 0,040164625 | 1,1225E-09 | 0,033972158 |
| 39 | **PXK** | 0,915802378 | 2,185697962 | 0,96505447 | 0,914877244 |  | 6,05691E-05 | 0,008971904 | 6,33772E-05 | 0,051617832 |
| 40 | **RPS6KA6** | 0,213639616 | -0,801865486 | -0,93473572 | 0,469361569 |  | 5,78052E-08 | 0,026213418 | 4,40144E-10 | 0,056864924 |
| 41 | **SMG1** | -0,099608216 | -0,292445054 | -2,544581268 | 1,121946269 |  | 3,0205E-06 | 0,128992274 | 9,86343E-07 | 0,025962994 |
| 42 | **SORCS3** | 0,699433834 | -1,857441248 | 1,978102777 | 0,203387044 |  | 0,005829222 | 0,009950005 | 0,002931384 | 0,113599795 |
| 43 | **STK10** | 0,575195089 | -0,639782697 | -0,003104484 | 0,393816393 |  | 0,008228093 | 0,047152853 | 0,712028226 | 0,070873676 |
| 44 | **STK33** | -0,842356984 | -1,606110438 | -1,266790642 | 1,640837987 |  | 1,47118E-10 | 0,010836704 | 9,64042E-11 | 0,006271015 |
| 45 | **TESK1** | -1,293015006 | -1,025789167 | 0,134555004 | 0,364613854 |  | 1,29915E-06 | 0,027566286 | 0,056755274 | 0,10268407 |
| 46 | **TK1** | 0,441348601 | 0,792071469 | 0,841141653 | 0,490052959 |  | 0,000463411 | 0,012956289 | 0,00017997 | 0,519456625 |
| 47 | **UCKL1** | -0,051606399 | -0,892126532 | 0,906549695 | 1,072006648 |  | 0,588540794 | 0,032984191 | 8,95239E-05 | 0,019435515 |
| 48 | **ULK4** | 0,300189843 | 1,95424266 | 0,359415446 | 0,880197351 |  | 0,002674376 | 0,043335574 | 0,03831425 | 0,038009466 |

**Supplemental Table S4: Features of the thyroid carcinoma cell lines used in this study**

| **Cell line*** | **Histotype** | **Genetic lesions**** |
| --- | --- | --- |
| TPC1 | PTC | RET/PTC1 |
| BCPAP | PTC | BRAF (V600E), TP53 (D259Y) |
| CAL62 | ATC | KRAS (G12R), TP53 (A161D), NF2 (E215STOP), CDKN2A (del471 and del522) |
| 8505C | ATC | BRAF (V600E), TP53 (R248G), NF2 (E129STOP), CDKN2A (del150) |

* All the cells were autenthicated by SNP genotyping

** From COSMIC: catalogue of somatic mutations in cancer: www.sanger.ac.uk/genetics/CGP/

**Supplemental Table S5: List of antiproliferative hits and qRT-PCR primers used in this study**

|  | **Gene symbol** | **Reverse primer sequence (5’-3’)** | **Forward primer sequence (5’-3’)** |
| --- | --- | --- | --- |
| 1 | AAK1 | GGGTTTTGGTTGGGGAAC | AAGAGGGCCACTGTTCAGC |
| 2 | ACVR1B | GGCAGCTGATATTCTTCATGG | ATATTGGGAGATTGCTCGAAGA |
| 3 | ACVRL1 | CTGCTCCGAAGGAGGTTG | CCACCTCTGCAACCACAA |
| 4 | AK3 | GCTTTCTGGCTTCTTTGTGG | TCTCATTCAGCGTGAGGATG |
| 5 | AKAP9 | CTTTGTCGAAACTGGGCAAG | GGAGGACGAGGAGAGACAGA |
| 6 | AKT2 | ACACACCCCAGCTGCCCTCA | CGCCAAGCCCAACCACTCTGG |
| 7 | CAMKIINalpha | GAGGCCAGCAACAGATTCTC | GGAGGGACACCACTACCTGA |
| 8 | CDK4 | AGGGAGACCCTCACGCCAGC | CTCTGCGTCCAGCTGCTCCG |
| 9 | CDKN2A | AAAACCCTCACTCGCGGCGG | GTGCGTGGGTCCCAGTCTGC |
| 10 | CIB3 | TCACGGAAAACATGTCCAAA | CTCGTGCCCCTCGACTATAC |
| 11 | CIT | CAGAAGGAGGTGGAGCTGAA | CTTGCTCCAAACTTCGCTTT |
| 12 | CSNK1G2 | AGTTCAACGCCTGGTTTTTG | CTTCGAGAAGCCCGACTATG |
| 13 | DGKB | CTCCTGAAGTGGGCAGAGTC | GGACTGCATTGTGTTTGGTG |
| 14 | EPHA2 | CCAGGCAGGCTACGAGAA | GGCTCTCAGATGCCTCAAAC |
| 15 | EPHA5 | TCTGGAGGACGTGCCTTCTCCT | TGACAGTGCGTGAATCCAAT |
| 16 | EPHA7 | GGTCAGATGGAGCCCTGTAA | AAGCAGGCTACCAGCAAAAA |
| 17 | EPHB2 | CCAGCAGAACTTGCATCTTG | TCTTTGGAGGGCCTGGAT |
| 18 | EPHB6 | CGGAACTCCTGCTCTATTGC | TGCTGGTGAATAGCCACTTG |
| 19 | FYN | CATCTTTTCGGCCAAGTTTT | CAGCAATTATGTGGCTCCAG |
| 20 | GRK4 | CACAATCACTTCGGTCCTCA | GAGGCACATTGAATTCTTGGA |
| 21 | HCK | GCAATCTGGGCTGAGAAGTC | TGGCAGTGAAGACGATGAAG |
| 22 | HIPK1 | TTTTGTGAAGCTATTGATATGTGGT | GCAAGCCTTGTGTTTGTGAA |
| 23 | IHPK3 | GAGCATACGGTGTGCAAGC | AGAGGTGCACTGTGACGGTA |
| 24 | ITPKA | TACATGTCCTTCCGCAGCTT | GTGTGCTCGACTGCAAAATG |
| 25 | LIMK1 | TGTCCTTGGCAAAGCTCACT | GGGGCATCATCAAGAGCA |
| 26 | MAP2KIP1 | TGTCAGATAGAGATGGAGTACCTGTT | AAACCAGGTCGCAAAGCAT |
| 27 | MAP3K6 | CGGGGGAGATGTTGGAGT | TCTCCTTGATGGCGATGC |
| 28 | MAP3K7IP1 | CTGAGCCAACCCCAGAGA | GGAGCTTGCTGCAGAGTGA |
| 29 | MAP4K1 | GCCACCAAGATGCTCAGTC | CCGGGATTCTTCAGTTTGTC |
| 30 | MAPK7 | GGTCGCTTTCCATCAGGTC | AAATGGCGGACACAATTCC |
| 31 | MAPKAPK2 | TCTCGTACACATCCACGATCC | GAGGACCCAGGAGAAATTCG |
| 32 | MARCKS | TTTACCTTCACGTGGCCATT | ATGGGTGCCCAGTTCTCC |
| 33 | NEK3 | TATTAGGGTGTTTCATTTTGGCTA | GCCATGAAAGAAATAAGGCTTC |
| 34 | PAK2 | GATAACGGTTTGGCCAGTTTC | AAGGGGTTCAGCCAAAGAAT |
| 35 | PIP5K1C | CTGGGTCGGGGGCTGCATAGA | CACCGACATCTACTTTCCCACCG |
| 36 | PKN1 | CTCTTCGATGCGCAGCTC | CACATCCGCATGCAACT |
| 37 | PLK3 | GTGGTCCCCGTAGAAGTTCA | CTACATGGAGCAGCACCTCA |
| 38 | PRKACB | CTTTGGCTAGAAACTCTTTCACG | TGACCCCTTCTTGCCATC |
| 39 | PXK | GGGCCAAGGTCAGCCCAGCTTA | GAACCAAAGTGGGAGGTGGTGGA |
| 40 | RPS6KA6 | GCAACTCAAACTGTGCAGGA | CGGCGAGGTAAATGGTCTTA |
| 41 | SMG1 | GCACTATCAGTTCTGGGTTGCCAGT | TAATGAGCCGCAGAGCCCCGG |
| 42 | SORCS3 | TTCCTCTCAGGTCCACATCC | ACGCGTGGGATTTACTTCAC |
| 43 | STK10 | CTTCGGCAAGGTTTACAAGG | TCAATCTCCACGATGTAGTCCTC |
| 44 | STK33 | TGTAATTGGCATCAGGGACA | CTTCGGTGAGACCAACCAAT |
| 45 | TESK1 | GGTGCTGGGTTCCAGGTT | AACTCTGGTGGGGGATGACT |
| 46 | TK1 | AATGGCTTCCTCTGGAAGGT | AGACACTCGCTAACAGCAGCA |
| 47 | UCKL1 | GGTAAGCTGGTTGTTCTGGA | TTCAGGACTGCGTCGTACAG |
| 48 | ULK4 | ACCCCTCACAACTTCTGGTG | GGCAAAAGTGGAAGGTGAAA |
